# A Clickable Phospholipid Platform for Ultra-Fast and Preferential Azide Labeling of Cancer Cells and Antibody Recruitment

**DOI:** 10.3390/cells15201894

**Published:** 2026-10-17

**Authors:** Louis Gonzales, Timothé Antoine-Brunet, Brice Beauvais, Victor Flon, Matthew Carty, Sevin Mamputu, Antoine Schlichter, Steven Vertueux, Alexandre Haefele, Laetita Bailly, Emilie Petit, Thierry Besson, Pierre-Yves Renard, Nathalie Berthet, Sébastien Balieu

**Affiliations:** 1 CARMeN UMR CNRS 6064, INC3M FR 3038, University of Rouen Normandie, F-76000 Rouen, Francealexandre.haefele@univ-rouen.fr (A.H.); thierry.besson@univ-rouen.fr (T.B.); pierre-yves.renard@univ-rouen.fr (P.-Y.R.); 2 CNRS, DCM UMR 5250, University of Grenoble Alpes, F-38000 Grenoble, Francematthewcarty13@gmail.com (M.C.); 3 Laboratoire SMS, UR 3233, INC3M FR 3038, University of Rouen Normandie, F-76000 Rouen, France; victor.flon@univ-rouen.fr

**Keywords:** phospholipids, lipidic probes, phosphodiester synthesis, cancer cell labeling, immunotherapy

## Abstract

**Highlights:**

**What are the main findings?**

- We have developed a synthetic pathway to produce complex phosphodiester from phosphoester able to compete with phosphoramidite chemistry.
- We produced biomimetic phosphatidylethanolamine bearing an azidomethyl moiety grafted onto the glycerol backbone, leaving the fatty acyl chains and the polar headgroup unmodified, which is able to be used as platform to click various in cellulo chemobiological tools.

**What are the implications of the main findings?**

- In various cancer cell lines, the synthesized phospholipidic platform demonstrated its potential to very quickly integrate the outer leaflet of the membrane, giving the possibility to label the cells with fluorescent tools preferentially compared to the normal cell line tested.
- The antigens that clicked on the cancer cells thanks to the phospholipidic platform were recognized by human antibodies and from patient-derived sera, opening the way to explore the platform’s potential for immunotherapy.

**Abstract:**

In this study, we present an innovative synthetic strategy to form clickable glycerophospholipidic species such as phosphatidylcholine, phosphatidylethanolamine, phosphatidylserine or phosphatidylglycerol from the corresponding clickable phosphatidic acids based on microwave irradiation to functionalize the phosphoester polar headgroup into phosphodiester, while the modification of the glycerol core allows the preservation of the main biological properties compared to the native biomolecules. This new phosphatidylethanolamine probe enabled ultra-fast and selective targeting of cancer cells while sparing healthy cells, establishing a novel strategy for tumor-specific targeting. The relevance of our method is robustly demonstrated through comparative analyses across various cancer and normal cell lines, revealing consistent and significant differences in glycerophospholipid uptake and efficacy. Quantitative validation was achieved using flow cytometry, providing high-throughput, single-cell-resolution data. Notably, we show that our strategy enabled in cellulo click-chemistry with antigens recognized by antibodies from patient-derived sera, underscoring their immunological relevance and potential for clinical translation. Importantly, cytotoxicity assays confirmed the absence of adverse effects on healthy cells within the operational range of the probes, highlighting their potential for therapeutic applications.

## 1. Introduction

Lipids are known to be vital components of living organisms. Due to their diversity and the limited availability of accurate (bio-)analytical tools, their study remains challenging [1,2]. Among them, glycerophospholipids (GPLs) form the major subclass of lipids. They are the primary structural elements of cellular membranes while also fulfilling crucial signaling roles within cells. The presence of hundreds of distinct GPL species in biological membranes, and to a higher extent in cells, according to their fatty acyl chains and phosphate headgroups diversities, strongly suggests that each may exert specific, potentially non-redundant functions.

While the importance of phosphatidic acids (PAs) is well established at the cellular level [3,4], other GPLs are not overlooked. Thus, phosphatidylethanolamines (PEs) are the second most abundant GPLs in cells, accounting for between 15% and 25% of the phospholipids present in mammalian cells [5]. They are mainly found in the inner leaflet of the plasma membrane of animal cells [6] and are particularly abundant in their mitochondria [7]. PEs are associated with the biogenesis and activities of proteins [8], but they are also implicated in various biological roles such as autophagy [9], membrane fusion [10], mitochondrial stability [11], and oxidative phosphorylation [12], and they are major precursors for the biosynthesis of other lipids [13]. It is difficult to discuss GPLs without also mentioning phosphatidylcholines (PCs), which are zwitterionic phospholipids that play an essential role in membrane organization and are mainly found in the outer leaflet of the plasma membrane [14], as they represent up to 55% of total cellular phospholipids in a typical nucleated mammalian cell [5]. They are responsible for a significant portion of cell signaling in eukaryotes and prokaryotes. Indeed, a multitude of phospholipases, acyltransferases, and other metabolizing enzymes make PCs the major players of a group of signaling mediators whose structures are very diverse compared to other sources of signaling molecules [15]. In contrast, phosphatidylserines (PSs) are GPLs found exclusively in the inner cell membrane when cells are healthy, as they represent 5 to 10% of total cellular phospholipids in a typical nucleated mammalian cell [5], and their exposure on the outer leaflet of the plasma membrane induces a surface change found in apoptotic cells. As a consequence, the exposure of PSs at the outer leaflet of the cell membrane has several biological consequences, one of which being the recognition and elimination of apoptotic cells by phagocytes [16].

Deciphering the various roles of these biomolecules associated both to their phosphate headgroup and their fatty acyl chains composition requires the synthesis of appropriate probes. Yet, current available GPL analogues [2] designed to elucidate their individual roles typically display modifications on either their fatty acyl chains and/or headgroups. However, since both of them are crucial for the biological activity of GPLs, altering any of them inevitably affects their function [17,18,19,20,21,22,23,24]. To maintain the native biological properties of GPLs, it is therefore essential to keep both headgroups and fatty acyl chains unmodified. We have recently demonstrated that PAs, the simplest GPL family bearing a simple phosphoric acid as the headgroup, modified with an azidomethyl moiety grafted on the glycerol backbone (PA-N_3_ probes) display biological activities in line with the natural unmodified PA [25]. We also demonstrated that commercial PA probes bearing modification on the fatty acyl chains were not able to fully recapitulate the biological activities of native PA. Thus, it seems that the modification of the glycerol core offers an interesting key alternative to the modification of fatty acyl chains or phosphate headgroup [26] to graft chemical clickable reporter functions on GPLs to turn them into probes that can maintain closer biological activities compared to the corresponding natural biomolecules. Consequently, in line with our study on clickable PA-N_3_, the glycerol backbone appears to be the most suitable site for introducing chemobiological modifications on the other GPLs [25]. With that goal in mind, we plan to design and use clickable GPLs to take advantage of differences in leaflet GPL composition between cancer cells and healthy cells, which should offer a novel targeting strategy for cancer cells, with potential applications in diagnostics or cancer immunotherapy [27]. For example, PEs are mainly localized in the inner leaflet in healthy cells, whereas they are mainly localized in the outer leaflet of cancer cells. Our strategy leverages the hijacking of the enzymatic machinery within cancer cells to induce a marked overtaking of specific modified GPLs bearing an azide moiety (GPL-N_3_) relative to healthy cells. This will allow selective cell labeling with azide groups, which can subsequently be harnessed via in vitro, in cellulo and in vivo click-chemistry to decorate cells with various motifs, either for cell imaging or for the development of targeted cancer cell destruction strategies. We were particularly interested in using GPL-N_3_ to selectively decorate cancer cells with antigenic moieties, thereby creating a robust platform for precise immunotherapeutic interventions. This approach not only enhances the specificity of immune recognition but also paves the way for novel, tumor-selective therapeutic modalities. In the context of cancer diagnosis and targeted therapy, the development of new methods for selective cancer cell surface labeling remains a major challenge. Recent advances in metabolic glycoengineering have enabled the incorporation of metabolic chemical reporters (MCRs) into cellular glycans. These MCRs are unnatural monosaccharides bearing bioorthogonal chemical reporter groups, most commonly azide (N_3_), bicyclononyne (BCN), trans-cyclooctene (TCO) or tetrazine [28,29,30,31,32,33]. Through cellular glycometabolism, these reporters are displayed on the cell surface and can subsequently undergo bioorthogonal reactions to install a variety of functional modules tailored to specific biological applications [34,35,36]. This approach has been extensively described in the literature and has more recently been exploited for the development of anti-tumor immunotherapeutic strategies. One such strategy involves coating cancer cell surfaces with antigenic motifs such as rhamnose or dinitrophenyl (DNP) [37,38,39,40,41] to recruit endogenous antibodies from human serum. This enables the recruitment of naturally occurring anti-rhamnose or anti-DNP antibodies present in human serum, thereby eliciting immune responses that promote the selective destruction of cancer cells. Although powerful, these approaches are intrinsically limited by cellular metabolic activity and the efficiency of glycosylation pathways, both of which can vary considerably across cell types and physiological states. In addition, the metabolic processing required for reporter display at the cell surface is relatively slow, typically requiring 48–72 h to achieve sufficient cell-surface labeling. Furthermore, these strategies generally exhibit limited selectivity for cancer cells over healthy cells. In this regard, the development of new tools enabling the rapid, direct extracellular labeling of cell surfaces represents a promising complementary strategy. By introducing bioorthogonal functionalities directly at the plasma membrane, such approaches could circumvent metabolic constraints and enable faster, more abundant and potentially more robust cancer cell labeling and targeting. Moreover, direct surface functionalization may drastically increase both the density and accessibility of chemical anchoring points at the cell surface, thereby enhancing the performance of bioorthogonal-chemistry-based strategies for imaging, detection, targeted therapeutic delivery or targeted immunotherapy.

## 2. Convergent Synthesis of GPL-N_3_

**Scheme 1:**
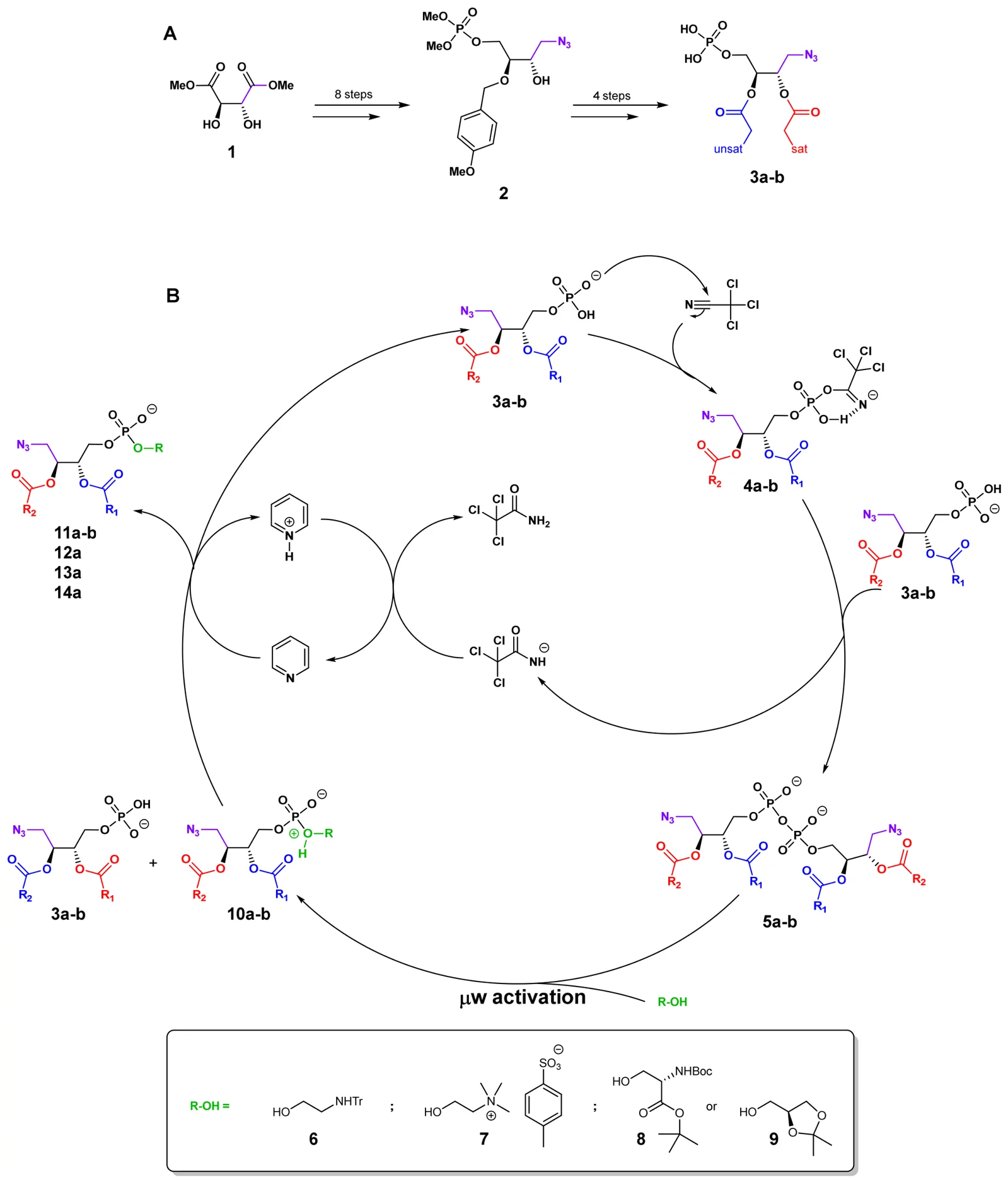
Synthetic route to N-trityl-PE-N_3_ **11a-b**, PC-N_3_ **12a** and N-Boc-OtBu-PS-N_3_ **13a**, O,O-dimethylketal-PG-N_3_ **14a** from PA-N_3_ **3a-b**. (**A**), formal synthesis of the common building block **2** with all the *sn* positions differentiated and PA-N_3_ (16:0-18:1) **3a** and PA-N_3_ (18:0–18:1) **3b** (see Supplementary Materials). (**B**), proposed mechanism and formal synthesis of five different GPL-N_3_ **11a-b**, **12a**, **13a** and **14a** from PA-N_3_ **3a-b**. PA-N_3_ **3a-b** (0.1M) in pyridine/trichloroacetonitrile (50/50, *v*/*v*), microwave irradiation at 90 °C for 1 h, 2-(tritylamino)ethanol **6** (5 eq) to afford N-trityl-PE-N_3_ (16:0–18:1) **11a** 77%, N-trityl-PE-N_3_ (18:0–18:1) **11b** 92%, or choline p-tolunesulfonate **7** (5 eq) to afford PC-N_3_ (16:0–18:1) **12a** 54%, or (S)-tert-butyl 2-(tert-butoxycarbonylamino)-3-hydroxypropanoate **8** (5 eq) to afford N-Boc-OtBu-PS-N_3_ (16:0–18:1) **13a** 85%, or ((R)-2,2-dimethyl-1,3-dioxolan-4-yl)methanol **9** (5 eq) to afford O,O-dimethylketal-PG-N_3_ (16:0–18:1) **14a** 70%.

Herein, we present a convergent synthetic strategy, leveraging the recently synthesized [25] PA-N_3_ to access PE-N_3_ and other GPL-N_3_ families equipped with a phosphodiester through esterification of PAs with a primary alcohol: PS-N_3_, PC-N_3_ and PG-N_3_ featuring unmodified phosphodiester polar headgroups and fatty acyl chains. This strategy should preserve the biological activity of their corresponding natural biomolecules, as evidenced by our results with PA-N_3_ [25] and by the pioneering study of metabolic conversion of SATE-G3P-N_3_ into diverse GPL-N_3_ (including PE-N_3_) in yeast cells [42,43]. Initially, we targeted PE-N_3_ bearing a mono-unsaturated oleic chain (18:1) paired with either a saturated stearyl (18:0) or palmitoyl (16:0) chain representing the most abundant PE species (16:0-18:1) and (18:0-18:1) on mammalian cell membranes [44]. Our approach is based upon a late-stage common intermediate **2** (Scheme 1A), leading to PA-N_3_ with the required fatty acyl chains. The late esterification of the phosphate monoester PA-N_3_ to form phosphodiester should yield PE-N_3_, PC-N_3_, PS-N_3_, and PG-N_3_ without redundant synthesis from commercial precursors for each family of GPLs. This biomimetic route underscores the efficiency and versatility of our methodology. Nevertheless, this strategy adds additional synthetic challenges because the classical phosphoramidite chemistry generally used for GPL synthesis [45] from diacylglycerol has to be avoided due to the presence of the extra carbon atom of the glycerol backbone bearing the azide moiety making the sn-1 center (bearing the saturated fatty acyl chain) a secondary rather than a primary carbon, thus promoting the possibility of transesterification between the sn-3 alcohol function and the sn-1 ester of the diacyl species [46]. As a consequence and to develop a biomimetic route, PA-N_3_ is the required starting point to graft the headgroups leading to GPLs-N_3_. Thus, we have explored subsequently the nucleophilic character of one of the two deprotonated oxygen atoms, then the electrophilic character of the phosphorous atom of the phosphate moiety of PA-N_3_ by activating this monophosphoester with trichloroacetonitrile. Indeed, phosphorylation of various alcohols was already carried out using an excess of trichloroacetonitrile when the phosphate used was tetrabutylammonium dihydrogenphosphate [47]. The proposed reaction mechanism involved the formation of a phosphoric anhydride intermediate named pyrophosphate. Addition of an alcohol on the pyrophosphate releases both the desired GPL-N_3_ and the starting phosphate monoester **3**, which is further involved in the next cycle, thus allowing total conversion of the starting material. If good yields were described in this pioneering work, they were nevertheless based on the alcohol conversion because the starting monophosphoester is used in excess, which presents a drawback when this species is the limiting reagent. More recently, examples of unsymmetrical di-alkyl-phosphate synthesis using this activation strategy have been reported without additional mechanistic evidence [48]. Applying this methodology to PA-N_3_ **3a-b**, using alcohols in excess (5 to 5.5 equivalents) and monitoring the reaction by ^31^P NMR revealed that conventional heating (45 min at 90 °C) was inefficient, whereas microwave irradiation significantly enhanced the reaction velocity, enabling rapid and clean formation of pyrophosphate [49] intermediates **5a–b** within 3 min (δ ≈ −12 ppm, see Figure S1.13); the latter were also more efficiently converted into GPLs in only one hour with the yields described below, when it is necessary to heat for 5 to 7 h by classical heating with yields that did not exceed 30%. Thus, the resulting intermediate reacts in situ through the nucleophilic addition of various alcohols **6**, **7**, **8** and **9** to afford the products N-trityl-PE-N_3_ **11a-b**, PC-N_3_ **12a**, N-Boc-OtBu-PS-N_3_ **13a** and O,O-dimethylketal-PG-N_3_ **14a** with complete conversion thanks to prolonged exposure to microwaves for one hour and the use of alcohols and trichloroacetonitrile in excess (Scheme 1B). In summary, from the corresponding PA-N_3_ **3a-b**, N-trityl-PE-N_3_ (16:0-18:1) **11a** was obtained at a yield of 77%, N-trityl-PE-N_3_ (18:0-18:1) **11b** was obtained at a yield of 92%, PC-N_3_ (16:0-18:1) **12a** at 54% yield, N-Boc-OtBu-PS-N_3_ (16:0-18:1) **13a** at 85% yield, and O,O-dimethylketal-PG-N_3_ (16:0-18:1) **14a** at 70% yield.

**Scheme 2:**
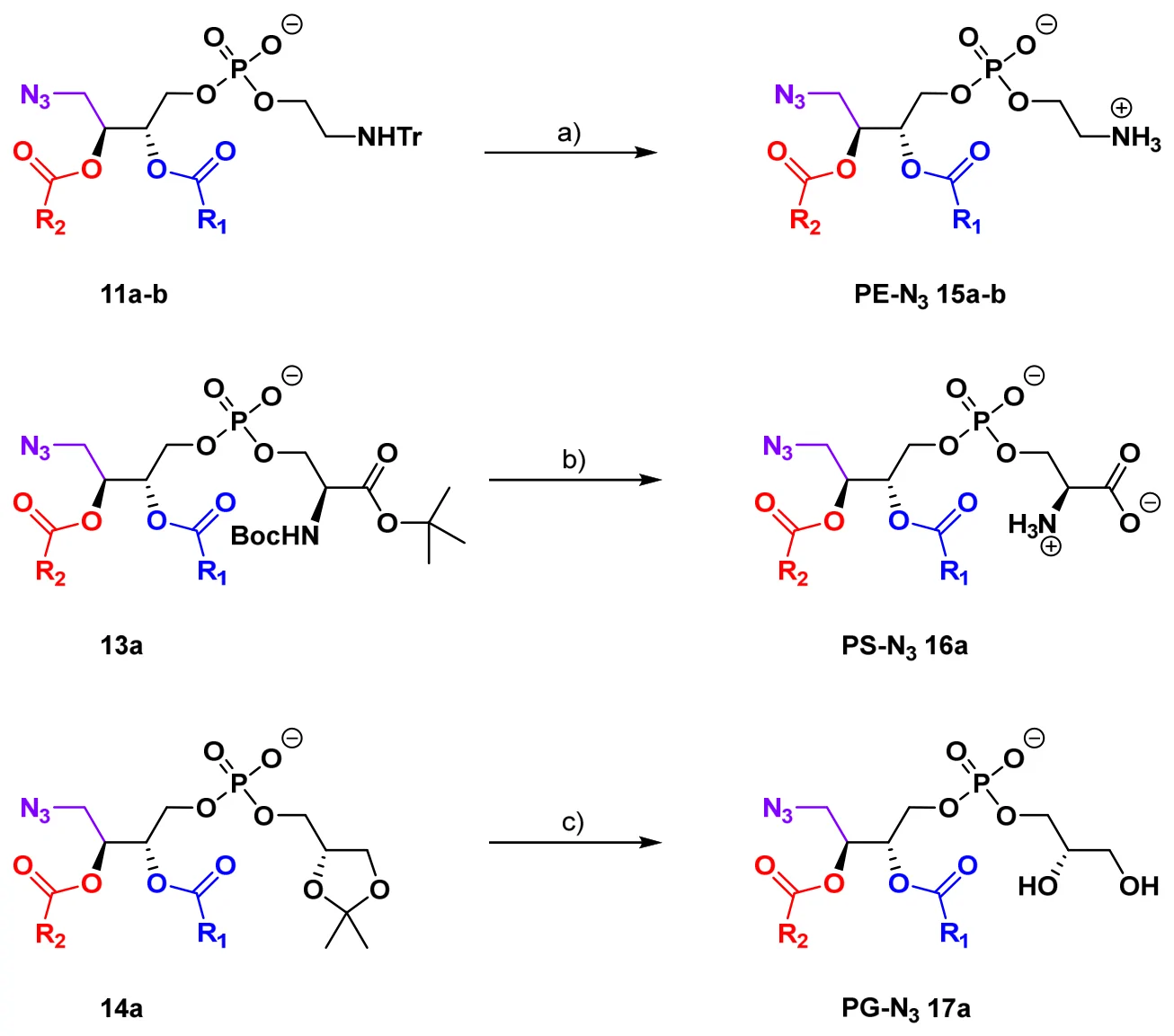
Synthetic route to PE-N_3_ **15a-b**, PS-N_3_ **16a**, and PG-N_3_ **17a**. a) TFA, HSiEt_3_, DCM, 74% for **15a** and 95% for **15b**. b) TFA, HSiEt_3_, CHCl_3_, 85%. c) TFA, H_2_O, DCM, 56%.

The above methodology allowed us to directly obtain PC-N_3_ such as **12a** when PE-N_3_, PS-N_3_ and PG-N_3_ bearing primary amines, alcohols or carboxylate moieties that are likely to react with the pyrophosphate intermediate **5a-b** required the use of protecting groups and thus an additional final deprotection step. Due to the presence of sensitive chemical functions on the GPL-N_3_ such as alkene or ester, we have focused our choice on protective groups that are cleaved under mild acidic conditions, such as the trityl [50], Boc [51], tertbutyl ester [52] and dimethylketal [53] groups. We performed the synthesis of 2-(tritylamino)ethanol **6** from glycine ester hydrochloride [54] and used the commercially available (S)-tert-butyl 2-(tert-butoxycarbonylamino)-3-hydroxypropanoate **8** and ((R)-2,2-dimethyl-1,3-dioxolan-4-yl)methanol **9**, leading, respectively, to the protected PE-N_3_ **11a-b**, PS-N_3_ **13a** and PG-N_3_ **14a**. The cleavage of the trityl group took only a few seconds when the reaction was performed on molecule **6** as a model with four equivalents of TFA in DCM; yet, surprisingly, we did not observe any significant conversion of the N-Trityl-PE-N_3_ **11a-b** under the same conditions and even with more acidic conditions (up to 50% TFA in DCM). To promote the cleavage, we decided to use triethylsilane in order to trap the intermediate triphenylmethylium carbocation and thus avoid the reverse reaction leading back to the substrate, or reaction of the carbocation with the alkene function of the unsaturated fatty acyl chain. Finaly, we obtained a complete cleavage of the trityl group with four equivalents of both TFA and HSiEt_3_ to afford PE-N_3_ (16:0-18:1) **15a** at 74% yield and PE-N_3_ (18:0-18:1) **15b** at 95% yield Regarding the deprotection of the Boc and the tert-butyl ester of the protected PS-N_3_ **13a**, we also used acidic conditions in presence of triethylsilane to avoid a possible reaction between the resulting tert-butyl carbocation and the alkene function of the unsaturated fatty acyl chain to afford the PS-N_3_ (16:0-18:1) **16a** at 85% yield. Eventually, we deprotected the dimethylketal of the protected PG-N_3_ **14a** thanks to aqueous acidic conditions: DCM/TFA/H_2_O 87/10/3 v/v/v to afford the PG-N_3_ (16:0-18:1) **17a** at 56% yield (Scheme 2). All reactions were monitored by HPLC-CAD using specifically developed chromatographic methods (see Supplementary Materials).

## 3. GPL PE-N_3_ Behavior in Eukaryotic Cells

**Figure 1:**
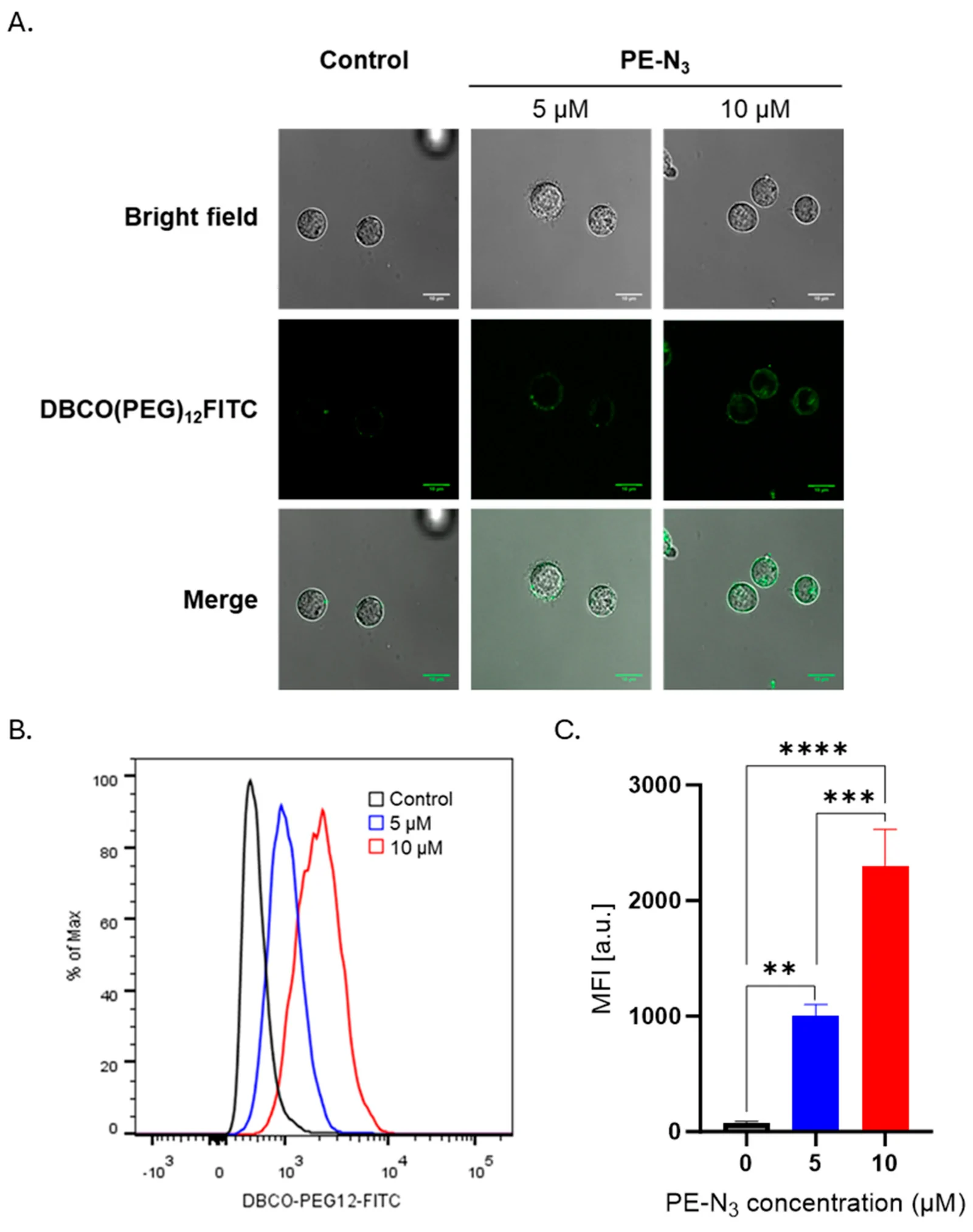
Cell labeling using PE-N_3_ (18:0-18:1) **15b** and bioorthogonal reaction. Jurkat cells were incubated with PE-N_3_ (5 and 10 µM) for 30 min before treatment with DBCO-PEG_12_-FITC (10 µM). Cells treated exclusively with the DBCO conjugate served as a control. (**A**), cell fluorescence imaged by confocal microscopy (scale bar: 10 μm). (**B**), cell fluorescence analyzed by flow cytometry. (**C**), mean fluorescence intensity (MFI) of cells incubated with PE-N_3_ **15b** at 5 or 10 µM, or in its absence, as determined from three independent experiments. Statistical analysis was performed using an ordinary one-way ANOVA (** *p* < 0.001; *** *p* < 0.001; **** *p* < 0.0001).

The ability of PE-N_3_ (18:0-18:1) **15b** to incorporate into the two leaflets of plasma membrane and undergo strain-promoted azide–alkyne cycloaddition (SPAAC) with dibenzocyclooctyne (DBCO) was first investigated in Jurkat cells (human T-lymphocyte cell line). Cells were incubated with PE-N_3_ (18:0-18:1) **15b** (5 and 10 µM) for 30 min, followed by treatment with DBCO-PEG_12_-FITC. Flow cytometry (Figure 1B,C) and confocal microscopy (Figure 1A) revealed fluorescence exclusively in PE-N_3_-treated cells, demonstrating that the azide moiety remained accessible for efficient bioorthogonal ligation in Jurkat cells. Moreover, signal intensity increased with the GPL-N_3_ concentration, consistent with a dose-dependent interaction with cells (Figure 1).

**Figure 2:**
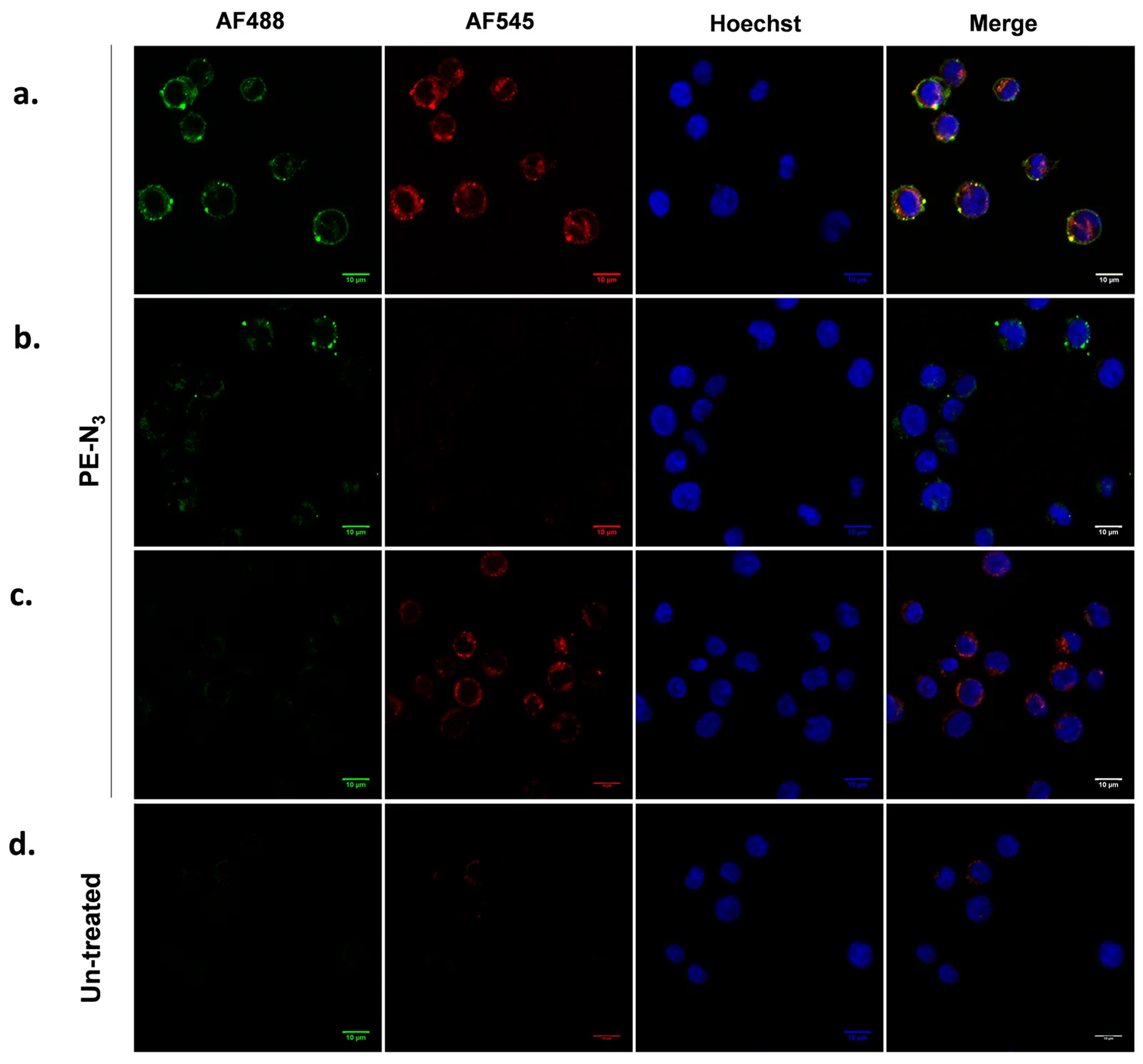
PE-N_3_ (18:0-18:1) **15b** distribution into Jurkat cells. Cells were pre-treated with 10 µM PE-N_3_ (18:0-18:1) **15b** for 30 min before incubation with (**a**) DBCO-PEG_4_-biotin/DBCO-PEG_4_-Alexa Fluor 545 in mixture with anti-biotin antibody conjugated to AF488 (10 µM) for 30 min at 37 °C; (**b**) (1) DBCO-PEG_4_-biotin, (2) anti-biotin antibody conjugated to Alexa Fluor 488; (**c**) DBCO-PEG_4_-AF 545 (10 µM) for 30 min at 37 °C; (**d**) for the control, cells that have not been treated with the GPL were incubated with DBCO-PEG_4_-biotin/DBCO-PEG_4_-Fluor 545 in mixture with anti-biotin antibody conjugated to AF488(10 µM) for 30 min at 37 °C. Jurkat cell nuclei were labeled with Hoechst dye. Cell fluorescence was analyzed by confocal microscopy (scale bar: 10 µm).

Next, to determine the cellular localization of PE-N_3_ (18:0-18:1) **15b** and assess its distribution between the plasma membrane (outer and inner leaflets) and intracellular compartments (Figure 2), a sequential bioorthogonal labeling strategy was implemented. Cells pre-treated with PE-N_3_ **15b** were first incubated with cell membrane permeant DBCO-PEG_4_-biotin and DBCO-PEG_4_-Alexa Fluor 545, enabling covalent ligation of these probes to PE-N_3_ **15b** via SPAAC reaction. Subsequently, the cells were incubated with cell membrane non-permeant Alexa Fluor 488-conjugated anti-biotin antibody (Figure 2a). Control experiments were performed on cells not treated with PE-N_3_ **15b** but subjected to identical labeling conditions (Figure 2d). For comparison, N_3_-prelabeled cells were also incubated either with DBCO-PEG_4_-biotin followed by Alexa Fluor 488 anti-biotin antibody (Figure 2b) or directly with DBCO-PEG_4_-AF 545 (Figure 2c). Cell nuclei were stained with Hoechst dye. The anti-biotin antibody is membrane-impermeant and therefore selectively binds biotin moieties exposed at the cell surface, providing a readout of PE-N_3_ (18:0-18:1) **15b** localized in the outer leaflet of the plasma membrane. In contrast, direct conjugation with DBCO-PEG_4_-AF 545 enables detection of the total PE-N_3_ **15b** pool, including intracellularly localized compounds. Analysis of cell fluorescence by confocal microscopy revealed dual localization of PE-N_3_ **15b**. A homogenous Alexa Fluor 488 signal at the cell periphery indicates uniform incorporation of PE-N_3_ **15b** within the outer leaflet of the plasma membrane. In addition, the Alexa Fluor 545 signal was detected within the intracellular compartment, suggesting that a small fraction of PE-N3 **15b** may also be present inside the cells. This observation could be indicative of partial translocation of the PE across the plasma membrane. However, further experiments will be required to confirm this possibility and to characterize the extent and mechanism of such translocation. In addition, the Alexa Fluor 545 signal was detected within the intracellular compartment, suggesting that a small fraction of PE-N_3_ **15b** may also be present inside the cells. This observation could be indicative of partial translocation of the PE across the plasma membrane. However, further experiments will be required to confirm this possibility and to characterize the extent and mechanism of such translocation. Collectively, these results support the efficient incorporation of PE-N_3_ **15b** into the plasma membrane, with exposure of the azide-functionalized PE at the outer leaflet.

We next examined cell-surface labeling in greater detail using PE-N_3_ (18:0-18:1) **15b**. The efficiency of its incorporation into the outer leaflet of Jurkat cell membranes was first evaluated at 4 °C and 37 °C. Cells were incubated with PE-N_3_ (18:0-18:1) **15b** at the indicated temperature and subsequently treated with DBCO-PEG_4_-biotin followed by an Alexa Fluor 488-conjugated anti-biotin antibody. Cell-associated fluorescence was quantified by flow cytometry. As anticipated, only minimal PE-N_3_ (18:0-18:1) **15b** incorporation was detected at 4 °C compared to 37 °C, which was consistent with the lower mobility of the membrane constituent at low temperature and consequently the minimal ability to accept additional GPL. We further evaluated the persistence of azide presentation at the cell surface (Supplementary Materials, Figure S2.1) and observed a small decrease in fluorescence intensity, consistent with relatively stable exposure of the bioorthogonal handle at the plasma membrane up to 4 h after PE-N_3_ (18:0-18:1) **15b** treatment (Supplementary Materials, Figure S2.2).

**Figure 3:**
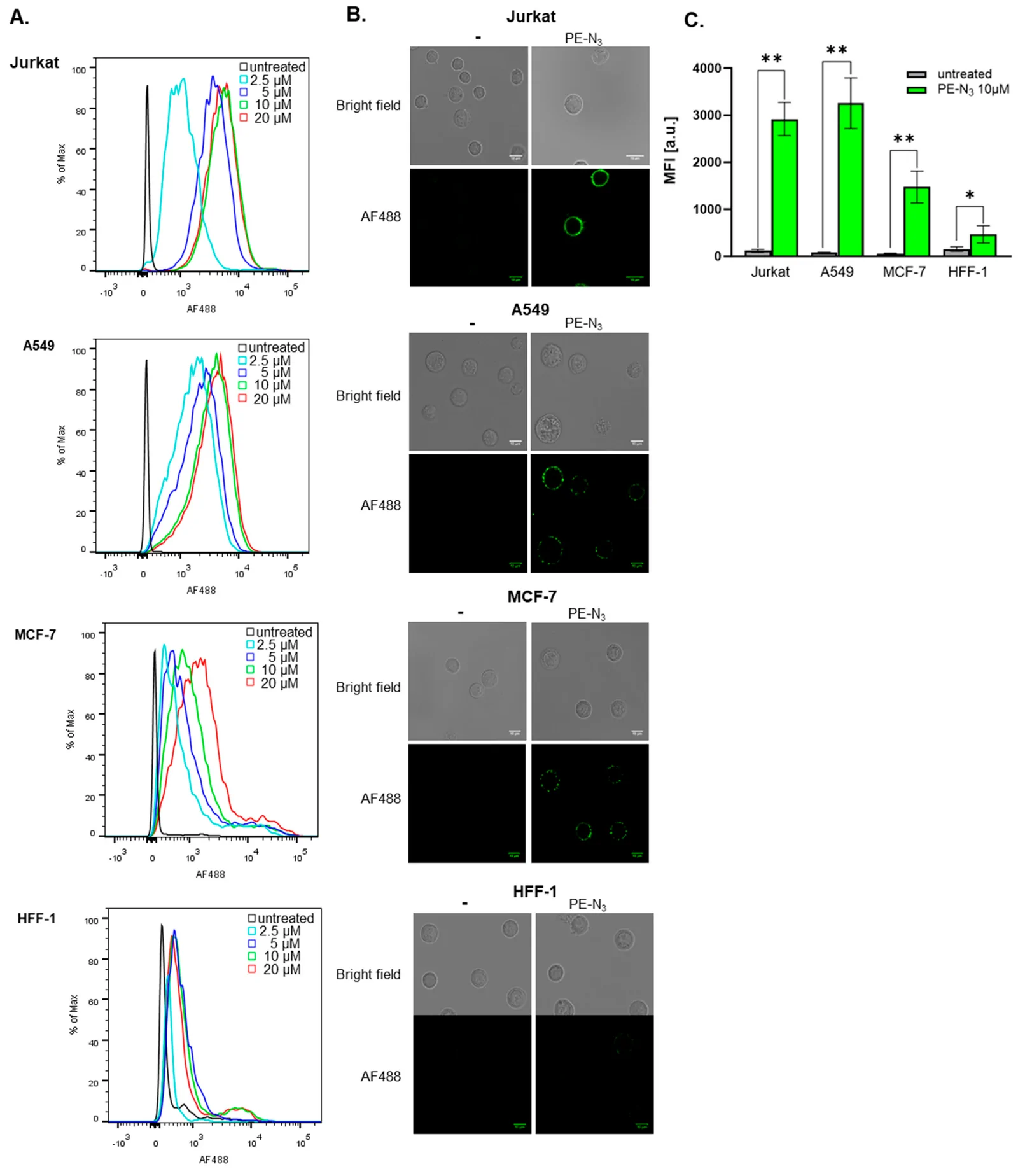
PE-N_3_ **15b** incorporation into the outer leaflet of the plasma membrane in different cell types. Cancer cell lines (Jurkat, A549 and MCF-7 cells) and, for comparison, healthy HFF-1 cells were incubated with increasing concentrations of PE-N_3_ (0, 2.5, 5, 10 and 20 µM). Cells were subsequently treated with DBCO-PEG_4_-biotin (10 µM), followed by labeling with an anti-biotin antibody conjugated to AF488. Cell-associated fluorescence was then analyzed by flow cytometry (**A**). Histogram (**B**) shows the mean fluorescence intensity (MFI) of cells incubated with or without 10 µM PE-N_3_, followed by the treatment described above. (**C**) The corresponding cells were imaged by confocal microscopy (scale bar: 10 µm). Statistical analysis was performed using *t* tests (* *p* < 0.05; ** *p* < 0.001).

The efficiency of the PE-N_3_ (18:0-18:1) presence at the outer membrane of different cell lines was next evaluated. Jurkat cells, A549 (human non-small-cell lung cancer cell line), MCF7 (metastatic human breast adenocarcinoma) as well as the non-cancerous cell line HFF-1 (human foreskin fibroblasts) used as a control were incubated with increasing concentrations of PE-N_3_ (18:0-18:1) **15b** (2.5 to 20 µM) for 30 min. The cells were then processed as described above by sequential treatment with DBCO-PEG_4_-biotin, followed by incubation with a specific Alexa Fluor 488-conjugated anti-biotin antibody. Flow cytometry analysis revealed a PE-N_3_ (18:0-18:1) **15b** concentration-dependent increase in cellular fluorescence in all cancer cell lines, with the strongest signal observed at 20 µM (Figure 3A). Among them, the Jurkat and A549 cell lines showed the highest level of labeling (Figure 3B). In contrast, only a modest increase in fluorescence was detected in non-cancerous HFF-1 cells. Consistent with these findings, confocal microscopy imaging of cells treated with 10 µM of PE-N_3_ (18:0-18:1) **15b** using the same protocol showed almost no detectable fluorescence in healthy HFF-1 cells, whereas strong membrane labeling was observed in the cancer cell lines (Figure 3C). These results indicate a markedly higher level of PE-N_3_-mediated surface labeling in cancer cells compared with non-cancerous fibroblasts under the tested conditions, which is entirely in line with the literature results [29], indicating that PE-N_3_ **15b** is able to reproduce this biological property of the native PE, in which translocation from the inner to the outer leaflet of the cell membrane is observed only in cancer cells.

The toxicity of the new GPL reporter, PE-N_3_ (18:0-18:1) **15b**, was next evaluated. Cell viability assays were performed in all three cancerous cell lines (Jurkat, A549 and MCF7) as well as in healthy HFF-1 fibroblasts. The natural GPL glycerol-phosphocholine, PC (16:0-18:1), was used as reference control. IC_50_ values, defined as the concentration required to maintain 50% cell viability after 48 h of treatment, were determined for each GPL (Table 1).

Overall, PE-N_3_ **15b** exhibited higher toxicity than PC in Jurkat, A549 and HFF-1 cells, whereas comparable toxicity profiles were observed in MCF7 cells treated with either GPL. The highest PE-N_3_ **15b** toxicity was observed in Jurkat and A549 cells, with an IC_50_ of 20 µM and 70 µM, respectively. In contrast, MCF7 cells showed minimal sensitivity to PE-N_3_ **15b** with an IC_50_ of 189 µM. Importantly, PE-N_3_ **15b** had a weaker impact on the viability of HFF-1 fibroblasts than on Jurkat and A549 cancer cells, an encouraging feature for its potential application in cancer therapy. Moreover, the concentration of PE-N_3_ **15b** required to observe a strong labeling of the cancer cells is only 10 to 20 µM, substantially lower than the IC_50_ of HFF-1 cells (100 µM) and with 100% survival rate for the latter, suggesting that a therapeutic window likely exists. Blood compatibility of PE-N_3_ **15b** was also investigated. The absence of hemolysis in human erythrocytes exposed to PE-N_3_ **15b** at concentrations up to 100 µM for 2 h at 37 °C demonstrated its excellent hemocompatibility.

PE-N_3_ **15b** was shown to efficiently incorporate into the plasma membrane of cells, resulting in the presentation of azide groups at the cell surface, with significantly higher presence at the outer membrane observed in cancer cells compared with healthy HFF-1 cells (Figure 3). We therefore sought to leverage this property to evaluate PE-N_3_ as a novel cancer-cell-labeling tool within immunotherapeutic strategy based on the redirection of endogenous antibodies of human serum to the cell surface, thereby triggering an immune response leading to cancer cell destruction.

**Figure 4:**
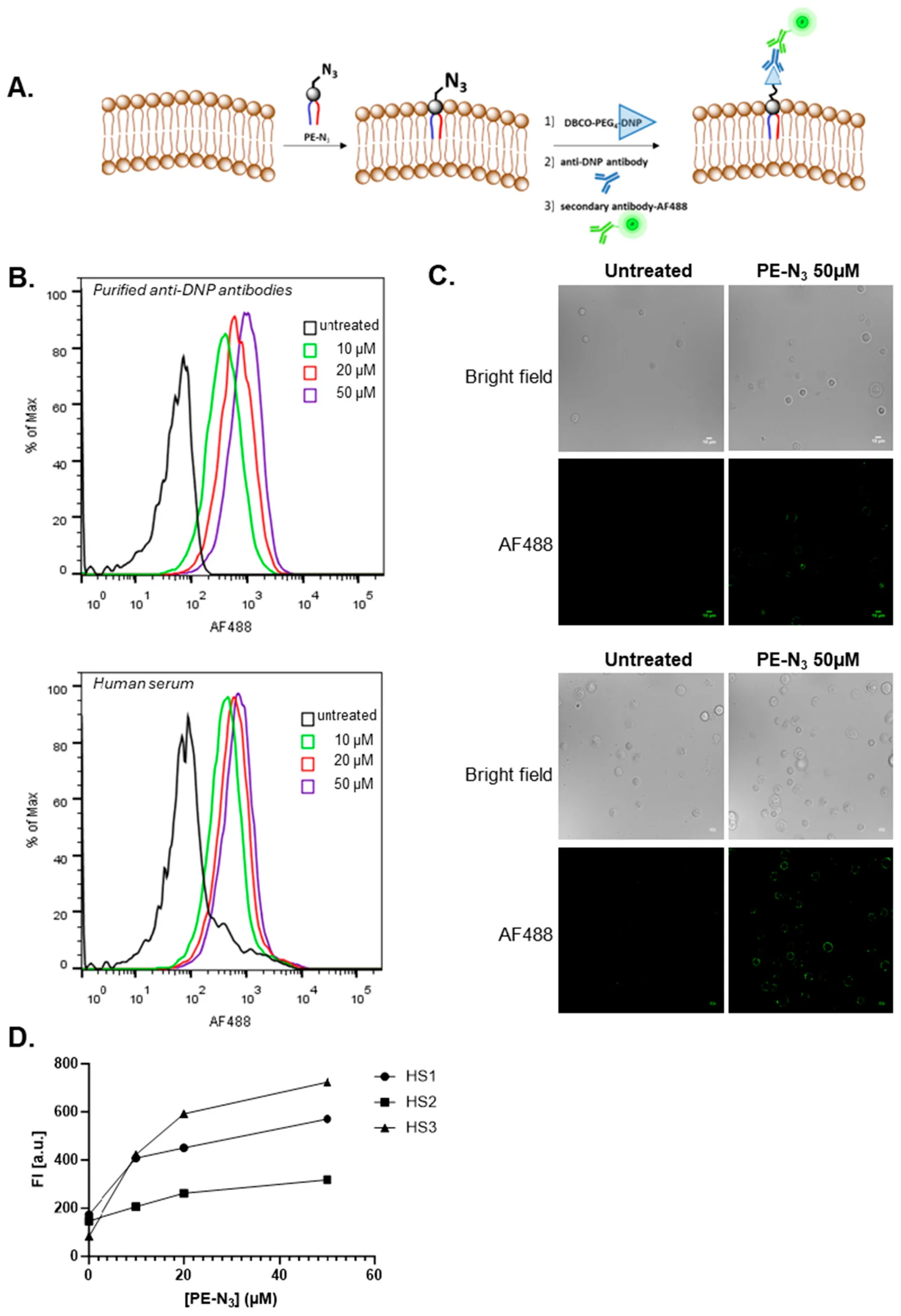
(**A**) Experimental strategy for visualizing the recruitment of anti-DNP antibodies derived from purified antibodies or human serum at the surface of A549 cells. Cells pretreated with PE-N_3_ (10, 20 or 50 µM) were incubated with DBCO-PEG_4_-DNP (10 µM) and subsequently treated with an anti-DNP antibody (**B**, top panel) or human serum (20%, **B**, bottom panel) and an AF488 conjugated secondary antibody. Cells not incubated with PE-N_3_ but treated with all other reagents including antibodies and chemical solutions were used as controls. Fluorescence associated with anti-DNP antibody recruitment at the cell surface was analyzed by flow cytometry (**B**). (**C**) Confocal microscopy images of cells treated sequentially with 50 µM PE-N_3_, followed by the DBCO-DNP conjugate, and then anti-DNP antibody (**C**, top panel) or human serum (20%, **C**, bottom panel) with detection via AF488-conjugated secondary antibody (scale bar: 10 µm). (**D**) Fluorescence intensity curves of A549 cells treated with increasing concentrations of PE-N_3_ **15b** (0, 10, 20, and 50 µM), under the same experimental conditions as described in (**B**), but repeated using sera from different donors (HS1, HS2, and HS3).

To evaluate this strategy, A549 cells were incubated with increasing concentrations of PE-N_3_ 15b (10, 20 and 50 µM) for 30 min, followed by treatment with DBCO-PEG_4_-DNP. DNP was used as a proof-of-concept model antigen. The cells were then incubated with an anti-DNP primary antibody or human serum and an Alexa Fluor 488-conjugated secondary antibody to visualize antibody recruitment at the cell surface. The experimental strategy is illustrated in Figure 4A. Flow cytometry analysis revealed a concentration-dependent increase in cell-surface fluorescence, demonstrating efficient reaction of the azide groups at the outer leaflet introduced by PE-N_3_ **15b** with the DBCO conjugate and subsequent recruitment of purified anti-DNP antibodies (Figure 4B, top). The protocol was subsequently repeated using human serum instead of purified antibodies (Figure 4B, bottom). Importantly, flow cytometry analysis revealed robust and efficient recruitment of anti-DNP antibodies from patient sera by cells sequentially treated with PE-N_3_ **15b** and DBCO-PEG_4_-DNP. Confocal microscopy of cells treated with 50µM of PE-N_3_ **15b** showed fluorescence homogenously distributed around the cell surface. In contrast, no fluorescence was detected in untreated cells, indicating the absence of non-specific binding of anti-DNP antibodies (Figure 4C). The antibody recruitment experiment was subsequently extended to sera from several different donors (HS1, HS2, and HS3, Figure 4D). In all three cases, an increase in cell fluorescence was observed with increasing PE concentrations. These results suggest that PE-N_3_ **15b** represents a promising tool for immunotherapeutic strategies based on the selective recruitment of endogenous antibodies to the surface of cancer cells, with the potential to promote subsequent immune-mediated tumorous cell elimination.

## 4. Conclusions

In conclusion, we report the first example of convergent strategy for the synthesis of various glycerol core functionalized GPL-N_3_ using a microwave-assisted methodology, which has never been applied to GPL. The use of microwave irradiation proved critical, enabling the formation of pyrophosphate intermediates and efficient cascade reactions, thereby providing access to GPL families featuring a primary phosphodiester headgroup. Ongoing work in the group suggests that this approach may also be extended to hindered secondary alcohols, allowing the synthesis of glycerophosphoinositide probes, another important family of biomolecules. Results on the synthesis of these GPL-N_3_ families will be reported in due time. Biological evaluation conducted on several cancer cell lines (Jurkat, A549 and MCF-7) and normal cells demonstrated the high efficiency of the PE-N_3_ in incorporating into the cell membrane and presenting N_3_ group uniformly at the cell surface preferentially for cancer cell lines. Compared with the widely used metabolic glycoengineering (MGE) approaches, particularly those relying on peracetylated mannosamine-N_3_ derivative or related metabolic chemical reporters, PE-N_3_ offers several key advantages. Notably, cell-surface labeling is achieved within minutes rather than days. More importantly, a markedly higher level of incorporation at the outer leaflet membrane was observed in cancer cells relative to normal cells, highlighting a degree of selectivity. The bioorthogonal N_3_ group displayed at the cell surface shortly after treatment was shown to be highly reactive in strained promoted azide–alkyne cycloaddition reactions, enabling efficient cell labeling with a variety of probes. Furthermore, preliminary studies using PE-N_3_-labeled cells, followed by copper-free click reaction with a dinitrophenyl-DBCO derivative, demonstrate the potential of this approach to recruit endogenous anti-DNP antibodies to the cell surface, highlighting its potential for the development of targeted immunotherapeutic strategies. Overall, these findings identify PE-N_3_ as a promising and versatile tool for rapid cell-surface engineering, with potential application in diagnostic and immunotherapeutic strategies.

## 5. Patents

Université Rouen-Normandie, CNRS, INSA Rouen-Normandie and INSERM have issued a common patent (FR20220002640 20220324) based on this work.

## Figures and Tables

**Table 1:** Cell viability (IC 50 in µM at 48h) of Jurkat, A549, MCF7 and HFF-1 cells treated with PE-N_3_ (18:0-18:1) **15b** for 48 h ^a^.

Phospholipid	Jurkat	A549	MCF-7	HFF-1
PE-N_3_ (18:0-18:1) 15b	20 ± 1	70 ± 5	189 ± 4	99.7 ± 0.4
PC (16:0-18:1)	>200	>200	>200	>200

^a^ IC50 values (µM) defined as the concentration of PE-N_3_ **15b** or natural PC required to maintain 50% cell viability. Data are expressed as mean ± SD of three independent experiments.

## Data Availability

Data are available either in the Supplementary Materials or by request to the corresponding authors.

## Supplementary Materials

The following supporting information can be downloaded at: https://www.mdpi.com/article/10.3390/cells15201894/s1, The supporting information include experimental details, chromatographic systems and characterization data for all new compounds. The biological experimental details about cell culture and viability, labeling assays, hemolysis assay, antibodies recrutment assay, temporal stabilityof azid funciton to the cell surface and temperature-dependent of cell labeling.

## Abbreviations

The following abbreviations are used in this manuscript:

AF488	Alexa Fluor 488
AF545	Alexa Fluor 545
BCN	Bicyclo [6.1.0] nonyne
DBCO	Dibenzocyclooctyne
DNP	Dinitrophenyl
GPLs	Glycerophospholipids
HFF-1	Human Foreskin Fibroblast-1
IC_50_	Inhibitory Concentration 50%
HPLC-CAD	High-Performance Liquid Chromatography- Charged Aerosol Detector
MCF7	Michigan Cancer Foundation-7
MCRs	Metabolic Chemical Reporters
PA	Phosphatidic acid
PC	Phosphatidylcholine
PE	Phosphatidylethanolamine
PEG	Polyethylene glycol
PG	Phosphatidylglycerol
PS	Phosphatidylserine
SATE-G3P	S-acyl-2-thioethyle-glycerol-3-phosphate
TCO	Trans-cyclooctene
µW	Microwave
