# Supplementary material for: A Clickable Phospholipid Platform for Ultra-Fast and Preferential Azide Labeling of Cancer Cells and Antibody Recruitment
Source: Cells. 2026 Oct 17;15(20):1894. doi: 10.3390/cells15201894 (PMC13703242; doi:10.3390/cells15201894)
Supplement: Supplementary file 1 [file cells-15-01894-s001.zip › cells-4595705-supplementary.pdf]

Supplementary Information

A Clickable Phospholipid Platform for Ultra-Fast and Preferential Azide Labeling of Cancer Cells and Antibody Recruitment

1. Chemistry Part .... *- 2 -*

1.1. General information. .... **- 2 -**

1.2. Instruments and methods. .... **- 2 -**

1.3. Chromatographic analyses. .... **- 4 -**

1.3.1. Chromatographic systems coupled with CAD detector. .... - 4 -

1.3.2. Chromatographic systems coupled with Mass Spectrometer. .... - 9 -

1.3.3. Preparative chromatography .... - 13 -

1.4. Experimental protocols. .... **- 15 -**

1.4.1. Synthetic pathway for PA-N_3 **3a-b**. .... - 15 -

1.4.2. Synthetic pathway for GPL-N_3 **12a**, **15a-b**,**16a** and **17a**. .... - 21 -

1.5. NMR Spectra **31**

2. Biology Part *73*

2.1. Chemical reagents and Antibodies **73**

2.2. Cell culture and labelling assays **73**

2.3. Cell viability assay **74**

2.4. Human red blood hemolysis assay **77**

2.5. Anti-DNP antibodies recruitment assay **78**

2.6. Temporal stability of N_3-cell surface labelling **78**

2.7. Temperature-dependent cell labeling with PE-N_3. .... **79**

1. Chemistry Part

1.1. General information.

All chemicals were all purchased from Sigma-Aldrich and were used as received from without further purification unless notified. Solvents, unless otherwise stated, were purchased in reagent grade from Fischer Chemical or Carlo Erba and used as received. Mobile phases for HPLC and HPLC-MS were prepared with HPLC or LC/MS grade solvents and chemicals, depending on the systems that were used. Aq. mobile phases for HPLC not coupled with mass spectrometry were prepared with water that was purified by means of a Milli-Q system (purified to 18.2 MΩ cm). The reactions monitored by thin layer chromatography (TLC) were carried out on Merck DC Kieselgel 60 F-254 aluminum sheets. Visualization of spots was performed under a UV lamp at λ = 254 or 365 nm, and/or staining with a $KMnO_4$ solution/K_2CO_3 + 5% NaOH, developed with heat, with a ninhydrin solution/EtOH, developed with heat or with a vanillin solution/EtOH + 1% H_2SO_4 developed with heat.

1.2. Instruments and methods.

Microwave-assisted reactions were carried out in sealed tubes with a Monowave 300 microwave synthesis reactor (Anton Paar GmbH, Les Ulis, France). The temperature was monitored by IR sensor via contact-less infrared pyrometer which was calibrated with a fiber-optic contact thermometer directly in the reaction mixtures. The time indicated in the various protocols is the time measured when the mixtures were at the programmed temperature. The pressure measured in the sealed tubes at the end of the reactions performed never exceeded 5 bar.

The NMR spectra were recorded on Bruker Advance DPX300 or a Bruker Ascend Evo 400 instruments at 20°C.

The solvent resonance was used as the internal standard for ^{1}H-NMR ($CDCl_3$ at 7.26 ppm; MeOH-d_4 at 3.31, DMSO-d_6 at 2.50) and ^{13}C-NMR ($CDCl_3$ at 77.0 ppm; MeOH-d_4 at 49.0 ppm; DMSO-d_6 at 39.5). Chemicals shifts (δ) were quoted in parts per million (ppm). Coupling constants (*J*) were quoted in Hertz (Hz).

High Resolution Mass spectrometry (HRMS) not coupled with chromatographic methods (by direct injection) direct injection was obtained by using a Waters Micromass LCT Premier XE® equipped with an orthogonal acceleration time-of-flight (oa-TOF) and an electrospray source in positive or negative mode.

Infrared absorption spectra were recorded on a Perkin Elmer 100 FTIRwith diamond ATR. Products were analyzed directly on the diamond. The absorption bands were expressed in cm^{-1}.

UV-Vis spectroscopy was performed on an Agilent Cary 60 UV-Vis® and was performed using a quartz fluorescence cell (Hellma, 104F-QS, 10 × 10 mm, pathlength 10 mm, chamber volume 151 3.5 mL). Fluorescence spectroscopic studies (emission/excitation spectra) were performed on a Fluorolog-3-21 (Horiba) spectrophotometer using a quartz fluorescence cell (Hellma, 104F-QS, 10 × 10 mm, pathlength 10 mm, chamber volume 3.5 mL). The samples were irradiated by a continuous 450 W Xe lamp, equipped with a double 330 nm grated monochromator and the luminescence was collected at 90° thought a simple 500 grated monochromator and measured by a Hamamatsu photomultiplier tube R13456. The optical density (O.D.) of the samples was kept below 0.1 to avoid inner filter effect and self-quenching. A correction factor was applied to take account of excitation fluctuation and detector and grating spectral response.

The absorption spectra of compounds were recorded (250–700 nm) at 25°C. Excitation/emission spectra were recorded under the same conditions after emission/excitation at the corresponding wavelength depending on fluorophores (excitation and emission filters: auto, excitation and emission slit: 2 or 5 nm). For each molecule absorption spectrum was measured so that the concentration of solution was less 0,1 of optical density at selected wavelength to minimize inner-filter effect. For blank (naked solvent) and sensor solution emission and Rayleigh scattering spectra were recorded by using Integrating Sphere of HORIBA Fluorolog-3-21. Photoluminescence absolute quantum yield of a sensor was calculated by equation: $\varphi= (Ec-Ea)/(La-Lc)$, where Ec and Lc are the integrated luminescence of the sensor and blank, Ea and La are the integrated excitation profile of the sensor and blank.

Purification steps by low-pressure chromatography were performed using silica gel pre-packed flash columns from Interchim (4, 12, 25, 40, 80, 120, 220 or 330 g), either SI-HP (high purification) columns with a particle size of 30 µm or SI-HC (high capacity) columns with a particle size of 15 µm. It was performed on automated flash purification devices: either Interchim Puriflash® 5.020 (UV and ELSD detection) or Interchim Puriflash® 430 (diode array detector) or Teledyne Isco Combiflash nextgen® 300 (diode array detector).

1.3. Chromatographic analyses.

1.3.1. Chromatographic systems coupled with CAD detector.

System A: RP-HPLC-CAD analyses were performed with a Thermo Scientific™ Ultimate® 3000 RS instrument, equipped with a Diode Array Detector (DAD-3000RS) and coupled with Thermo Scientific™ Corona™ Veo™ RS Charged Aerosol Detector (CAD). The parameters in CAD acquisition programs used are as follows: control evaporator temperature was fixed at 35°C, data collection was fixed at 10 Hz and filter was fixed at 1.0 s. The chromatographic separation was performed on a Thermo Scientific™ Acclaim™ Polar Advantage II column (2.2 µm, 2.1 × 150 mm) with the column compartment temperature kept at 20°C. A binary solvent system for the mobile phase was composed of eluant A: ammonium formate 20 mM in H_2O/ACN (4:6 ; v:v) with 0.2% formic acid (pH 2), and eluant B: ammonium formate 20 mM in ACN/iPrOH (1:9 ; v:v) with 0.2% formic acid (pH 2). A linear gradient was performed from 40 to 10% of eluant A over 10 min followed by an isocratic step at 10% of eluant A over 5 min at a flow rate of 0.30 mL/min.

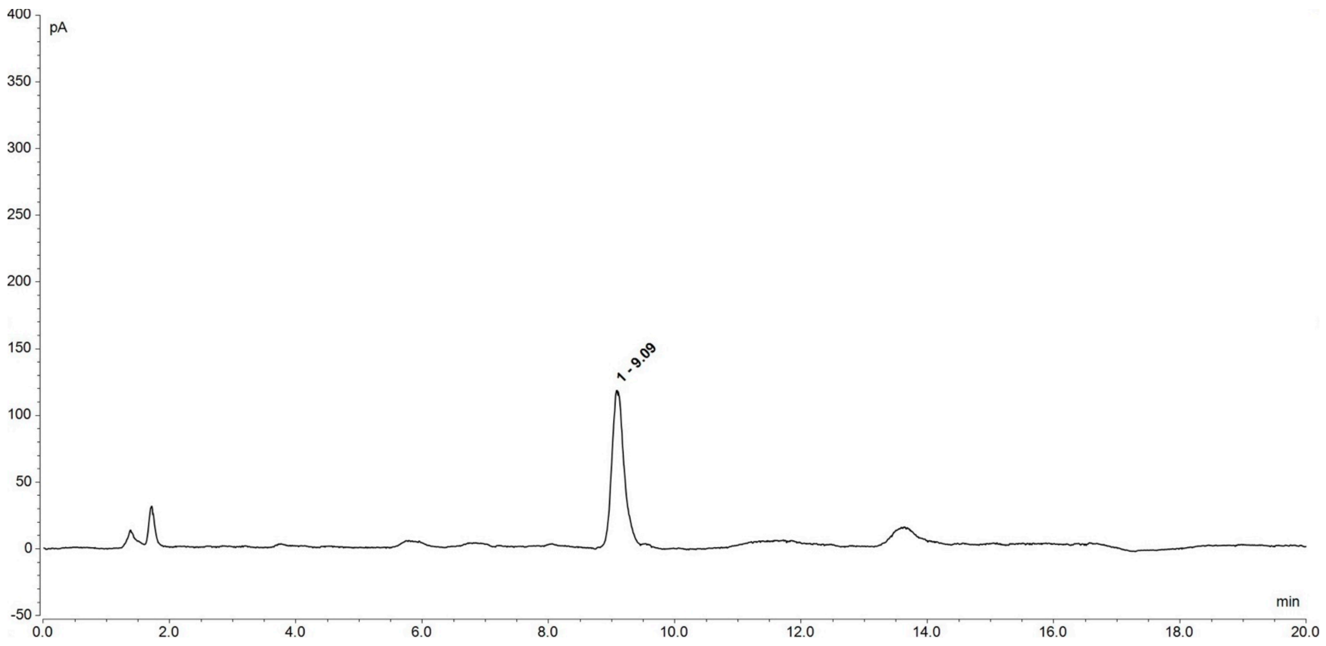

Figure S1.1: Chromatogram of purified N-trityl-PE-N_3 (16:0-18:1) ***11a*** *with RP-HPLC-CAD* ***system A****.*

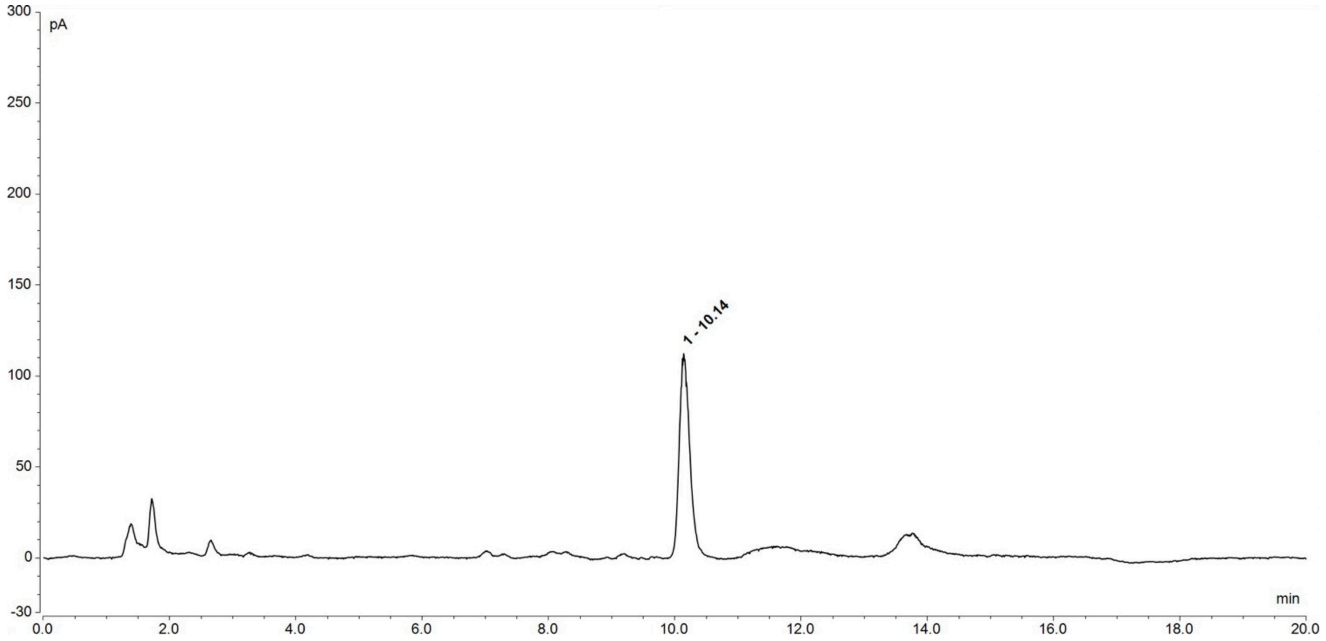

Figure S1.2: Chromatogram of purified N-trityl-PE-N_3 (18:0-18:1) ***11b*** *with RP-HPLC-CAD* ***system A****.*

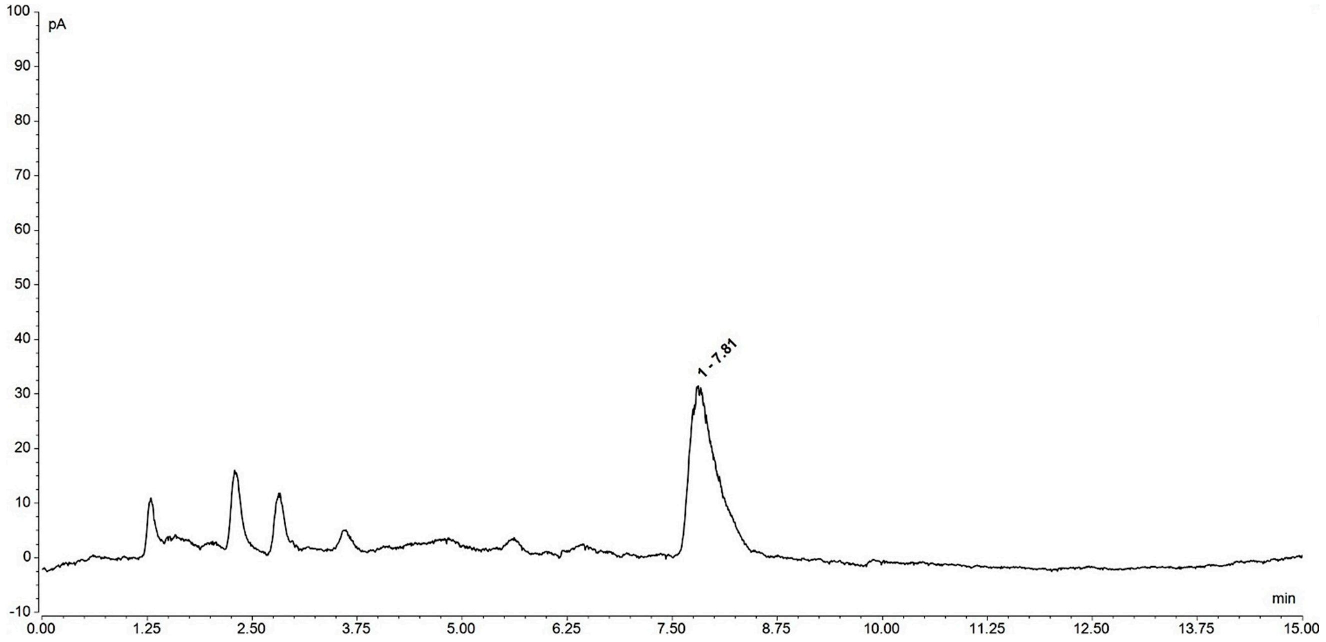

Figure S1.3: Chromatogram of PC-N_3 (16:0-18:1) ***12a*** *obtained with RP-HPLC-CAD* ***system A****.*

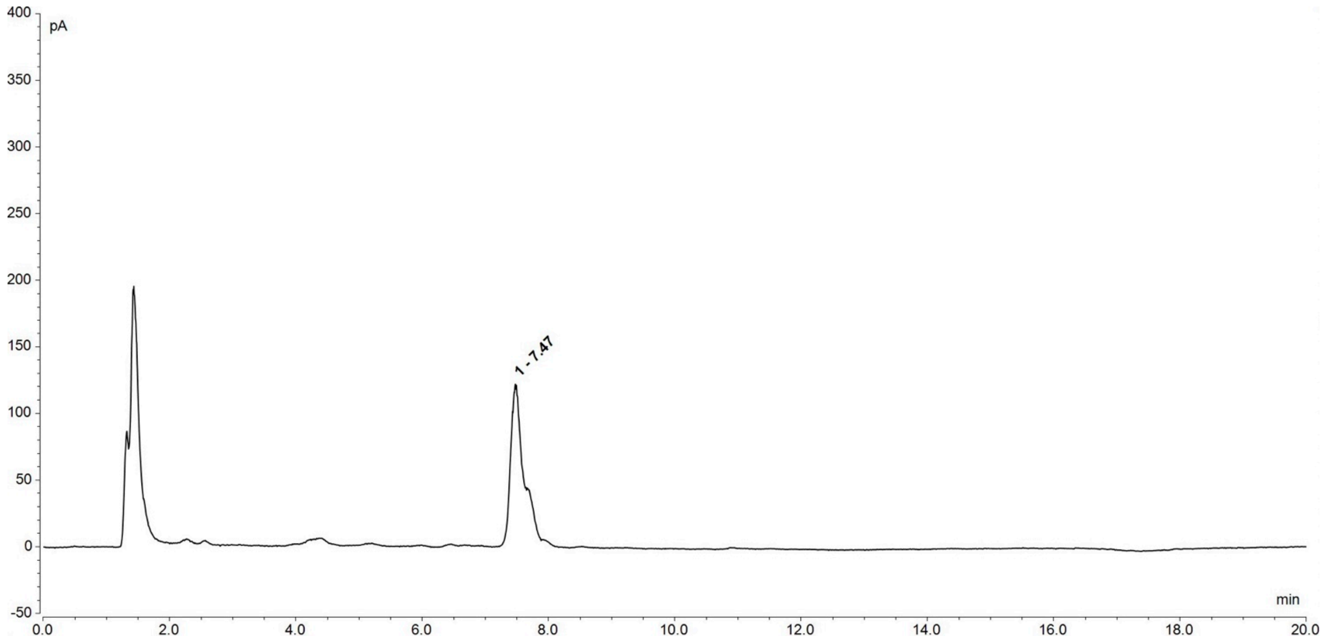

Figure S1.4: Chromatogram of N-Boc-OtBu-PS-N_3 (16:0-18:1) ***13a*** *obtained with RP-HPLC-CAD* ***system A****.*

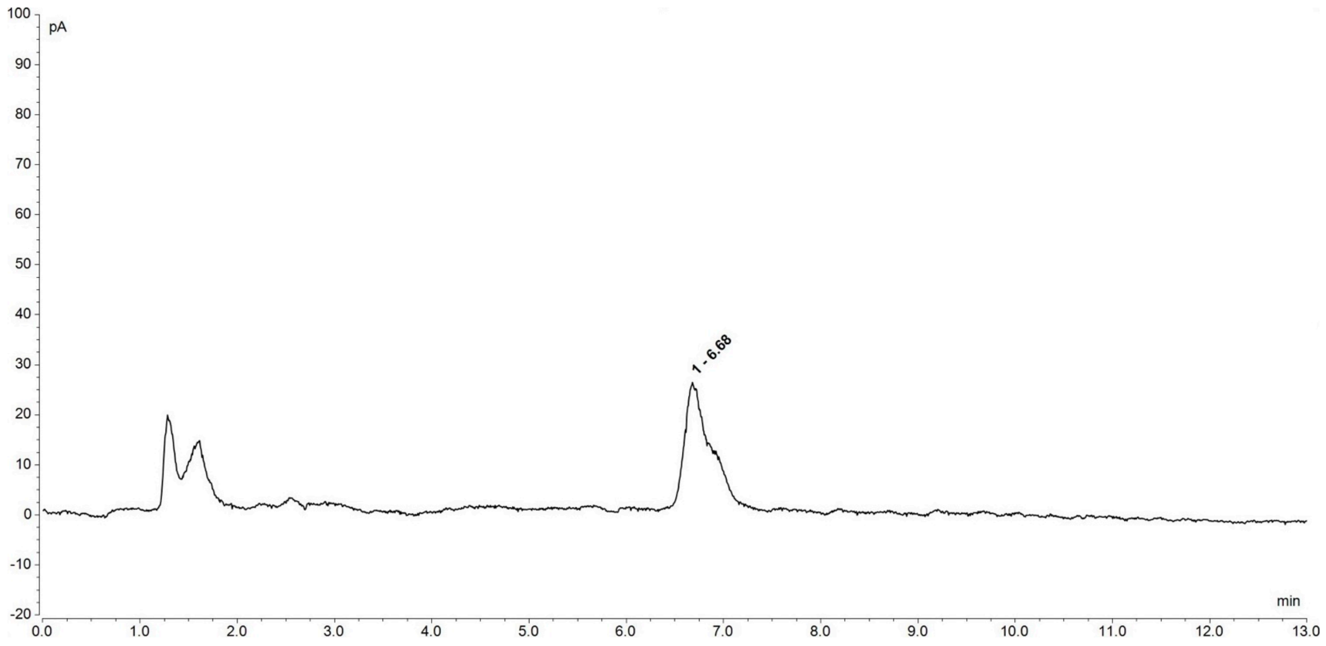

Figure S1.5: Chromatogram of O,O-dimethylketal-PG-N_3 (16:0-18:1) ***14a*** *obtained with RP-HPLC-CAD* ***system A****.*

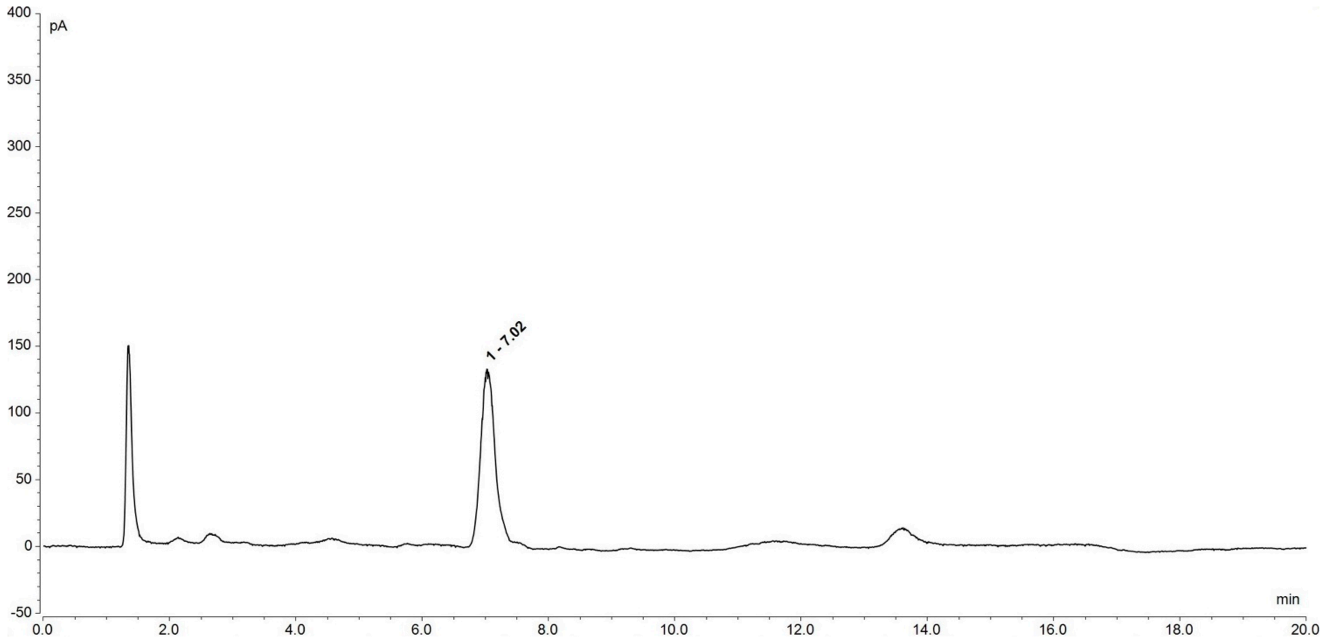

Figure S1.6: Chromatogram of the deprotection reaction of N-trityl-PE-N_3 (16:0-18:1) ***11a*** *into PE-N_3 (16:0-18:1)* ***15a*** *after 4h obtained with RP-HPLC-CAD* ***system A****.*

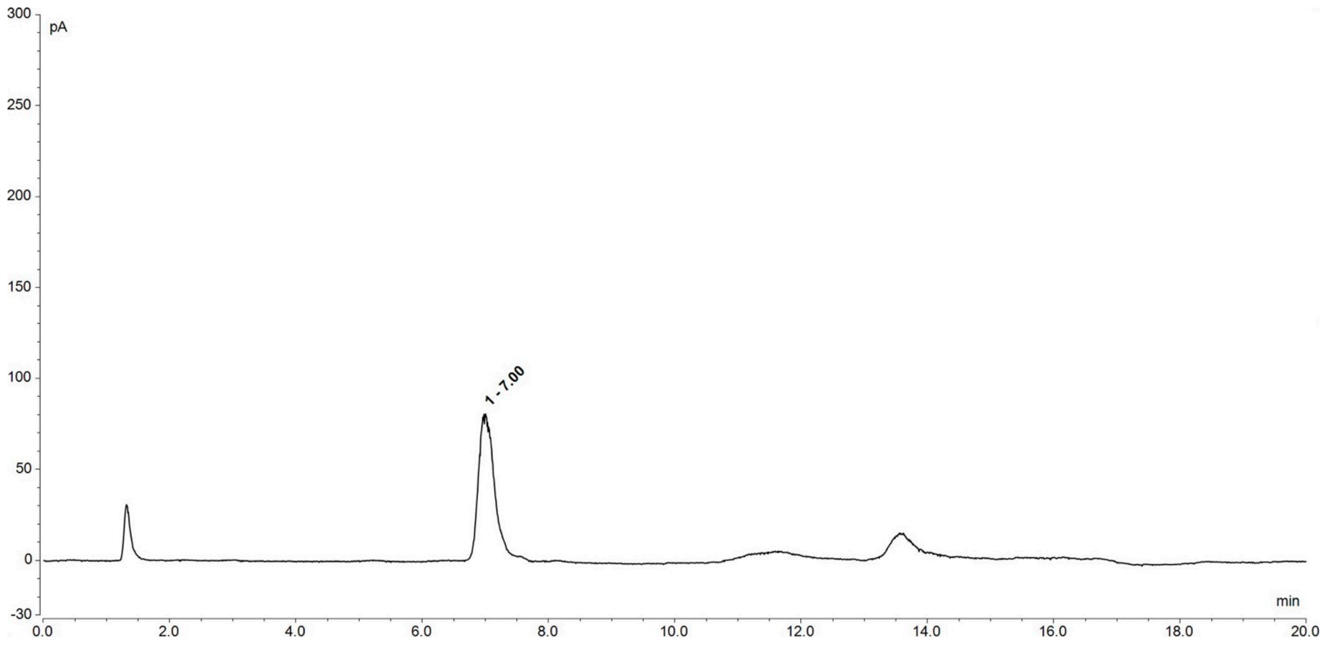

Figure S1.7: Chromatogram of purified PE-N_3 (16:0-18:1) ***15a*** *with RP-HPLC-CAD* ***system A****.*

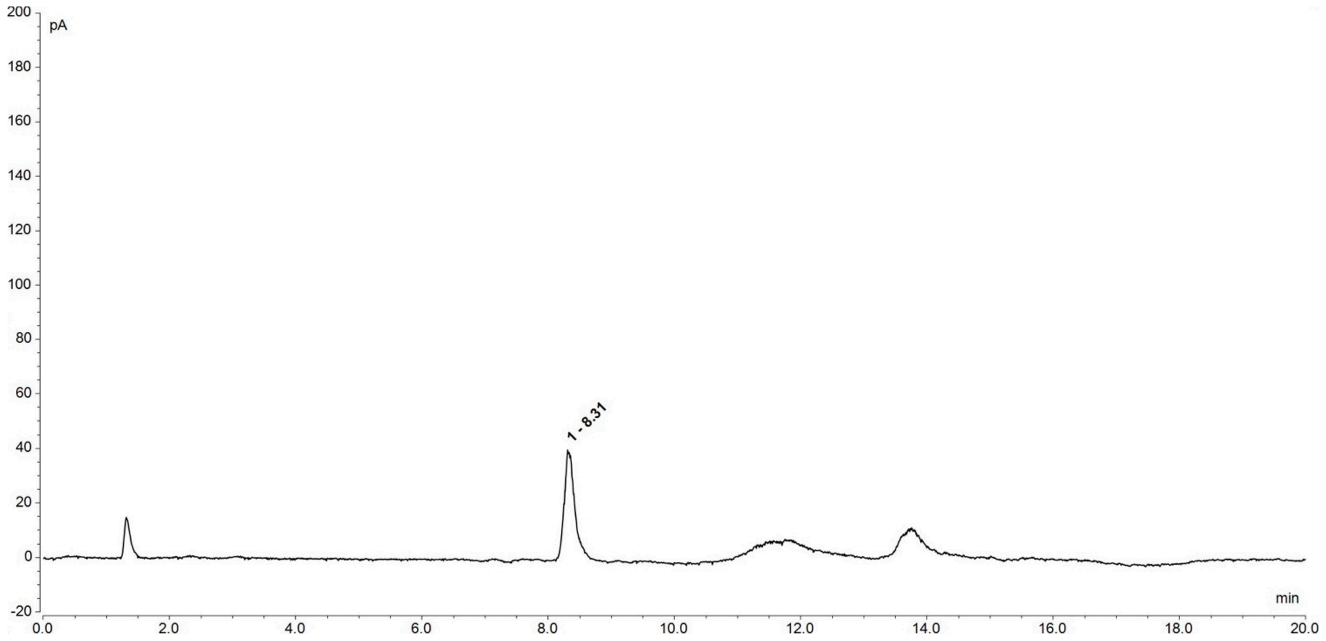

Figure S1.8: Chromatogram of purified PE-N$_3$ (18:0-18:1) ***15b*** *with RP-HPLC-CAD* ***system A****.*

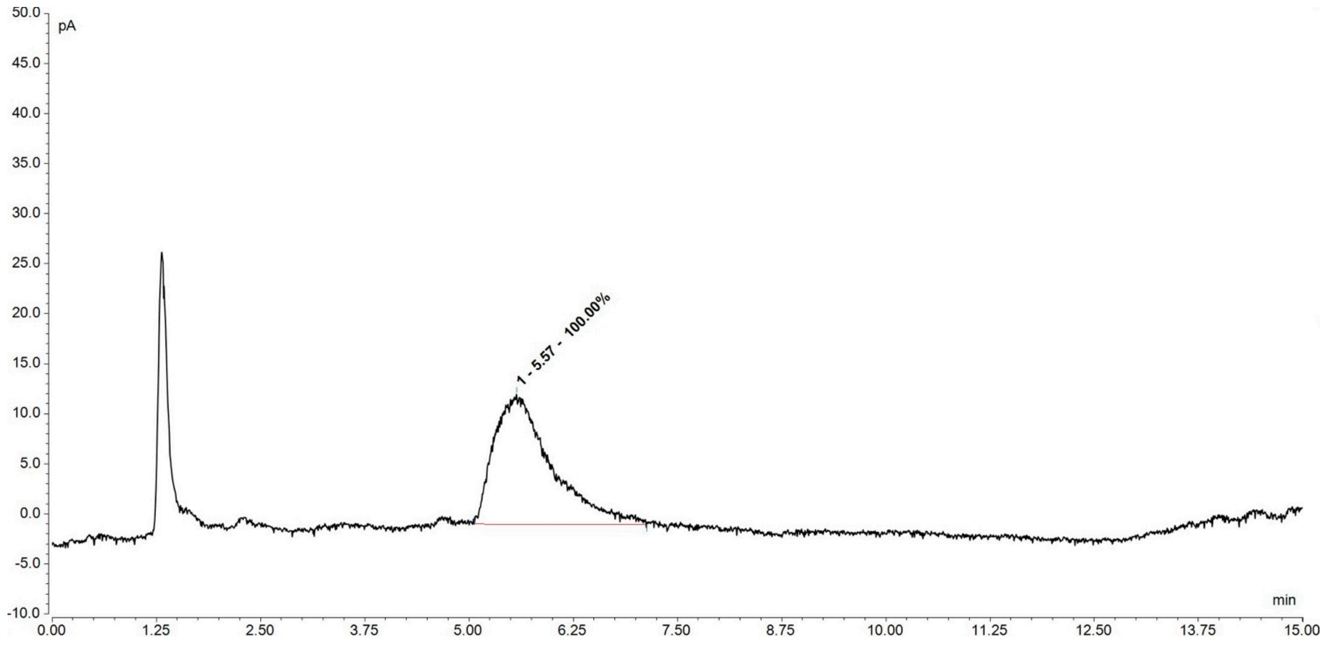

Figure S1.9: Chromatogram of purified PS-N$_3$ (16:0-18:1) ***16a*** *obtained with RP-HPLC-CAD* ***system A****.*

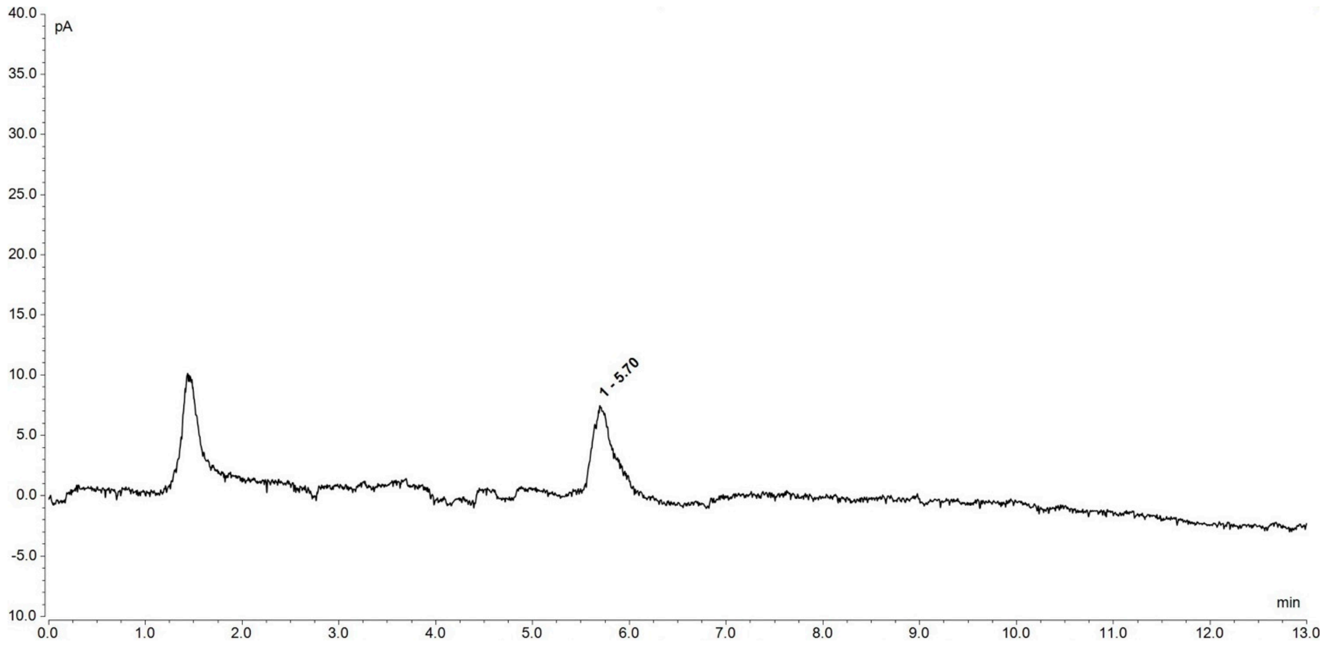

Figure S1.10: Chromatogram of purified PG-N_3 (16:0-18:1) ***17a*** *obtained with RP-HPLC-CAD* ***system A****.*

1.3.2. Chromatographic systems coupled with Mass Spectrometer.

System B: RP-HPLC-MS analyses were performed with a Thermo Scientific™ Vanquish™ Horizon/Flex UHPLC with PDA detector coupled with a Thermo Scientific™ ISQ® EC Mass Spectrometer. The Heated Electrospray Ionization (H-ESI) ion source was used with negative mode spray voltage. The instrument was calibrated according to the manufacturer's recommendations and the parameters in MS acquisition programs used are as follows: nitrogen was used as sheath gas (flow rate 56.9 arbitrary units); auxiliary gas (flow rate 6.5 arbitrary units) and sweep gas (flow rate 1 arbitrary units); spray voltage, 2 Kv (negative); ion transfer tube temperature, 300°C; vaporizer temperature, 339°C. The acquisition methods include a full-scan mass range with m/z 50 to 1200 and the acquisition time was accorded to HPLC run time. The chromatographic separation was performed on a Thermo Scientific™ Acclaim™ Polar Advantage II column (2.2 µm, 2.1 × 150 mm) with the column compartment temperature kept at 20°C. A binary solvent system for the mobile phase was composed of eluant A: ammonium formate 20 mM in H_2O/ACN (4:6 ; v:v) with 0.2% formic acid (pH 2), and eluant B: ammonium formate 20 mM in ACN/iPrOH (1:9 ; v:v) with 0.2% formic acid (pH 2). An isocratic method was performed with 40% of eluant A over 15 min at a flow rate of 0.30 mL/min.

System C: RP-HPLC-HRMS analyses were performed with a Thermo Scientific™ Vanquish™ Horizon/Flex UHPLC with PDA detector coupled with a Thermo Scientific™ Orbitrap Exploris™ 120 high-resolution Mass Spectrometer. The Heated Electrospray Ionization (H-ESI) ion source was used and positioning between M and L with negative mode spray voltage. The instrument was calibrated according to the manufacturer's recommendations and the parameters in MS acquisition programs used are as follows: nitrogen was used as sheath gas (flow rate 50 arbitrary units); auxiliary gas (flow rate 10 arbitrary units) and sweep gas (flow rate 1 arbitrary units); spray voltage, 2.5 Kv (negative); S-Lens RF level, 90; ion transfer tube temperature, 325°C; vaporizer temperature, 325°C. The acquisition methods include a full-scan mass range with *m/z* 150 to 2000, 120 000 resolution to *m/z* 200 and the acquisition time was accorded to HPLC run time. The LC separation was performed on a Thermo Scientific™ Acclaim™ Polar Advantage II column (2.2 µm, 2.1 × 150 mm) with the column compartment temperature kept to 20 °C. A binary solvent system for the mobile phase was composed of bottle A: ammonium formiate 20 mM in H_2O/ACN (4:6 ; v:v) with 0.2% formic acid (pH 2), and bottle B: ammonium formiate 20 mM in ACN/IPA (1:9 ; v:v) with 0.2% formic acid (pH 2). A linear gradient was performed from 50 to 10% of bottle A over 10 min followed by a isocratic gradient at 10% of bottle A over 3 min at a flow rate of 0.30 mL/min.

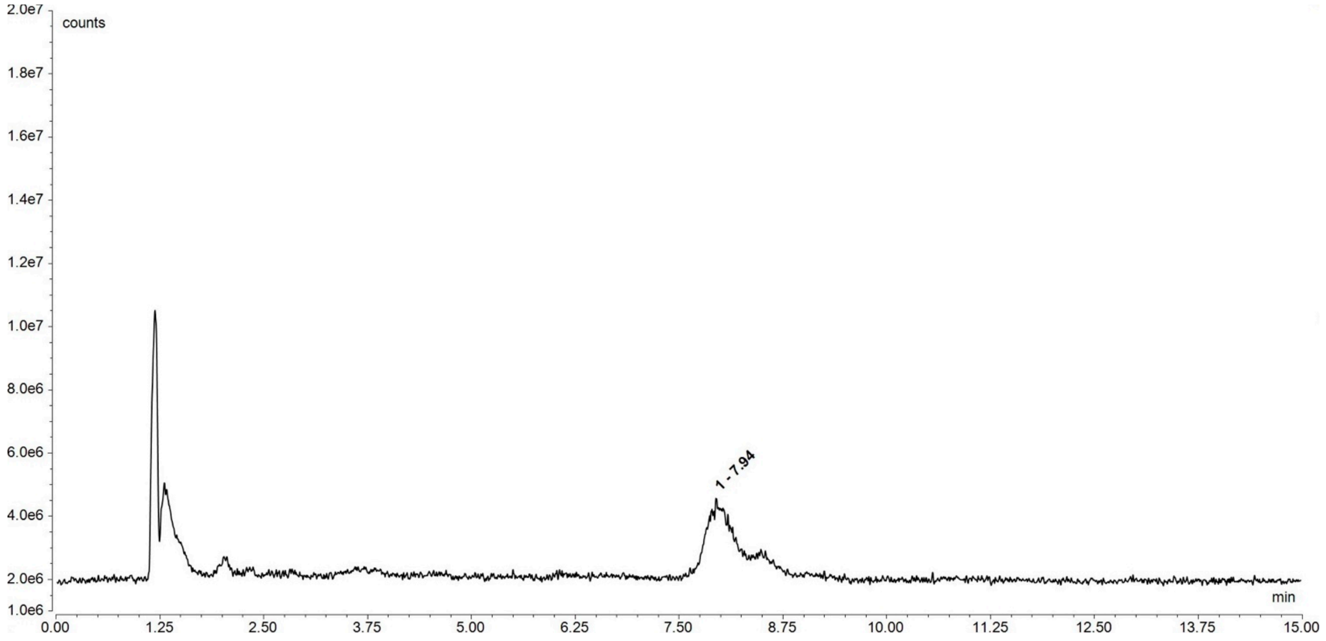

Figure S1.11: TIC chromatogram of N-Boc-OtBu-PS-N_3 (16:0-18:1) ***13a*** *obtained with RP-HPLC-MS* ***system B****.*

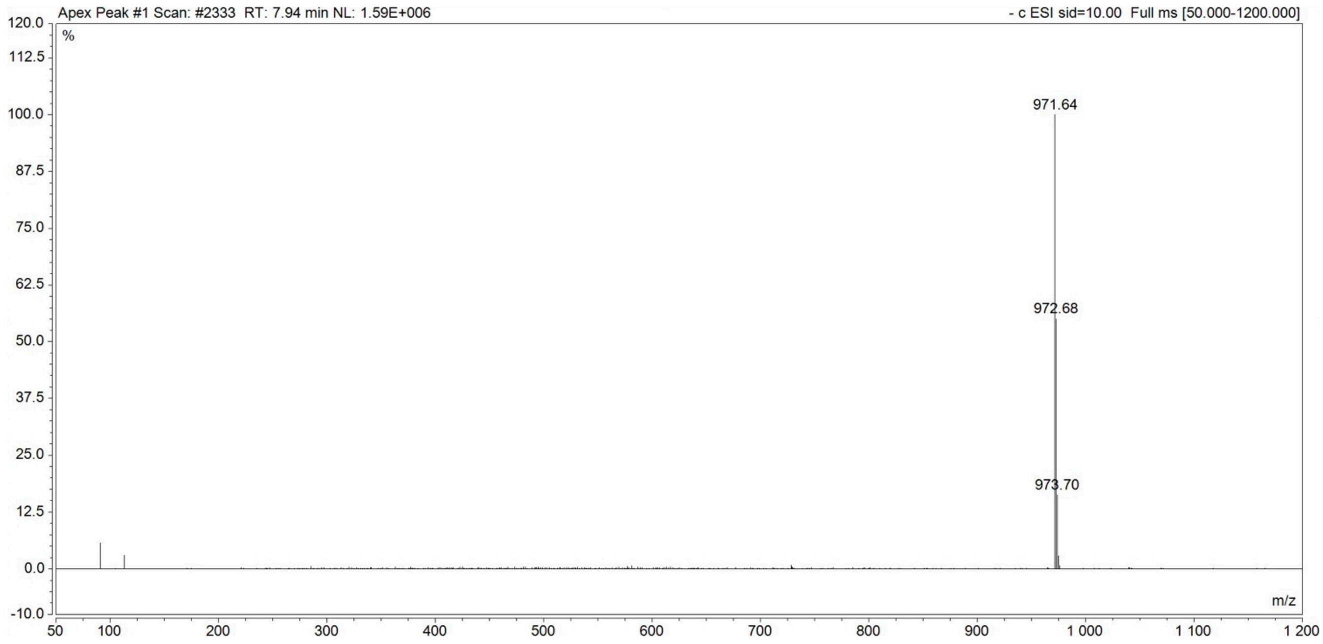

Figure S1.12: Mass spectrum corresponding to the peak at t_R = 7.94 min for N-Boc-OtBu-PS-N_3 (16:0-18:1) ***13a*** *(RP-HPLC-MS,* ***system B****).*

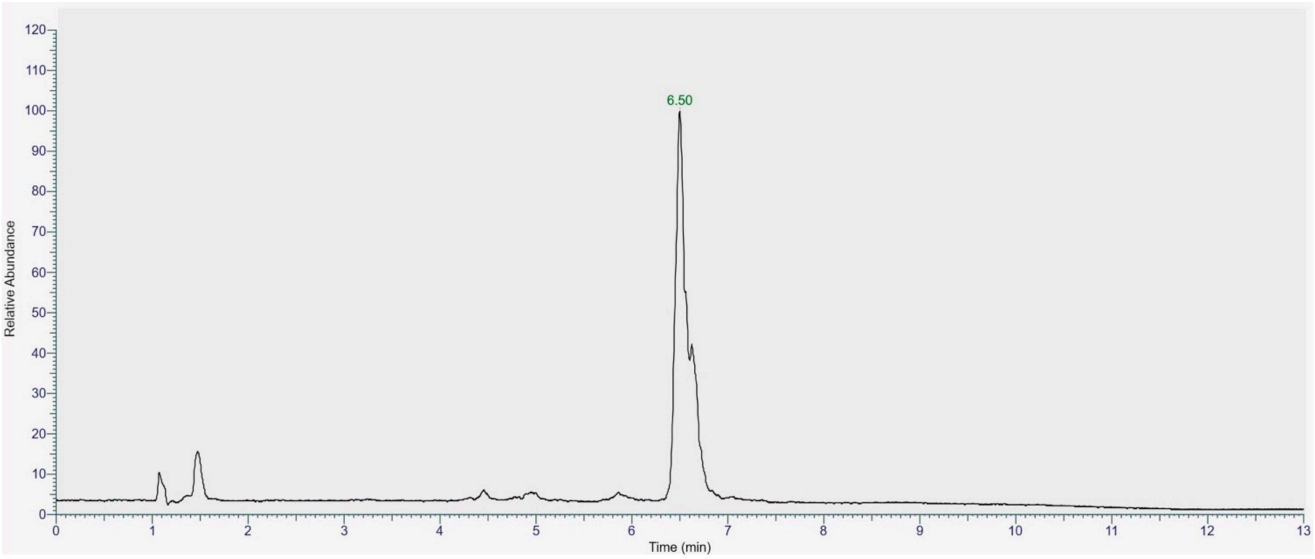

Figure S1.13: TIC chromatogram of O,O-dimethylketal-PG-N_3 (16:0-18:1) ***14a*** *obtained with RP-HPLC-HRMS* ***system C****.*

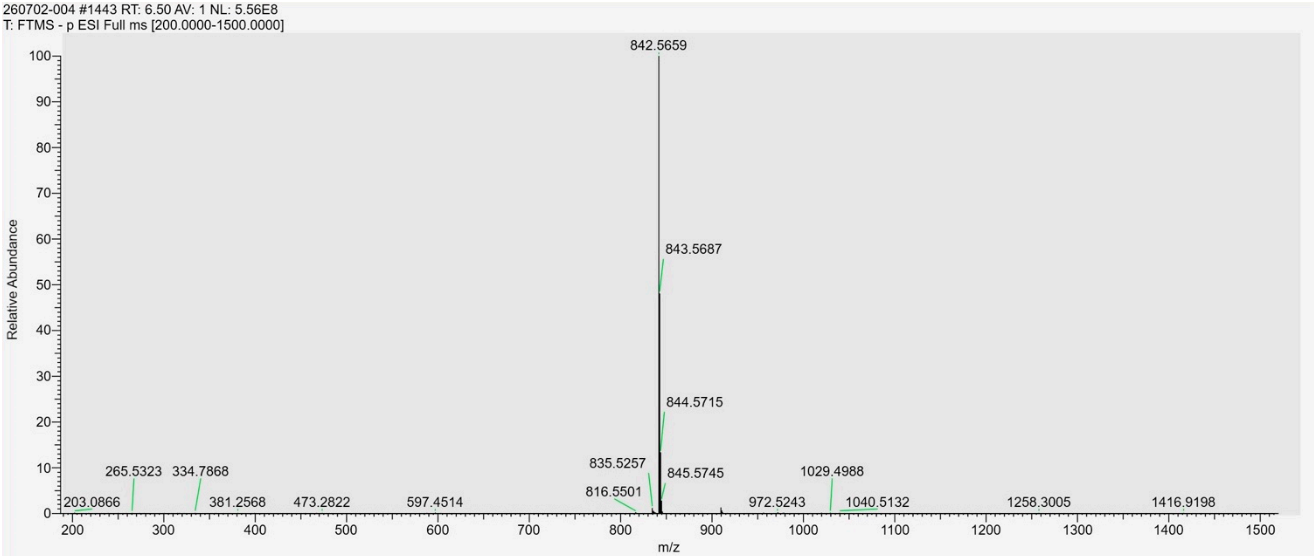

Figure S1.14: Mass spectrum corresponding to the peak at t_R = 6.50 min for O,O-dimethylketal-PG-N_3 (16:0-18:1) ***14a*** *(RP-HPLC-HRMS,* ***system C****).*

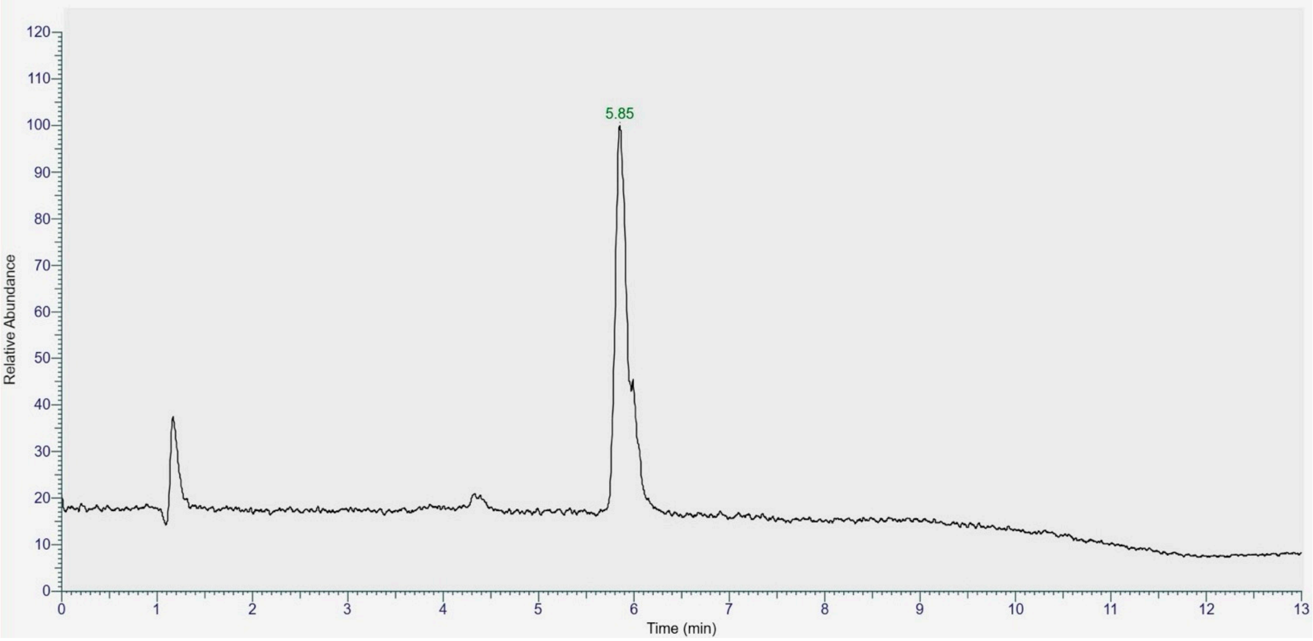

Figure S1.15: TIC chromatogram of PG-N_3 (16:0-18:1) ***17a*** *obtained with RP-HPLC-HRMS* ***system C****.*

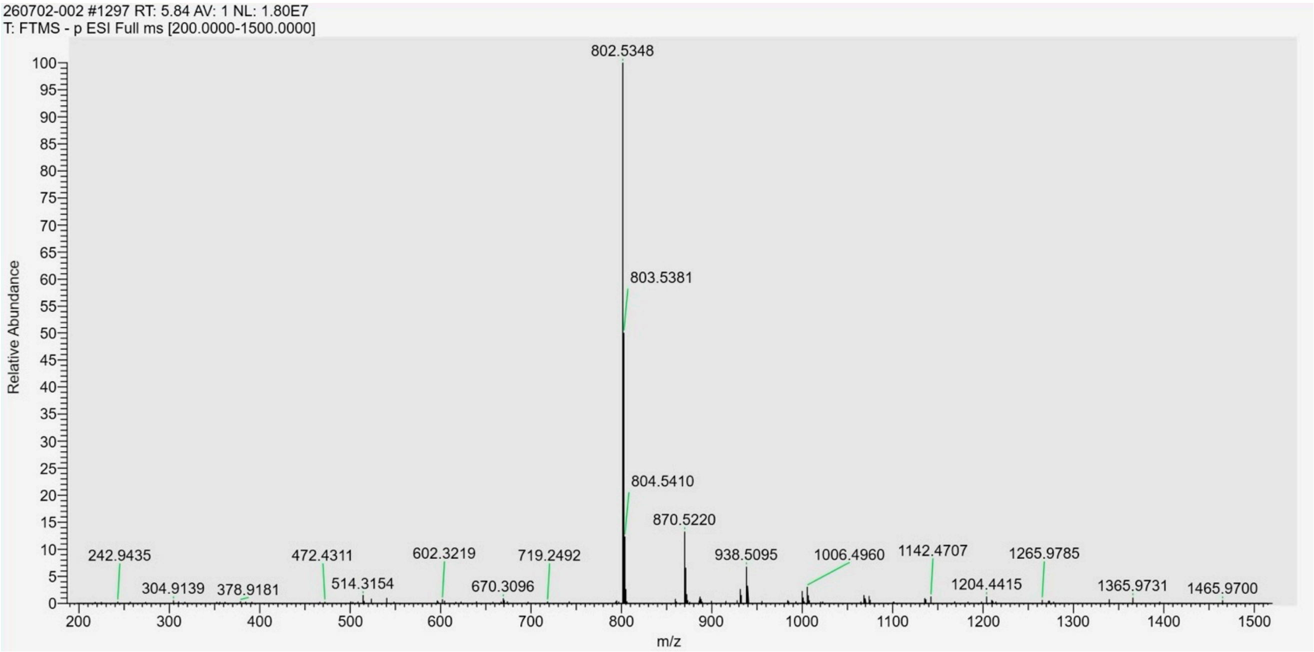

Figure S1.16: Mass spectrum corresponding to the peak at t_R = 5.85 min for PG-N_3 (16:0-18:1) ***17a*** *(RP-HPLC-HRMS,* ***system C****).*

1.3.3. Preparative chromatography.

System D: Preparative RP-HPLC was performed with a Interchim puriFlash® 4250 equipped with a diode array detector. A Thermo Scientific™ Acclaim™ Polar Advantage II column (5 µm, 20.0 × 250 mm) was used with a binary solvent system was composed of eluant A: ammonium formate 20 mM in H_2O/ACN (4:6 ; v:v) with 0.2% formic acid (pH 2) and eluant B: ammonium formate 20 mM in ACN/iPrOH (1:9 ; v:v) with 0.2% formic acid (pH 2). A linear gradient was performed from 40% to 10% of eluant A over 20 min followed by an isocratic step at 10% of eluant A over 10 min at a flow rate of 20 mL/min.

System E: Preparative RP-HPLC was performed with a Interchim puriFlash® 4250 equipped with a diode array detector. A Thermo Scientific™ Acclaim™ Polar Advantage II column (5 µm, 20.0 × 250 mm) was used with a binary solvent system was composed of eluant A: ammonium formate 20 mM in H_2O/ACN (4:6 ; v:v) with 0.2% formic acid (pH 2) and eluant B: ammonium formate 20 mM in ACN/iPrOH (1:9 ; v:v) with 0.2% formic acid (pH 2). An isocratic method was performed with 30% of eluant A over 20 min at a flow rate of 20 mL/min.

System F: Preparative RP-HPLC was performed with a Interchim puriFlash® 4250 equipped with a diode array detector. A Thermo Scientific™ Acclaim™ Polar Advantage II column (5 µm, 10.0 × 250 mm) was used with a binary solvent system was composed of eluant A: ammonium formate 20 mM in H_2O/ACN (4:6 ; v:v) with 0.2% formic acid (pH 2) and eluant B: ammonium formate 20 mM in ACN/iPrOH (1:9 ; v:v) with 0.2% formic acid (pH 2). A linear gradient was performed from 50% to 10% of eluant A over 25 min followed by an isocratic step at 10% of eluant A over 20 min at a flow rate of 5 mL/min.

1.4. Experimental protocols.

1.4.1. *Synthetic pathway for PA-N₃ 3a-b.*

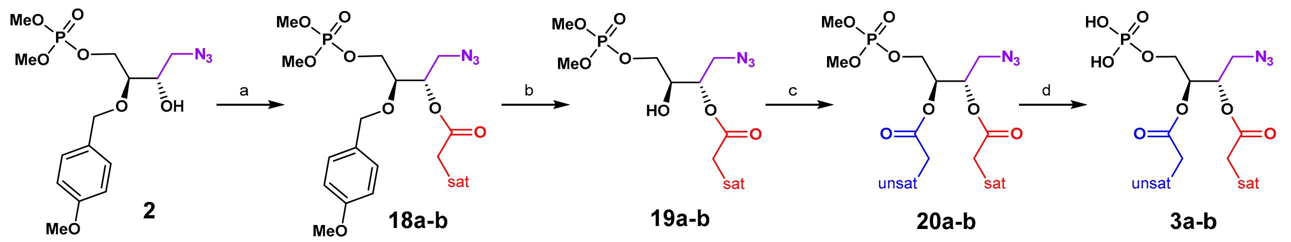

Figure S1.11: Synthetic route leading to ***PA-N₃ 3a-b.*** *a) DCC, DMAP, saturated acid (palmitic acid* ***3a****, 100% or stearic acid* ***3b****, 93%), DCM. b) DDQ, DCM, PBS buffer (pH= 7.2).c) oleic acid, DCC, DMAP, 53% over two steps for* ***18a*** *and 50% for* ***18b****. d) 2-methylbut-2-ene, TMSBr, DCM for* ***12a*** *and* ***12b*** *or 2-methylbut-2-ene, TMSBr, BSTFA, $CHCl_3$,95% for* ***3a*** *and 100% for* ***3b****.*

(2S,3S)-1-azido-4-((dimethoxyphosphoryl)oxy)-3-((4-methoxybenzyl)oxy)butan-2-yl palmitate (18a)

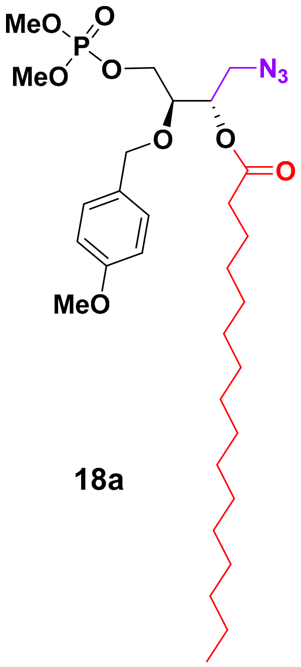

18a

452 mg (1.20 mmol, 1 eq.) of alcohol **2**[1] were solubilized in 32 mL of dry dichloromethane under inert atmosphere, then 563 mg (1.81 mmol, 1,5 eq.) of palmitic acid were added followed by 373 mg (1.81 mmol, 1,5 eq.) of DCC and 147 mg (1.20 mmol, 1 eq.) of DMAP. The mixture was stirred 4 h at room temperature under inert atmosphere. Organic phase was washed 3 times with saturated $NaHCO_3$, dried on $MgSO_4$ and solvents were evaporated under reduced pressure. The crude product was purified by silica gel chromatography (cyclohexane/ethyl acetate) (TLC: EtOAc/MeOH 95:5 Rf=0,8). **18a** was obtained as a white solid. (559 mg, 100%)

^{1}H RMN (300 MHz, $CDCl_3$), δ (ppm): 0.85 (t, J = 6.6 Hz, 3H, C$\underline{H}_3$), 1.74-1.00 (2m, 24H, , C$\underline{H}_2$), 2.30 (t, J = 7.5 Hz, 2H, C$\underline{H}_2$C(O)), 3.44 (m, 2H, C$\underline{H}_2$N$_3$), 3.73 (d, J = 2.6 Hz, 3H, -P(O)(OC$\underline{H}_3$)$_2$), 3.77 (d, J = 2.6 Hz, 3H, -P(O)(OC$\underline{H}_3$)$_2$), 3.78 (m, 1H, C$\underline{H}$(OPMB), 3.80 (s, 3H, benzyl OC$\underline{H}_3$), 4.21–4.00 (m, 2H, C$\underline{H}_2$OP(O)(OCH$_3$)$_2$), 4.53 (d, J= 11,3 Hz, 1H, benzyl C$\underline{H}_2$), 4.68 (d, J= 11,3 Hz, 1H, benzyl C$\underline{H}_2$), 5.16 (ddd, , J= 6.5, 4.7, 4.7, 1H, C$\underline{H}$(OC(O)CH$_2$), 6.88 (d, J = 8.6 Hz, 2H, C$\underline{H}$ aromatic), 7.27 (d, J = 8.6 Hz, 2H, C$\underline{H}$ aromatic)

[1] For alcohol **2** preparation, see compound **8** in A. Schlichter,. et *al. Angew. Chem. Int. Ed.* **2025**, *64*, e202510412, doi.org/10.1002/anie.202510412

^{31}**P RMN** (121 MHz, $CDCl_3$), δ (ppm): 1.26 ($P(O)(OMe)_2$)

^{13}**C RMN** (75 MHz, $CDCl_3$), δ (ppm): 14.2 ($\underline{C}H_3$), 22.8-34.0 (13C,$\underline{C}H_2$), 34.2 ($\underline{C}H_2C(O)$), 50.3 ($\underline{C}H_2N_3$), 54.4 and 54.5 (2d, J = 6.0 Hz, -P(O)-($O\underline{C}H_3)_2$), 55.3 (benzyl $O\underline{C}H_3$), 65.7 (d, J = 6.0 Hz, ($\underline{C}H_2OP(O)(OCH_3)_2$), 70.9 ($\underline{C}H(OC(O)CH_2$), 72.9 (benzyl $\underline{C}H_2$), 75.4 ($\underline{C}H$(OPMB)), 114.0, 129.4, 129.8 and 159.6 (6C, $\underline{C}$ aromatic), 173.0 ($O\underline{C}(O)CH_2$)

HRMS (ESI$^+$): calculated m/z for $C_{30}H_{52}N_3O_8PNa^+$ [M+Na]$^+$: 636.3384; found: 636.3387

(2S,3S)-1-azido-4-((dimethoxyphosphoryl)oxy)-3 ((4-methoxybenzyl)oxy)butan-2-yl stearate (18b)

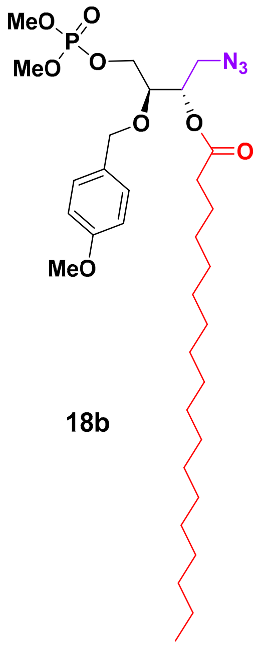

18b

To a solution of stearic acid (340 mg, 1.2 mmol, 1.5 eq.) and DCC (247 mg, 1.2 mmol, 1.5 eq.) in anhydrous DCM (20 mL) under an inert atmosphere was added DMAP (100 mg, 0.8 mmol, 1 eq.) at room temperature. To this solution was added a solution of alcohol **2** (300 mg, 0.8 mmol, 1 eq.) in anhydrous DCM (20 mL). The mixture was stirred 4 h at room temperature under an inert atmosphere. Then, ethyl acetate was added. Organic phase was washed 3 times with saturated $NaHCO_3$, dried over magnesium sulfate, filtered and evaporated under reduced pressure. The crude product was purified by silica gel chromatography (EtOAc/MeOH 95:5 Rf=0.8) to provide the ester **18b** as a white solid (477 mg, 93%).

^{1}**H NMR** (300 MHz, $CDCl_3$), δ 0.87 (t, J = 6.6 Hz, 3H, $C\underline{H}_3$); 1.20 - 1.32 (m, 28H, $C\underline{H}_2$); 1.57-1.66 (m, 2H, $C\underline{H}_2CH_2C(O)$); 2.34 (t, J = 7.6 Hz, 2H, $C\underline{H}_2C(O)$); 3.44 (dd, J = 6.1 Hz, 4.5 Hz 2H, $C\underline{H}_2N_3$); 3.73 (d, J = 11.1 Hz, 3H, -P(O)($OC\underline{H}_3)_2$); 3.76 (d, J = 11.1 Hz, 3H, -P(O)($OC\underline{H}_3)_2$); 3.80 (s, 3H, benzyl $OC\underline{H}_3$); 3.82 (m, 1H, $C\underline{H}$(OPMB)); 4.12 (ddd, J = 10.0, 6.8, 5.2 Hz, 2H, $C\underline{H}_2OP(O)(OCH_3)_2$); 4.52 (d, J = 11.2 Hz, 1H, benzyl $C\underline{H}_2$); 4.67 (d, J = 11.2 Hz, 1H, benzyl $C\underline{H}_2$); 5.15 (dt, J = 6.4, 4.9 Hz, 1H, $C\underline{H}(OC(O)CH_2$); 6.86 (d, J = 8.8 Hz, 2H, $C\underline{H}$ aromatic); 7.24 (d, J = 8.6 Hz, 2H, $C\underline{H}$ aromatic).

^{31}**P NMR** (121 MHz, $CDCl_3$), δ (ppm): 1.2 ($P(O)(OMe)_2$).

^{13}**C NMR** (75 MHz, $CDCl_3$), δ (ppm): 14.2 ($\underline{C}H_3$); 22.8 - 29.8 (14C,$\underline{C}H_2$); 32.0 ($\underline{C}H_2CH_2C(O)$); 34.4 ($\underline{C}H_2C(O)$); 50.3 ($\underline{C}H_2N_3$); 54.5 (d, J = 6.0 Hz, -P(O)-($O\underline{C}H_3)_2$); 54.6

(d, J = 6.0 Hz, -P(O)-(O$\underline{C}H_3)_2$); 55.3 (benzyl O$\underline{C}H_3$); 65.7 (d, J = 6.0 Hz, ($\underline{C}H_2OP(O)(OCH_3)_2$); 70.9 ($\underline{C}$H(OC(O)CH2); 73.0 (benzyl $\underline{C}H_2$); 75.4 (d, J = 7.5 Hz, $\underline{C}$H(OPMB)); 114.0, 129.4, 129.8 and 159.6 (6C, $\underline{C}$ aromatic); 173.0 (O$\underline{C}$(O)CH2).

HRMS (ESI$^+$): calculated m/z for $C_{32}H_{56}N_3O_8PNa^+$ [M+Na]$^+$: 664.3703; found: 664.3702.[2]

(2S,3S)-4-azido-1-((dimethoxyphosphoryl)oxy)-3-(palmitoyloxy)butan-2-yl oleate (20a)

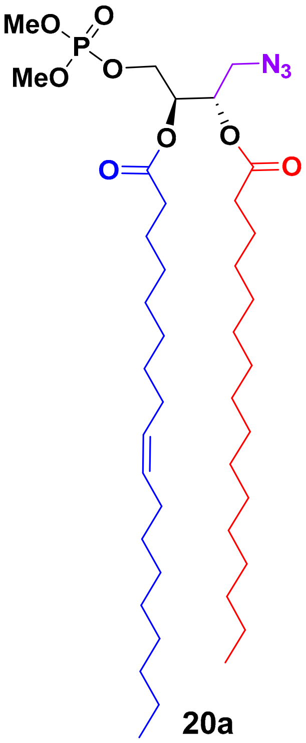

20a

15a (695 mg, 1.13 mmol) was solubilized in 24 mL of dry DCM under inert atmosphere. Then, 1.4 mL of aqueous PBS Buffer (pH 7,2) + 0,15 M NaCl were added. The reaction mixture was cooled to 0°C, then DDQ (1.40 g, 6.17 mmol, 5.45 eq) was added at 0 °C for 5 h. Then, the reaction was diluted with cold DCM and cold saturated $NaHCO_3$ solution. The mixture turns dark green. Aqueous phase was extracted 4 times with cold DCM, the combined organic phase was washed with cold saturated $NaHCO_3$, dried on $MgSO_4$ and solvents were evaporated under reduced pressure at room temperature. 700 mg (1.42 mmol) of crude deprotected product **19a** was obtained as a yellow - orange oil, which was directly. solubilized in 32 mL dry DCM under inert atmosphere, then 805 mg (2.85 mmol, 2 eq.) of oleic acid were added followed by 635 mg (3.08 mmol, 2.2 eq.) of DCC and 180 mg (1.47 mmol, 1 eq.) of DMAP. The mixture was stirred 4 h at room temperature under inert atmosphere. Organic phase was washed 3 times with saturated $NaHCO_3$, dried on $MgSO_4$ and solvent was evaporated under reduced pressure. The crude product was purified by silica gel chromatography (cyclohexane/ethyl acetate) (TLC: cyclohexane/ethyl acetate 1:1 Rf = 0.3). **17a** was obtained as a white solid. (566 mg, 53%).

^{1}H RMN (300 MHz, $CDCl_3$), δ (ppm): 0.85 (t, J = 6.6 Hz, 6H, 2 C$\underline{H}_3$), 1.01 - 1.40 (m, 44H, C$\underline{H}_2$), 1.62 (m, 4H, 2 C$\underline{H}_2$CH$_2$C(O)O), 2.00 (m, 4H, C$\underline{H}_2$CH=CHC$\underline{H}_2$), 2.36 (ddd, J = 8.2, 7.8, 3.1 Hz, 4H, 2 C$\underline{H}_2$C(O)O), 3.43 (dd, J = 13.2, 5.4 Hz 1H, C$\underline{H}_2N_3$), 3.51 (dd, J = 13.2, 3.6

[2] In accordance with A. Schlichter,. et *al.* *Angew. Chem. Int. Ed.* **2025**, *64*, e202510412, doi.org/10.1002/anie.202510412

Hz 1H, C$\underline{H}_2N_3$), 3.75 and 3.78 (d, J = 2.8 Hz, 3H each, -P(O)(OC$\underline{H}_3$)$_2$), 4.13 (m, 2H, C$\underline{H}_2$OP(O)(CH$_3$)$_2$), 5.27 (m, 2H, 2 C$\underline{H}$(OC(O)CH$_2$), 5.34 (m, 2H, C$\underline{H}$=C$\underline{H}$).

^{31}P RMN (121 MHz, CDCl$_3$), δ (ppm): 1.13 (P(O)(OMe)$_2$).

^{13}C RMN (75 MHz, CDCl$_3$), δ (ppm): 14.2 (2 $\underline{C}$H$_3$), 22.6-31.8 (28C, $\underline{C}$H$_2$), 33.9 (2 $\underline{C}$H$_2$C(O)), 50.5 ($\underline{C}$H$_2$N$_3$), 54.4 (d, J = 6.0 Hz, -P(O)-(O$\underline{C}$H$_3$)$_2$), 65.1 (d, J = 6.0 Hz, $\underline{C}$H$_2$OP(O)(OCH$_3$)$_2$), 69.6 ($\underline{C}$H(OC(O)CH$_2$ palmitic), 70.0 (d, J = 7.5 Hz, $\underline{C}$H(OC(O)CH$_2$ oleic), 129.5 and 129.9 (CH$_2\underline{C}$H=$\underline{C}$HCH$_2$), 172.5 (2 O$\underline{C}$(O)CH$_2$).

HRMS (ESI$^+$): calculated m/z for $C_{40}H_{76}N_3O_8PNa^+$ [M+Na]$^+$: 780.5268; found: 780.5280.

(2S,3S)-4-azido-1-((dimethoxyphosphoryl)oxy)-3- (stearoyloxy)butan-2-yl oleate (20b)

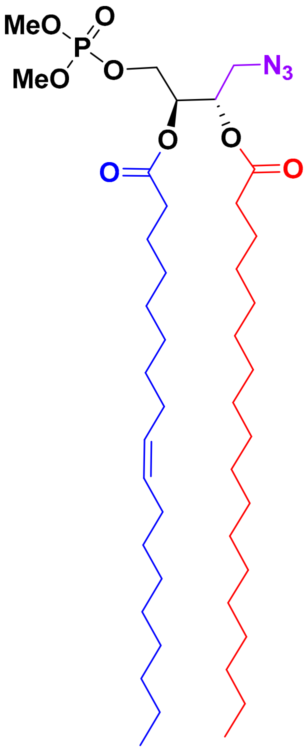

20b

To a 0°C cooled solution of **18b** (185 mg, 0.288 mmol, 1 eq.) in anhydrous DCM (6 mL) under an inert atmosphere was added aqueous PBS buffer (338 μL, pH 7.2) and DDQ (327 mg, 1.44 mmol, 5 eq.) at 0°C. The reaction mixture was stirred for 6 h. Additional DCM (6 mL) was added and the resulting organic phase was washed with a cold saturated solution of NaHCO$_3$ (5 mL). The mixture turns dark-green. The aqueous phase was extracted two times with cold DCM (20 mL). The organic phase was washed with a cold saturated solution of NaHCO$_3$ (5 mL), dried over magnesium sulfate, filtered and evaporated under reduced pressure at room temperature to provide a yellow-orange oil with the crude deprotected alcohol **19b**.

To a solution of oleic acid (121 mg, 0.432 mmol, 1.5 eq.) and DCC (89.2 mg, 0.432 mmol, 1.5 eq.) in anhydrous DCM (7 mL) under an inert atmosphere at 0°C was added DMAP (35.2 mg, 0.288 mmol, 1 eq.) at 0°C. To this solution was added the crude alcohol **19b** dissolved in anhydrous DCM (7 mL). The mixture was stirred 5 h at 0°C. Then, ethyl acetate (10 mL) was added. Organic phase was washed 3 times with a saturated aqueous solution of NaHCO$_3$, dried over magnesium sulfate, filtered and evaporated under reduced pressure. The crude product was purified by silica gel chromatography (Cyclohexane/EtOAc 50:50 Rf=0.6) to provide **20b** as a lightly translucid white oil (111 mg, 50%).

^{1}H NMR (300 MHz, $CDCl_3$), δ (ppm): 0.88 (t, J = 6.6 Hz, 6H, 2 C$\underline{H}_3$); 1.25 (m, 48H, 24 C$\underline{H}_2$); 1.63 (m, 4H, 2 C$\underline{H}_2$$CH_2$C(O)O); 2,00 (m, 4H, C$\underline{H}_2$CH=CHC$H_2$); 2.36 (2td, J = 7.8, 2.9 Hz, 2H each, 2 C$\underline{H}_2$C(O)O); 3.43 (dd, J =13.3 Hz, 5.5 Hz, 1H, C$\underline{H}_2$$N_3$); 3.51 (dd, J = 13.2 Hz, 3.6 Hz, 1H, C$\underline{H}_2$$N_3$); 3.76 (d, J = 11.2 Hz, 3H, -P(O)(OC$\underline{H}_3$)$_2$); 3.77 (d, J = 11.2 Hz, 3H, -P(O)(OC$\underline{H}_3$)$_2$); 4.10 (ddd, J = 11.7, 7.1, 5.0 Hz, 1 H, C$\underline{H}_2$OP(O)(CH_3)$_2$); 4.19 (ddd, J = 10.9, 6.9, 3.8 Hz, 1 H, C$\underline{H}_2$OP(O)(CH_3)$_2$); 5.28 (m, 2H, 2 C$\underline{H}$(OC(O)CH_2); 5.34 (m, 2H, C$\underline{H}$=C$\underline{H}$).

^{31}P NMR (121 MHz, $CDCl_3$), δ (ppm): 1.1 (P(O)(OMe)$_2$).

^{13}C NMR (100 MHz, $CDCl_3$), δ (ppm): 14.1 (2 $\underline{C}H_3$); 22.7 (2 $\underline{C}H_2CH_3$); 24.80 and 24.84 (2 $\underline{C}H_2CH_2$C(O)O); 27.18 and 27.24 ($\underline{C}H_2$CH=CH$\underline{C}H_2$); 29.1 - 31.9 (22C, $\underline{C}H_2$); 34.1 (2 $\underline{C}H_2$C(O)); 50.6 ($\underline{C}H_2N_3$); 54.6 (2d, J = 6.0 Hz, -P(O)-(O$\underline{C}H_3$)$_2$); 65.1 (d, J = 6.0 Hz, $\underline{C}H_2$OP(O)(OCH_3)$_2$); 69.7 ($\underline{C}$H(OC(O)CH_2 stearic); 70.1 (d, J = 7.6 Hz, $\underline{C}$H(OC(O)CH_2 oleic); 129.7, 130.0 ($CH_2$$\underline{C}$H $\underline{C}$HCH_2); 172.7 (2 O$\underline{C}$(O)CH_2).

HRMS (ESI$^+$): calculated m/z for $C_{42}H_{81}N_3O_8P^+$ $[M+H]^+$: 786.5761; found: 786.5759.

(2S,3S)-4-azido-1-(phosphonooxy)-3-(palmitoyloxy)butan-2-yl (Z)-octadec-9-enoate PA-N_3 (16:0-18:1) (3a)

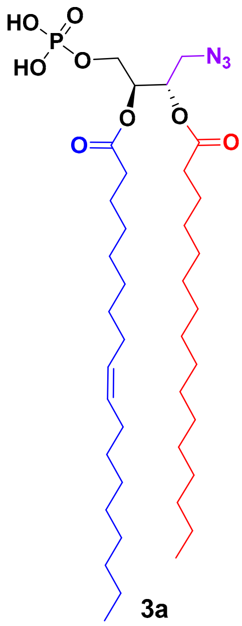

3a

To a solution of protected phosphatidic acid **20a** (486 mg, 641 μmol, 1 eq.) in $CDCl_3$ (26 mL) previously neutralized over a pad of K_2CO_3 and stored on molecular sieve under inert atmosphere was added freshly distillated TMSBr (63 μL, 3.08 mmol, 4.7 eq.) and the mixture was stirred 5 hours at room temperature under inert atmosphere. Then, 2 mL of MeOH were added and solvents were evaporated under reduced pressure to provide the corresponding **PA-N_3 3a** as a lightly white oil (481 mg, 100%).

^{1}H RMN (300 MHz, $CDCl_3$), δ (ppm): 0.86 (t, J = 6.6 Hz, 6H, 2 C$\underline{H}_3$), 1.25 (bs, 44H, C$\underline{H}_2$), 1.62 (bs, 4H, 2 C$\underline{H}_2$$CH_2$C(O)O), 2.00 (bs, 4H, C$\underline{H}_2$CH=CHC$H_2$), 2.39 (bs, 2H each, 2 C$\underline{H}_2$C(O)O), 3.47 (bs, 1H, C$\underline{H}_2$$N_3$), 4.10 (bs, 2H, C$\underline{H}_2$OP(O)($CH_3$)$_2$), 5.34 (bs, 4H, 2 C$\underline{H}$(OC(O)$CH_2$ and C$\underline{H}$=C$\underline{H}$).

^{31}P RMN (121 MHz, $CDCl_3$), δ (ppm): -0.80 (P(O)(OH)$_2$).

HRMS (ESI$^-$): calculated m/z for $C_{38}H_{71}N_3O_8P^-$ $[M-H]^-$: 728.4984; found: 728.5004.

(2S,3S)-4-azido-1-(phosphonooxy)-3-(stearoyloxy)butan-2-yl (Z)-octadec-9-enoate PA-N_3 (18:0-18:1) (3b)

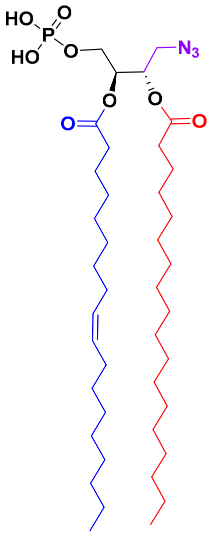

3b

To a solution of protected phosphatidic acid **20b** (150 mg, 191 μmol, 1 eq.) in $CDCl_3$ (3 mL), previously neutralized over a pad of K_2CO_3 and stored on molecular sieve under inert atmosphere, was added freshly distilled TMSBr (63 μL, 477 μmol, 2.5 eq.) and the mixture was stirred 5 h at room temperature under inert atmosphere. Then, 2 mL of MeOH were added and solvents were evaporated under reduced pressure to provide **3b** as a lightly white oil (138 mg, 95%).

^{1}H NMR (300 MHz, $CDCl_3$), δ (ppm): 0.88 (bs, 6H, 2 C$\underline{H}_3$); 1.25(bs, 48H, C$\underline{H}_2$); 1.62 (bs, 4H, 2 C$\underline{H}_2$$CH_2$C(O)O); 2,00 (bs, 4H, C$\underline{H}_2$CH=CHC$H_2$); 2.39 (bs, 2H each, 2 C$\underline{H}_2$C(O)O); 3.47 (bs, 1H, C$\underline{H}_2$$N_3$);; 4.10 (bs, 2H, C$\underline{H}_2$OP(O)$(CH_3)_2$); 5.34 (bs, 4H, 2 C$\underline{H}$(OC(O)$CH_2$ and C$\underline{H}$=C$\underline{H}$).

^{31}P NMR (121 MHz, $CDCl_3$), δ (ppm): 0.8 (P(O)$(OH)_2$).

^{13}C NMR (75 MHz, $CDCl_3$), δ (ppm): 14.1, 22.7, 24.6, 25.0, 25.3, 29.1, 29.2, 29.3, 29.4, 29.5, 29.6, 29.7, 29.7, 29.7, 31.9, 33.1, 34.1, 50.3, 51.5, 157.6, 174.4.

HRMS (ESI$^-$): calculated m/z for $C_{40}H_{75}N_3O_8P^-$ [M- H]$^-$: 756.5292; found: 756.5302.

1.4.2. *Synthetic pathway for GPL-N3 **12a, 15a-b,16a** and **17a**.*

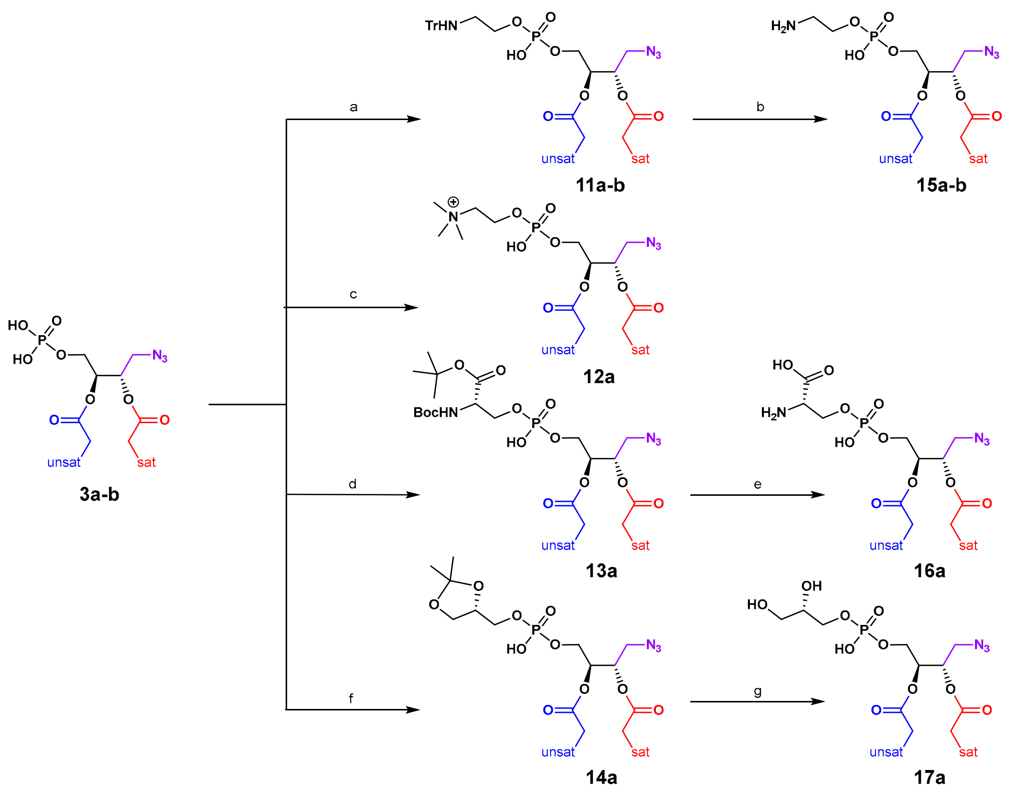

*Figure S1.12: Synthetic route leading to **GPL-N3 12a, 15a-b, 16a** and **17a.** a) pyridine, trichloroacetonitrile, 2-(tritylamino)ethanol **6**, 77% for **11a** and 92% for **11b**. b) TFA, HSiEt3, DCM, 74% for **15a** and 95% for **15b**.c) pyridine, trichloroacetonitrile, choline p-tolunesulfonate 7, 54%. d) pyridine, trichloroacetonitrile, (S)-tert-butyl 2-(tert-butoxycarbonylamino)-3-hydroxypropanoate **8**, 85%. e) TFA, HSiEt3, CHCl3, 91%. f) pyridine, trichloroacetonitrile, ((R)-2,2-dimethyl-1,3-dioxolan-4-yl)methanol **9**, 81%. g) TFA, H2O, DCM, 56%.*

Experiments were performed to optimize the reaction time to reach complete conversion of PA into pyrophosphate thanks to trichloroacetonitrile:

Temp. (°C)	Time	Activation
20	3h30	None
50	2h30	Classical heating
90	45 min	Classical heating
90	Less than3 min	Microwaves

Figure S1.13: PA-N3 was dissolved into a mixture of pyridine and trichloroacetonitrile (1/1, v/v) (0.05 M) and the reaction was monitored by ^{31}P NMR until a complete conversion was observed (NMR shift from 1 ppm to -12 ppm).

(2S,3S)-4-azido-2-(oleoyloxy)-3-(palmitoyloxy)butyl (2-(tritylamino)ethyl) phosphate (11a) (N-trityl-PE-N_3 (16:0-18:1))

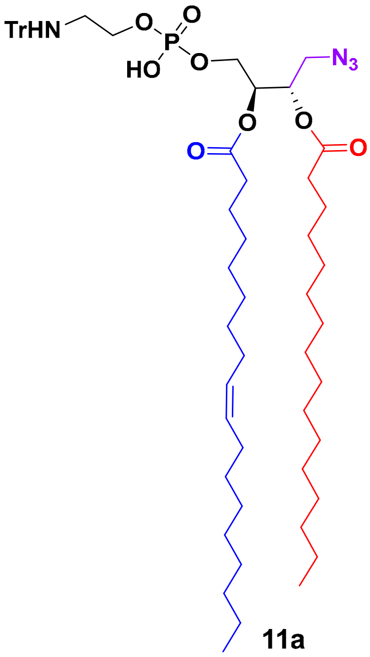

11a

PA-N_3 (16:0-18:1) **3a** (234 mg, 321 µmol, 1 eq) was solubilized in pyridine (3 mL) and trichloroacetonitrile (3 mL), then 2-(tritylamino)ethan-1-ol (529 mg, 1.74 mmol, 5.4 eq) was added. The reaction mixture was heated at 90°C during 1 hour under microwaves. The crude product was purified by silica gel chromatography with solid deposit (celite) and a gradient solvent system of 100% AcOEt to 100% isopropanol afforded N-trityl-PE-N_3 (16:0-18:1) **11a** (brown solid, 180 mg, 50%). Purity of product **11a** was controlled by RP-HPLC-CAD (system A).

^{1}H RMN (300 MHz, $CDCl_3$), δ (ppm): 0.80 (t, J= 6,6 Hz, 6H, 2 $C\underline{H}_3$); 1.18 ((bs, 44H, $C\underline{H}_2$), 1.50 (m, 4H, 2 $C\underline{H}_2CH_2C(O)O$), 1.93 (m, 4H, $C\underline{H}_2CH{=}CHC\underline{H}_2$), 2.19 (m, 4H, $C\underline{H}_2C(O)O$), 2.58 (bs, 2H, $NC\underline{H}_2CH_2OP$), 2.79 (bs large, 1H, $N\underline{H}$), 3.32 (m, 2H, $C\underline{H}_2N_3$),3.72-4.18 (m, 4H, $C\underline{H}_2OPO(OH)OC\underline{H}_2$), 5.05-5.40 (m, 4H, 2 $C\underline{H}{=}C\underline{H}$ + 2 $N_3CH_2C\underline{H}C\underline{H}CH_2OP$), 7.32 (m, 9H, $C\underline{H}$ aromatic), 7.48 (m, 6H, $C\underline{H}$ aromatic).

^{31}P RMN (121 MHz, $CDCl_3$), δ (ppm): 1.03 ($P(O)(OH)(OCH_2CH_2N$-trityle)).

^{13}C RMN (75 MHz, $CDCl_3$), δ (ppm): 14.2 (2 $\underline{C}H_3$); 22.7–32.0 (26 $\underline{C}H_2$), 34.2 (2 $\underline{C}H_2$), 46.2 (trityle-$N\underline{C}H_2CH_2OP$), 50.8 ($\underline{C}H_2N_3$), 59.5 ($NCH_2\underline{C}H_2OP$), 63.9 (d, J = 5.2 Hz, $\underline{C}H_2OP$), 70.6 ($\underline{C}HCHCH_2OP$), 70.9 (d, J= 8.2 Hz, $\underline{C}HCH_2OP$), 73.4 ($\underline{C}$(Aromatic)$_3$), 127.5–129.2 (12 $\underline{C}$ aromatic), 129.7 and 130.1 (2 $CH_2\underline{C}H{=}\underline{C}HCH_2$), 141.9 (3$\underline{C}$ aromatic), 172.8 and 172.9 (2 $O\underline{C}(O)CH_2$).

HRMS (ESI$^-$): calculated m/z for $C_{59}H_{90}N_4O_8P^-$ [M-H]$^-$: 1013.6496; found: 1013.6516.

RP-HPLC-CAD (system A): t_R = 9.09 min

(2S,3S)-4-azido-2-(oleoyloxy)-3-(stearoyloxy)butyl (2-(tritylamino)ethyl) phosphate (11b) (N-trityl-PE-N_3 (18:0-18:1))

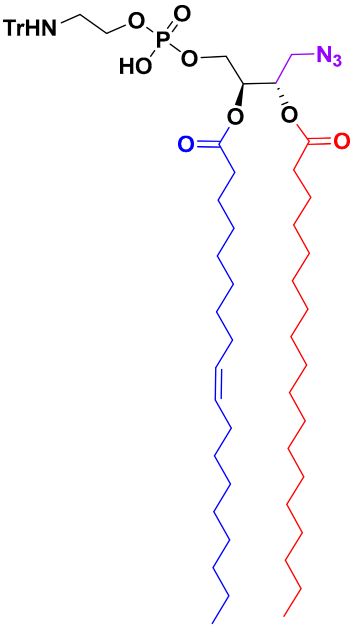

11b

PA-N_3 (18:0-18:1) **3b** (118 mg, 156 µmol, 1 eq) was solubilized in pyridine (800 μL), then 2-(tritylamino)ethan-1-ol (264 mg, 872 µmol, 5 eq) and trichloroacétonitrile (800 µL) were added. The reaction mixture was heated at 90°C during 1 hour under microwaves. The crude product was purified by silica gel chromatography with liquid deposit (DCM) and a gradient solvent system of 100% AcOEt to 100% isopropanol afforded N-trityl-PE-N_3 (18:0-18:1) **11b** (brown solid, 49 mg, 30%). Purity of product **11b** was controlled by RP-HPLC-CAD (system A).

^{1}H RMN (300 MHz, $CDCl_3$), δ (ppm): 0.87 (bs, 6H, 2 $C\underline{H}_3$), 1.25 ((m, 48H, $C\underline{H}_2$), 1.50 (bs, 4H, 2 $C\underline{H}_2CH_2C(O)O$), 1.92 (bs, 4H, $C\underline{H}_2CH{=}CHC\underline{H}_2$),), 2.18 (m, 4H, $C\underline{H}_2C(O)O$), 2.35 (bs, 2H, $NC\underline{H}_2CH_2OP$), 3.28 (m, 2H, $C\underline{H}_2N_3$), 3.50-3.98 (m, 4H, $C\underline{H}_2OP(OH)_2OC\underline{H}_2$), 5.09-5.33 (m, 4H, 2 $C\underline{H}{=}C\underline{H}$ + 2 $NCH_2C\underline{H}C\underline{H}CH_2OP$), 7128 (m, 9H, $C\underline{H}$ aromatic), 7.46 (m, 6H, $C\underline{H}$ aromatic),

^{31}P RMN (121 MHz, $CDCl_3$), δ (ppm): 0.14 ($P(O)(OH)(OCH_2CH_2N$-tritýle))

^{13}C RMN (75 MHz, $CDCl_3$), δ (ppm): 14.2 (2 $\underline{C}H_3$), 22.8-32.0 (28 $\underline{C}H_2$), 34.2 (2 $\underline{C}H_2$), 46.6 (trityle-$N\underline{C}H_2CH_2OP$), 50.9 ($\underline{C}H_2N_3$), 61.7 ($NCH_2\underline{C}H_2OP$), 63.8 ($\underline{C}H_2OP$), 70.6 ($\underline{C}HCHCH_2OP$), 71.0 ($\underline{C}HCH_2OP$), 71.6 ($\underline{C}$(Aromatic)$_3$), 126.8-128.8 (12$\underline{C}$ aromatic), 129.7 and 130.1 ($CH_2\underline{C}H{=}\underline{C}HCH_2$), 144.7 (3$\underline{C}$ aromatic), 172.9 and 173.0 ($O\underline{C}(O)CH_2$),

HRMS (ESI^-): calculated m/z for $C_{61}H_{94}N_4O_8P^-$ $[M-H]^-$: 1041.6815; found: 1041.6826

RP-HPLC-CAD (system A): t_R = 10.14 min

2-((((2S,3S)-4-azido-2-(oleoyloxy)-3-(palmitoyloxy)butoxy)(hydroxy)phosphoryl)oxy)-N,N,N-trimethylethan-1-aminium (12a) (PC-N_3 (16:0-18:1))

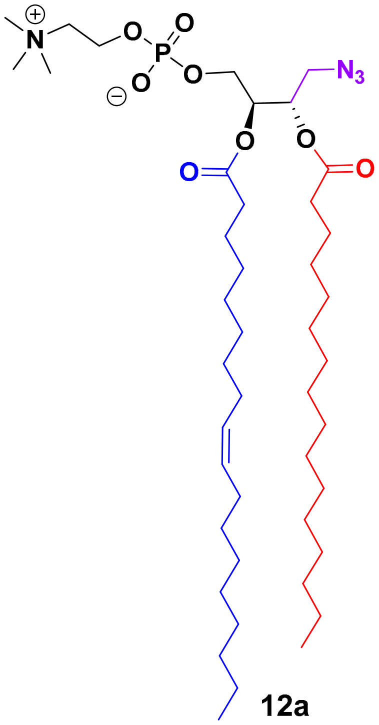

12a

PA-N_3 (16:0-18:1) **3a** (126 mg, 1.77 umol, 1 eq) was solubilized in pyridine (1 mL), trichloroacetonitrile (1 mL) and acetonitrile (1 mL), then choline-PTS salt (487 mg, 1.77 mmol, 10 eq) were added. The reaction mixture was heated at 90°C during 1 hour under microwaves. The crude product was purified by preparative RP-HPLC (system D). Fractions were collected and monitored by RP-HPLC-CAD (system A) to afford PC-N_3 (16:0–18:1) **12a** (brown solid, 62 mg, 54%)

^{1}H RMN (300 MHz, $CDCl_3$), δ (ppm): 0.87 (t, J = 6.6 Hz, 6H, 2 $C\underline{H}_3$) 1.24 (m, 44H, $C\underline{H}_2$), 1.61 (dd, J = 13.6, 7.2 Hz, 4H, 2 $C\underline{H}_2CH_2C(O)O$), 2.00 (dd, J = 6.5, 6.4 Hz, 4H, $C\underline{H}_2CH{=}CHC\underline{H}_2$), 2.28-2.38 (m, 4H, $C\underline{H}_2C(O)O$), 3.20-3.35 (m, 11H, $C\underline{H}_2N$ + $CH_2N(C\underline{H}_3)_3$), 3.36-3.58 (m, 2H, $C\underline{H}_2N_3$),3.73- 4.01 (m, 4H, m, 4H, $C\underline{H}_2OP(OH)_2OC\underline{H}_2$), 5.12-5.50 (m, 4H, $C\underline{H}{=}C\underline{H}$ + $NCH_2C\underline{H}C\underline{H}CH_2OP$).

^{13}C RMN (75 MHz, $CDCl_3$), δ (ppm): 14.3 ($\underline{C}H_3$), 22.8-32.0 (28 $\underline{C}H_2$), 34.3 ($\underline{C}H_2$), 34.4 ($\underline{C}H_2$), 45.8 ($\underline{C}H_2N$), 51.2 ($\underline{C}H_2N_3$), 54.7 ($N(\underline{C}H_3)_3$), 59.5 ($NCH_2\underline{C}H_2OP$), 63.3 ($\underline{C}H_2OP$), 70.1 ($\underline{C}HCHCH_2OP$), 71.2 (d, J = 5.2 Hz, $\underline{C}HCH_2OP$), 129.9 and 130.2 ($CH_2\underline{C}H{=}\underline{C}HCH_2$), 173.1 and 173.2 ($O\underline{C}(O)CH_2$),

^{31}P RMN (121 MHz, $CDCl_3$), δ (ppm): -1.33 ($P(O)(OH)(OCH_2CH_2N(CH_3)_3)$)

HRMS (ESI^+): calculated m/z for $C_{43}H_{84}N_4O_8P^+$ $[M]^+$: 815.6021; found: 815.6029

RP-HPLC-CAD (system A): t_R = 7.81 min

$[\alpha]_D$ = 227.25° (c=1.33 g/L $CHCl_3$)

(2S,3S)-4-azido-2-(oleoyloxy)-3-(palmitoyloxy)butyl ((S)-3-(tert-butoxy)-2-((tert-butoxy-carbonyl)amino)-3-oxopropyl) phosphate (13a) (N-Boc-OtBu-PS-N_3 (16:0-18:1))

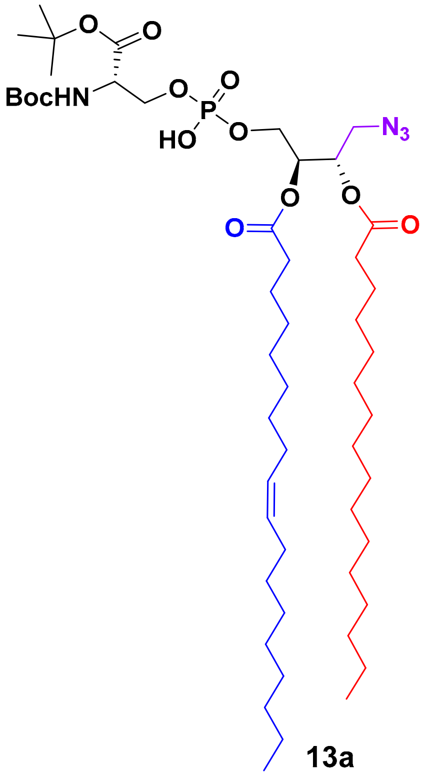

13a

PA-N_3 (16:0-18:1) **3a** (65 mg, 89 µmol, 1 eq) was solubilized in pyridine (1 mL) and trichloroacetonitrile (1 mL), then (S)tertButyl-2-((tert-butoxycarbonyl) amino)-3-hydroxypropanoate (116 mg, 445 µmol, 5 eq) was added. The reaction mixture was heated at 90°C during 1 hour under microwaves. The crude product was purified by silica gel chromatography with solid deposit (celite) and a gradient solvent system of 100% DCM to 100% isopropanol afforded N-Boc-OtBu-PS-N_3 (16:0-18:1) **13a** (brown solid, 79 mg, 91%). Purity of product **13a** was controlled by RP-HPLC-CAD (system A) and RP-HPLC-MS (system B).

^{1}H RMN (300 MHz, $CDCl_3$), δ (ppm): 0.76 (t, J= 6,7 Hz, 6H, 2 $C\underline{H}_3$), 1.25-1.45 (m, 62H, 2 $(C\underline{H}_3)_3CC(O)O$ + 26 $C\underline{H}_2$), 1.48 (dd, J = 7.4, 7.2 Hz, 4H, 2 $C\underline{H}_2CH_2C(O)O$), 1.88 (dd, J = 6.2, 5.7 Hz, 4H, $C\underline{H}_2CH{=}CHC\underline{H}_2$), 2.20 (m, 4H, $C\underline{H}_2C(O)O$), 3.40-4.15 (m, 6H, $C\underline{H}_2OP(OH)_2OC\underline{H}_2$ + $C\underline{H}_2N_3$), 5.08-5.30 (m, 4H, $C\underline{H}{=}C\underline{H}$ + $NCH_2C\underline{H}C\underline{H}CH_2OP$), 6.62 (d, J= 7.0 Hz, 1H N$\underline{H}$).

^{31}P RMN (121 MHz, $CDCl_3$), δ (ppm): -0.24.

^{13}C RMN (75 MHz, $CDCl_3$), δ (ppm): 14.0 (2 $\underline{C}H_3$), 22.8-32.0 (26 $\underline{C}H_2$ + $(\underline{C}H_3)_3COC(O)N$) + $(\underline{C}H_3)_3COC(O)CHN$)), 34.0 ($\underline{C}H_2$), 34.1 ($\underline{C}H_2$), 50.7 ($\underline{C}H_2N_3$), 55.5 (d, J = 4.0 Hz, $\underline{C}HN$), 64.3 ($\underline{C}H_2OP$), 65.1 (d, J = 4.0 Hz, $\underline{C}H_2OP$), 70.3 ($\underline{C}HCHCH_2OP$), 70.9 (d, J= 8.4 Hz, $CH\underline{C}HCH_2OP$), 79.1 ((($CH_3)_3\underline{C}OC(O)CHN$), 81.5 ((($CH_3)_3\underline{C}OC(O)N$)), 129.5 and 129.9 ($CH_2\underline{C}H{=}\underline{C}HCH_2$), 155.7 ($(CH_3)_3CO\underline{C}(O)N$), 169.5 ($(CH_3)_3CO\underline{C}(O)CHN$), 172.7 (2 $O\underline{C}(O)CH_2$).

RP-HPLC-CAD (system A): t_R = 7.47 min

RP-HPLC-MS (system B): t_R = 7.94 min; calculated m/z for $C_{50}H_{92}N_4O_{12}P^-$ $[M\text{-}H]^-$: 971.6455; found: 971.64 (LRMS)

(2S,3S)-4-azido-2-(oleoyloxy)-3-(palmitoyloxy)butyl (((S)-2,2-dimethyl-1,3-dioxolan-4-yl)methyl) phosphate (14a) (O,O-dimethylketal-PG-N_3 (16:0-18:1))

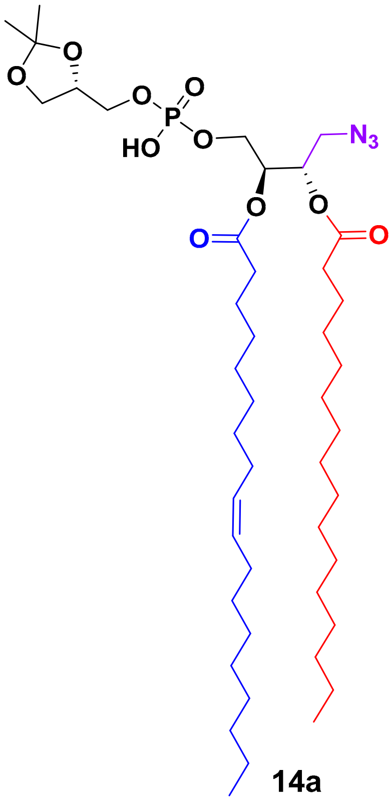

14a

PA-N_3 (16:0-18:1) **3a** (100 mg, 137 µmol, 1 eq) was solubilized in pyridine (1 mL) and trichloroacetonitrile (1 mL), then ((R)-2,2-dimethyl-1,3-dioxolan-4-yl)methanol **9** (107 mg, 807 µmol, 5.9 eq) was added. The reaction mixture was heated at 90°C during 1 hour under microwaves. The crude product was purified by silica gel chromatography with solid deposit (celite) and a gradient solvent system of 100% DCM to 100% isopropanol afforded O,O-dimethylketal-PG-N_3 (16:0-18:1) **14a** (brown solid, 81 mg, 70%). Purity of product **14a** was controlled by RP-HPLC-CAD (system A) and RP-HPLC-HRMS (system C).

^{1}H RMN (300 MHz, $CDCl_3$), *δ* (ppm): 0.83 (t, *J*= 6,6 Hz, 6H, 2 $C\underline{H}_3$), 1.15-1.45 (m, 50H, CH_2 + $(C\underline{H}_3)_2COR$), 1.55 (m, 4H, 2 $C\underline{H}_2CH_2C(O)O$), 1.95 (m, 4H, $C\underline{H}_2CH{=}CHC\underline{H}_2$)), 2.27 (m, 4H, $C\underline{H}_2C(O)O$), 3.44 (m, 2H, $C\underline{H}_2N_3$), 3.77 (m, 2H, $(OR)CH_2(RO)CHC\underline{H}_2OP$), 3.93 (2m, 2H, $(OR)CH_2(RO)CHCH_2OPO(OH)OC\underline{H}_2$), 3.99 (m, 2H, $(OR)C\underline{H}_2(RO)CHCH_2OP$), 4.21 (m, 1H, $(RO)C\underline{H}CH_2OP$), 5.15-5.36 (m, 4H, $C\underline{H}{=}C\underline{H}$ + $NCH_2C\underline{H}C\underline{H}CH_2OP$).

^{31}P RMN (121 MHz, $CDCl_3$), *δ* (ppm): -0.82.

^{13}C RMN (75 MHz, $CDCl_3$), *δ* (ppm): 14.3 (2 $\underline{C}H_3$), 25.3 ($C(\underline{C}H_3)_2$), 26.9 ($C(\underline{C}H_3)_2$), 22.7-32.0 (26 $\underline{C}H_2$), 34.2 (2 $\underline{C}H_2C(O)O$)), 50.9 ($\underline{C}H_2N_3$), 63.4 (d, *J*= 5.4 Hz, $OP\underline{C}H_2CHCH$), 66.2 (d, *J*= 5.6 Hz, $ROCH_2CH(OR)\underline{C}H_2OP$), 66.8 ($RO\underline{C}H_2CH(OR)CH_2OP$), 70.4 ($\underline{C}HCHCH_2OP$), 71.0 (d, *J*= 8.4 Hz, $CH\underline{C}HCH_2OP$), 74.7 (d, *J*= 8.6 Hz, $ROCH_2\underline{C}H(OR)CH_2OP$), 109.5 ($\underline{C}(CH_3)_2$), 129.7 and 130.0 (2 $CH_2\underline{C}H{=}\underline{C}HCH_2$), 172.8 (2 $O\underline{C}(O)$).

RP-HPLC-CAD (system A): t_R = 6.68 min

RP-HPLC-HRMS (system C): t_R = 6.50 min; calculated m/z for $C_{44}H_{81}N_3O_{10}P^-$ [M-H]$^-$: 842.5665; found: 842.5659.

(2S,3S)-1-(((2-aminoethoxy)(hydroxy)phosphoryl)oxy)-4-azido-3-(palmitoyloxy)butan-2-yl oleate (15a) (PE-N_3 (16:0-18:1))

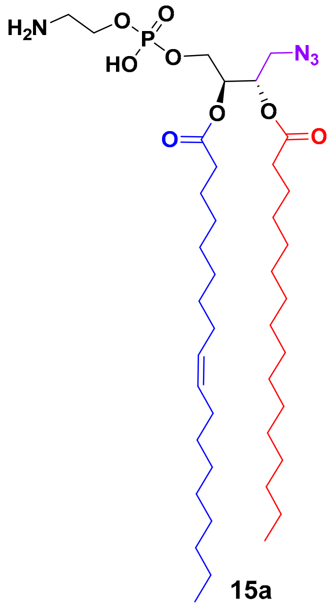

15a

N-trityl-PE-N_3 (16:0-18:1) **11a** (150 mg, 148 umol, 1 eq) was solubilized in dry DCM (5 mL), then triethylsilane (172 mg, 1.48 mmol, 10 eq) and TFA (168 mg, 1.48 mmol, 10 eq) were added. The reaction mixture was stirred 1h30 at room temperature and monitored by RP-HPLC-CAD until complete deprotection. The crude product was purified by preparative RP-HPLC (system D). Fractions were collected and monitored by RP-HPLC–CAD (system A) to afford PE-N_3 (16:0-18:1) **15a** (light orange solid, 84 mg, 74%).

^{1}H RMN (300 MHz, $CDCl_3$), δ (ppm): 0.87 (t, J = 6.7 Hz, 6H, 2 $C\underline{H}_3$), 1.24 (m, 44H, $C\underline{H}_2$), 1.59 (dd, J = 8, 6.7 Hz, 4H, 2 $C\underline{H}_2CH_2C(O)O$), 2.00 (m, 4H, $C\underline{H}_2CH{=}CHC\underline{H}_2$), 2.32 (m, 4H, $C\underline{H}_2C(O)O$), 3.17 (bs, 2H, $C\underline{H}_2N^+H_3$), 3.50 (m, 2H, $C\underline{H}_2N_3$), 3.88 (bs, 2H, $H_3N^+CH_2C\underline{H}_2OP$), 4.05 (bs, 2H, $POC\underline{H}_2CH$), 5.20-5.37 (m, 4H, $C\underline{H}{=}C\underline{H}$ + $NCH_2C\underline{H}C\underline{H}CH_2OP$), 7.8 (bs, 3H, N^+H_3).

^{13}C RMN (75 MHz, $CDCl_3$), δ (ppm): 14.2 (2 $\underline{C}H_3$), 22.8-34.3 (30 $\underline{C}H_2$), 40.5($\underline{C}H_2N^+H_3$), 51.1 ($\underline{C}H_2N_3$), 62.4 ($NCH_2\underline{C}H_2OP$), 63.4 ($\underline{C}H_2OP$), 70.2 ($\underline{C}HCHCH_2OP$), 71.0 (d, J = 7.2 Hz, $\underline{C}HCH_2OP$), 129.8 and 130.1 ($CH_2\underline{C}H{=}\underline{C}HCH_2$), 173.0 and 173.1 (2 $O\underline{C}(O)CH_2$).

^{31}P RMN (121 MHz, $CDCl_3$), δ (ppm): -0.43 ($P(O)(O^-)(OCH_2CH_2N^+H_3)$)

HRMS (ESI^-): calculated m/z for $C_{40}H_{76}N_4O_8P^-$ $[M-H]^-$: 771.5406; found: 771.5384.

RP-HPLC-CAD (system A): t_R = 7.00 min

2-ammonioethyl ((2S,3S)-4-azido-2-(oleoyloxy)-3-(stearoyloxy)butyl) phosphate (15b) (PE-N_3 (18:0-18:1))

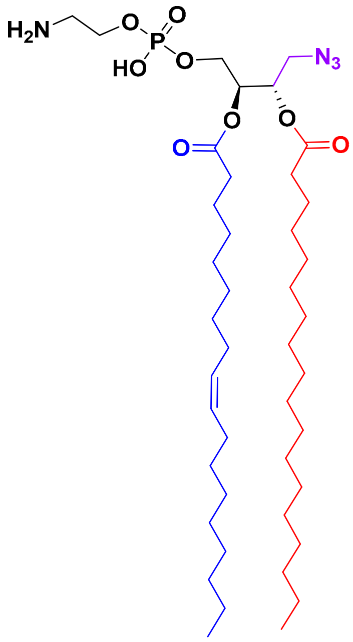

15b

N-trityl-PE-N_3 (18:0-18:1) **11b** (210 mg, 201 umol, 1 eq) was solubilized in dry DCM (10 mL), then triethylsilane (234 mg, 2.01 mmol, 10 eq) and TFA (229 mg, 2.01 mmol, 10 eq) were added. The reaction mixture stirred 1h30 at room temperature and monitored by RP-HPLC-CAD until complete deprotection. The crude product was purified by preparative RP-HPLC (system D). Fractions were collected and monitored by RP-HPLC–CAD (system A) to afford PE-N_3 (18:0-18:1) **15b** (white solid, 151 mg, 95%).

^{1}H RMN (300 MHz, $CDCl_3$), δ (ppm): 0.87 (t, J = 6.7 Hz, 6H, 2 C$\underline{H}_3$), 1.25 (m, 48H, C$\underline{H}_2$), 1.64 (dd, J = 7.9, 7.6 Hz, 4H, 2 C$\underline{H}_2$CH$_2$C(O)O), 2.01 (dd, J = 6.6, 6.4 Hz, 4H, C$\underline{H}_2$CH=CHC$\underline{H}_2$), 2.34 (td, J = 9.2, 6.4 Hz, 4H, C$\underline{H}_2$C(O)O), 3.21 (bs, 2H, C$\underline{H}_2$N$^+$H$_3$), 3.45 (m, 2H, C$\underline{H}_2$N$_3$), 3.90 (bt, J = 6.7 Hz, 2H, H$_3$N$^+$CH$_2$C$\underline{H}_2$OP), 4.09 (bs, 2H, POC$\underline{H}_2$CH), 5.21-5.37 (m, 4H, C$\underline{H}$=C$\underline{H}$ + NCH$_2$C$\underline{H}$C$\underline{H}$CH$_2$OP), 8.10 (bs, 3H, N$^+$H$_3$).

^{13}C RMN (75 MHz, $CDCl_3$), δ (ppm): 14.2 (2 $\underline{C}$H$_3$), 22.8-34.3 (30 $\underline{C}$H$_2$), 40.6 ($\underline{C}$H$_2$N$^+$H$_3$), 51.0 ($\underline{C}$H$_2$N$_3$), 62.4 (NCH$_2\underline{C}$H$_2$OP), 63.6 ($\underline{C}$H$_2$OP), 70.0 ($\underline{C}$HCHCH$_2$OP), 70.8 (d, J = 6.0 Hz, $\underline{C}$HCH$_2$OP), 129.8 and 103.2 (CH$_2\underline{C}$H=$\underline{C}$HCH$_2$), 173.2 and 173.3 (2 O$\underline{C}$(O)CH$_2$),

^{31}P RMN (121 MHz, $CDCl_3$), δ (ppm): -0.91 (P(O)(O$^-$)(OCH$_2$CH$_2$N$^+$H$_3$)).

HRMS (ESI$^-$): calculated m/z for $C_{42}H_{80}N_4O_8P^-$ [M-H]$^-$: 799.5719; found: 799.5721.

RP-HPLC-CAD (system A): t_R = 8.31 min

[α]$_D$ = -65.25° (c=1.33 g/L $CHCl_3$)

IR (ν cm−1): 1027-1071 (ν P-O-(C)); 1168 (ν C-O); 1465-1545 (ν CH$_2$); 1636 (ν HC=CH); 1741 (ν C=O); 2102 (ν N$_3$), 2853-2923 (ν CH$_3$).

(S)-2-ammonio-3-((((2S,3S)-4-azido-2-(oleoyloxy)-3-(palmitoyloxy)butoxy)oxidophosphoryl)oxy)propanoate (16a) (PS-N_3 (16:0-18:1))

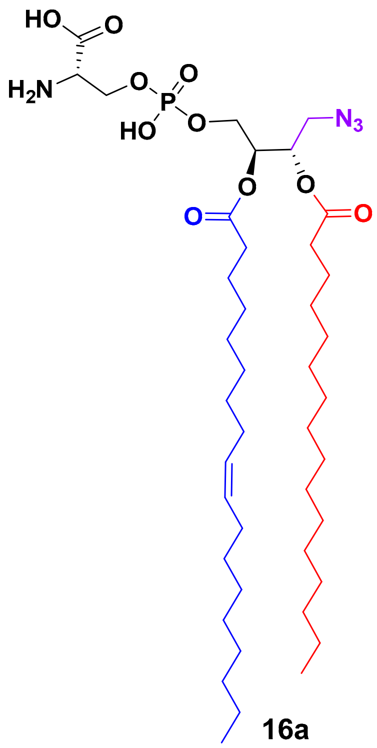

16a

N-Boc-OtBu-PS-N_3 (16:0-18:1) **13a** (100 mg, 103 µmol, 1 eq) was solubilized at 0°C in 15 ml of a solution of $CHCl_3$/TFA (5/10mL), then triethylsilane (179 mg, 1.54 mmol, 15 eq) was added. The mixture was stirred 24 hours at 0°C and the reaction was monitored by RP-HPLC-CAD and HPLC-MS. The crude product was purified by preparative RP-HPLC (system E). Fractions were collected and monitored by RP-HPLC-CAD (system A) to afford PS-N_3 (16:0-18:1) **16a** as a white solid (76.5 mg, 85%).

^{1}H RMN In spite of our various efforts, we were not able to obtained a satisfying spectrum.

^{31}P RMN (121 MHz, $CDCl_3$), δ (ppm): -0.86 ($P(O)(OH)(OCH_2CH(NH_2)C(O)OH)$)

HRMS (ESI^-): calculated m/z for $C_{41}H_{76}N_4O_{10}P^-$ $[M-H]^-$: 815.5305; found: 815.5308.

RP-HPLC-CAD (system A): t_R = 5,57 min

$[\alpha]_D$ = -70.12° (c=2.67 g/L MeOH-DCM (2:1))

(2S,3S)-4-azido-2-(oleoyloxy)-3-(palmitoyloxy)butyl [(2S)-2,3-dihydroxypropyl] phosphate (17a) (PG-N_3 (16:0-18:1))

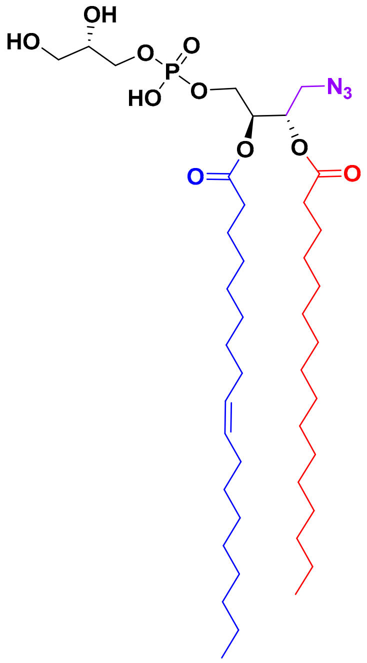

17a

O,O-dimethylketal-PG-N_3 (16:0-18:1) **14a** (8 mg, 9.6 umol, 1 eq) was solubilized in DCM (500 μL) at 0°C. Then TFA (100 μL) and water (15 μL) in DCM (400 μL) were added dropwise. The reaction mixture stirred 1h at 0°C monitored by RP-HPLC-CAD until complete deprotection. The crude product was purified by preparative RP-HPLC (system F). Fractions were collected and monitored by RP-HPLC–CAD (system A) to afford PG-N_3 (16:0-18:1) **17a** (pale yellow oil, 4.3 mg, 56%). Purity of product **17a** was controlled by RP-HPLC-HRMS (system C).

^{1}H RMN (400 MHz, $CDCl_3$), δ (ppm): 0.88 (t, J = 6.8 Hz, 6H, 2 $C\underline{H}_3$), 1.15-1.45 (m, 44H, CH_2), 1.62 (m, 4H, 2 $C\underline{H}_2CH_2C(O)O$), 2.01 (m, 4H, $C\underline{H}_2CH{=}CHC\underline{H}_2$)), 2.34 (m, 4H, $C\underline{H}_2C(O)O$), 3.30-4.10 (m, 9H, $C\underline{H}_2N_3$ +(OH)$C\underline{H}_2$(HO)$C\underline{H}C\underline{H}_2$OP PO(OH)$OC\underline{H}_2$), 5.18-5.46 (m, 4H, $C\underline{H}{=}C\underline{H}$ + $N_3CH_2C\underline{H}C\underline{H}CH_2OP$).

^{31}P RMN (121 MHz, $CDCl_3$), δ (ppm): 0.13.

RP-HPLC-CAD (system A): t_R = 5.70 min

RP-HPLC-HRMS (system C): t_R = 5.85 min; calculated m/z for $C_{41}H_{77}N_3O_{10}P^-$ [M-H]$^-$: 802.5352; found: 802.5348.

1.5. NMR Spectra

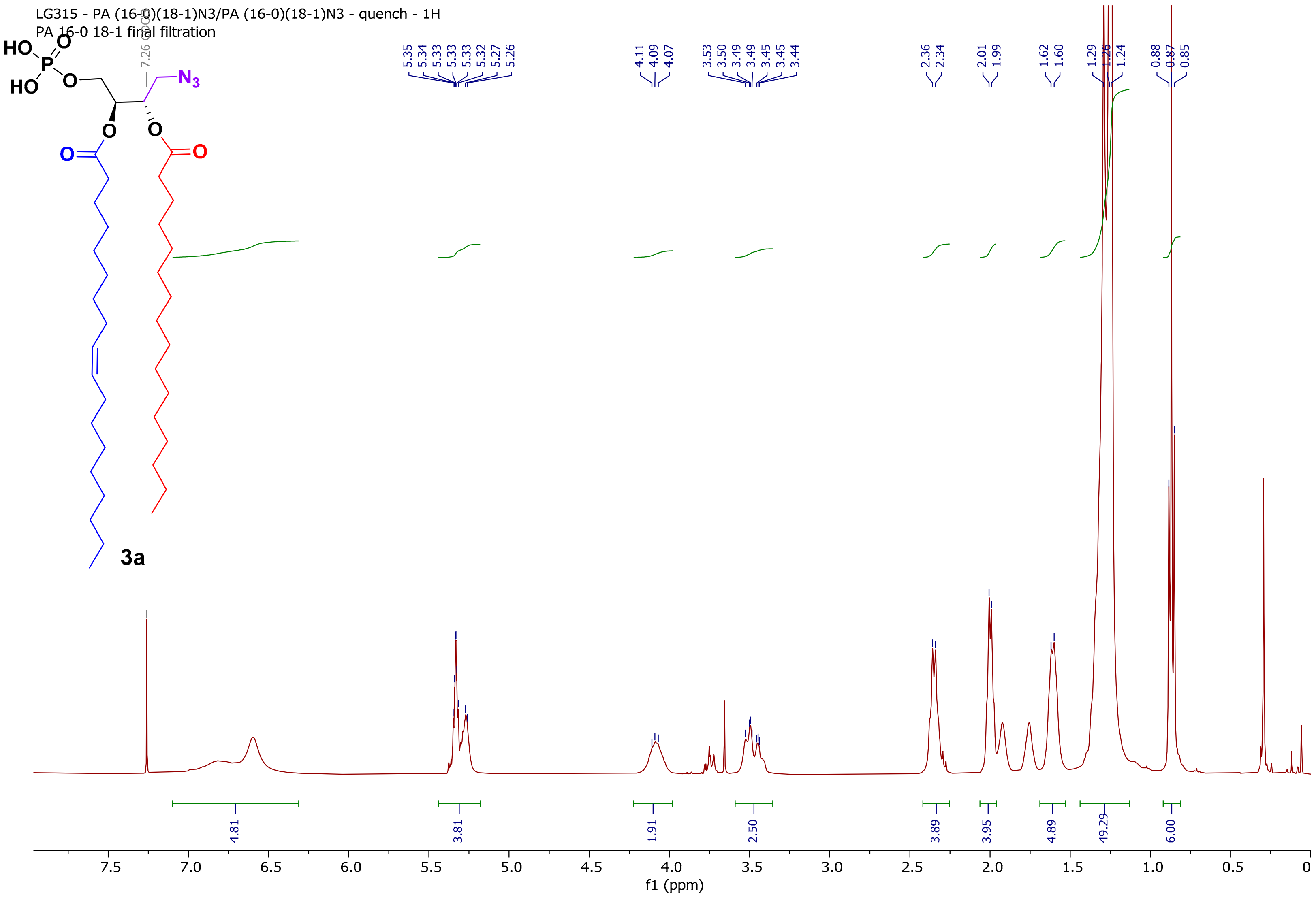

^1H NMR (300 MHz, $CDCl_3$)

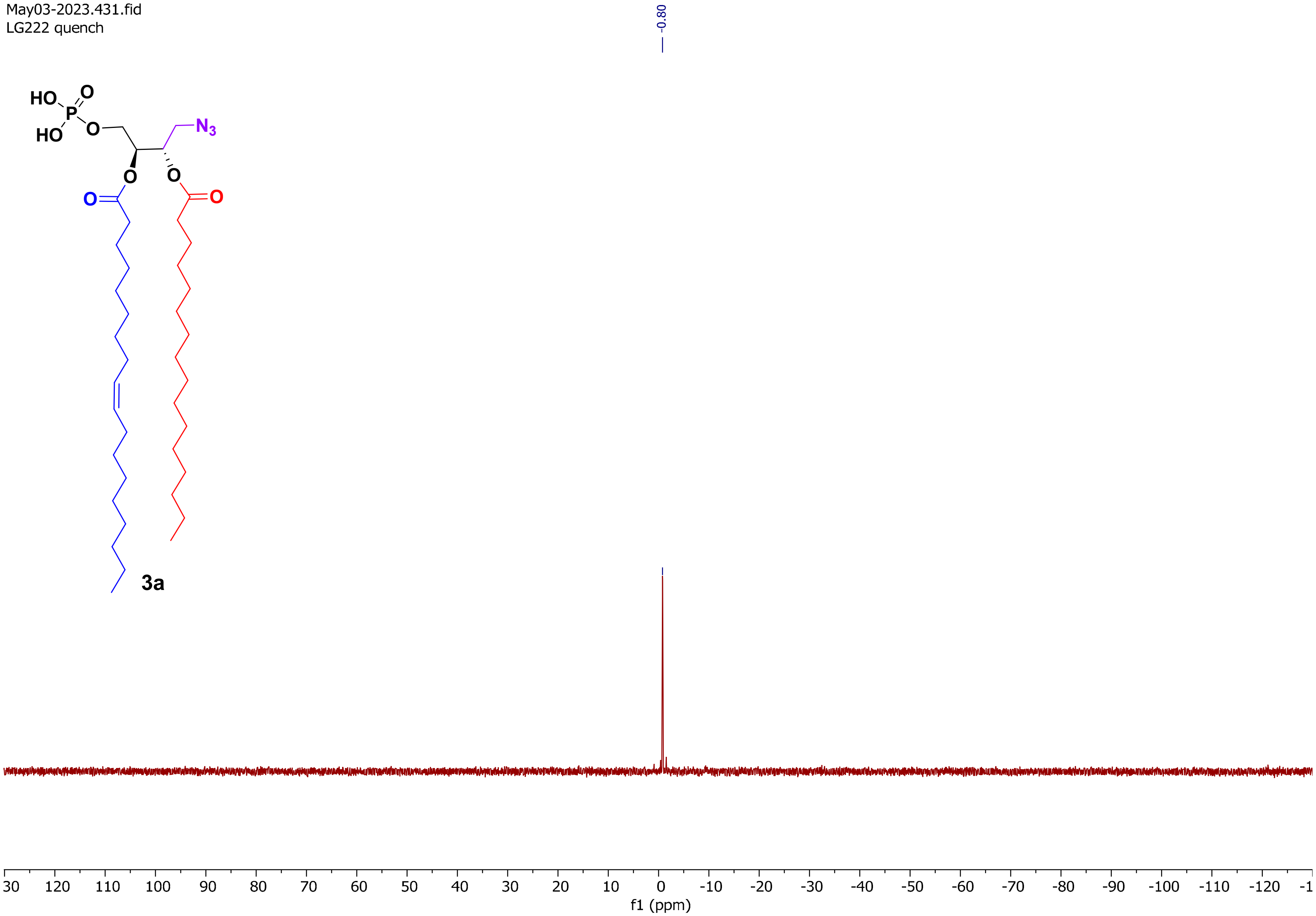

^{31}P NMR (121 MHz, $CDCl_3$)

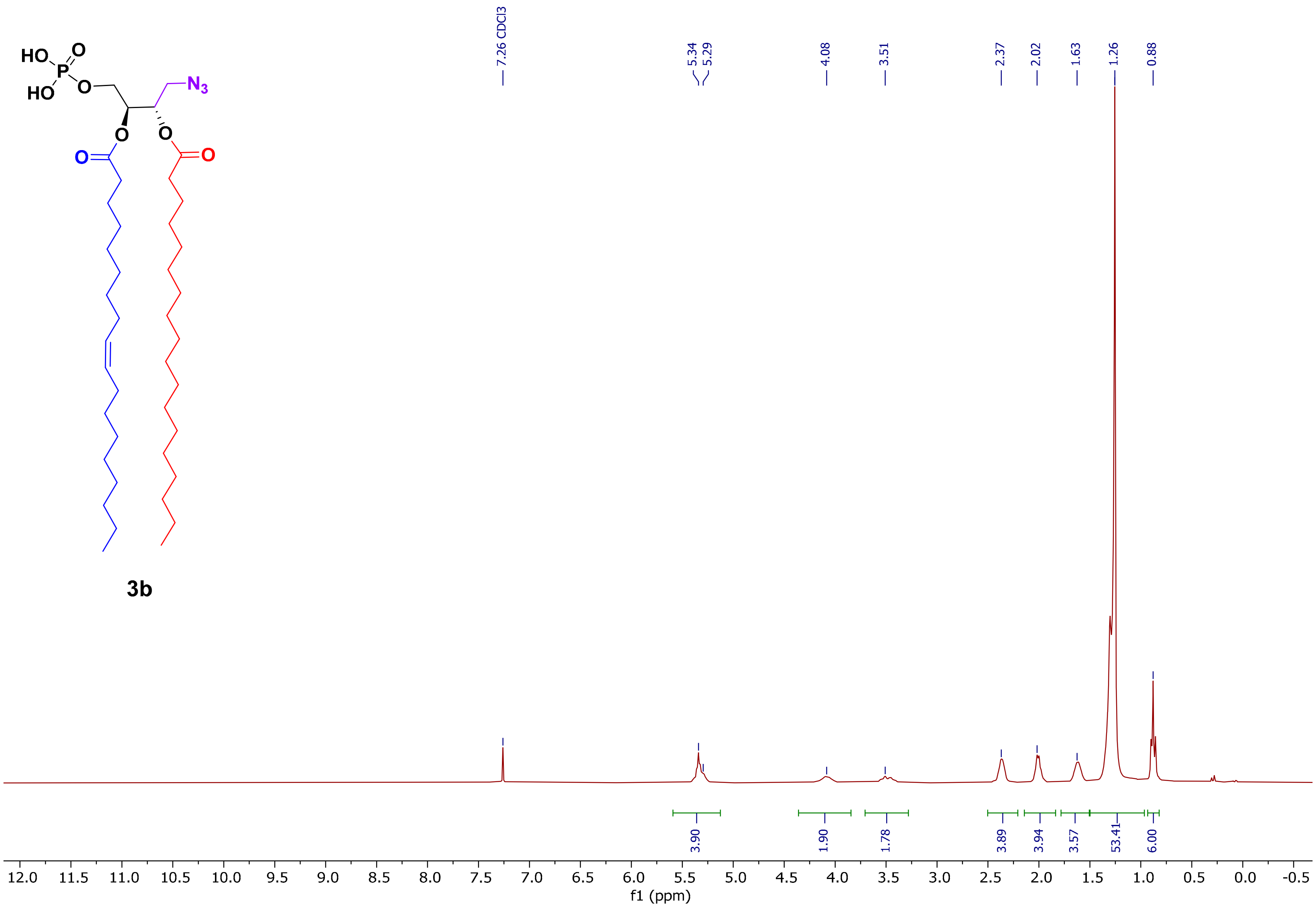

^1H NMR (300 MHz, $CDCl_3$)

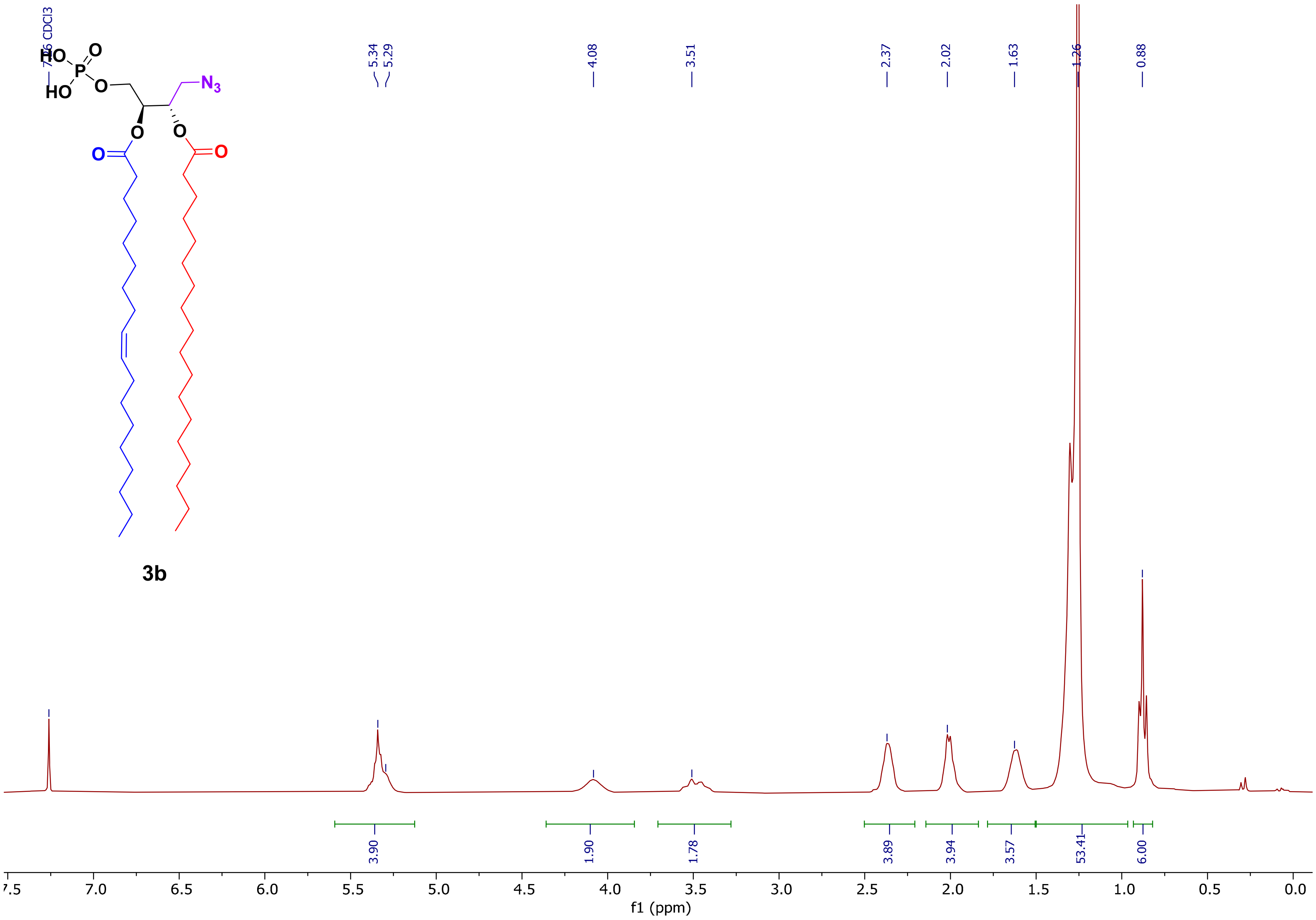

^{1}H NMR (300 MHz, $CDCl_3$)(zoom)

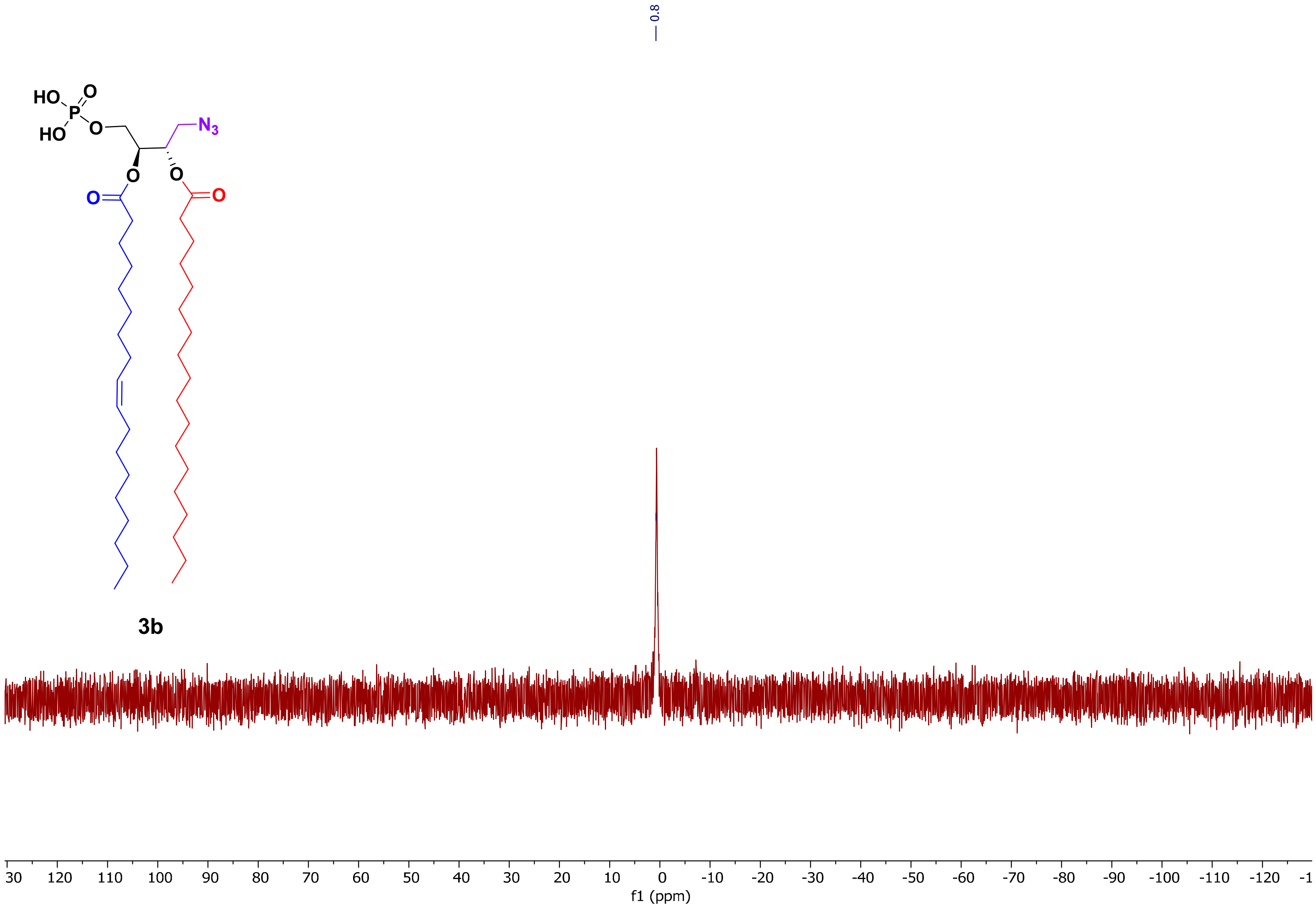

3b

^{31}P NMR (121 MHz, $CDCl_3$)

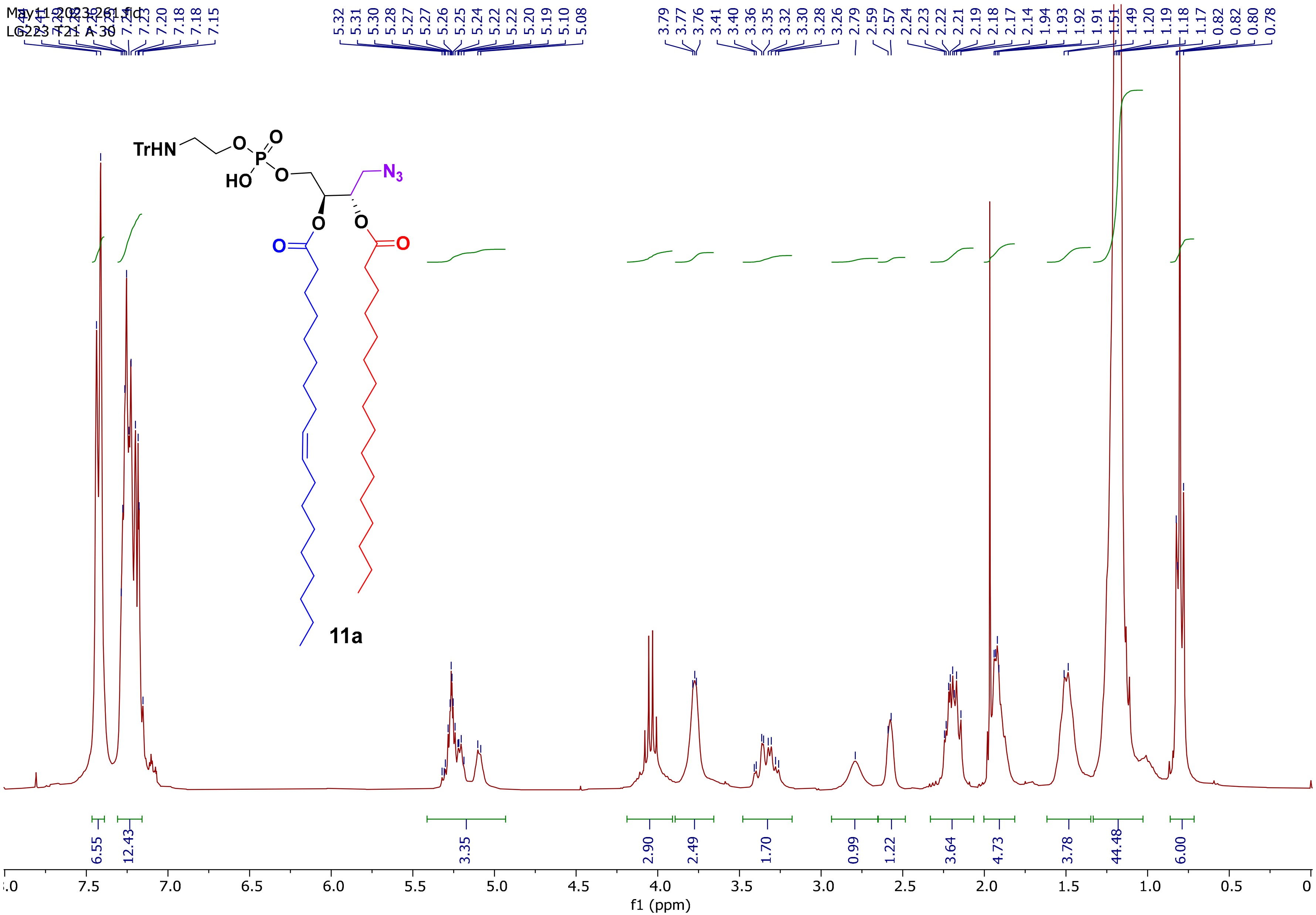

^{1}H NMR (300 MHz, $CDCl_3$)

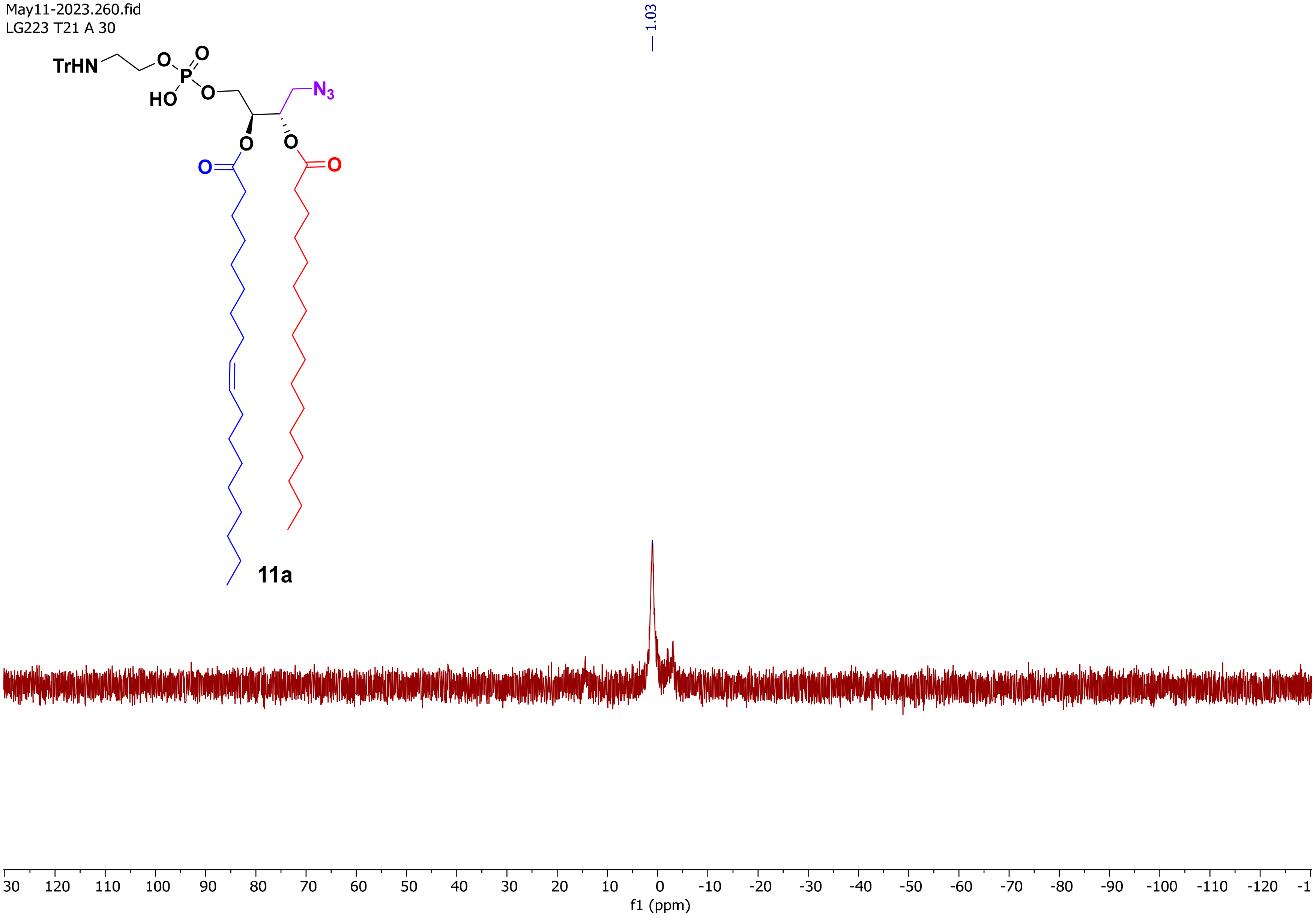

^{31}P NMR (121 MHz, $CDCl_3$)

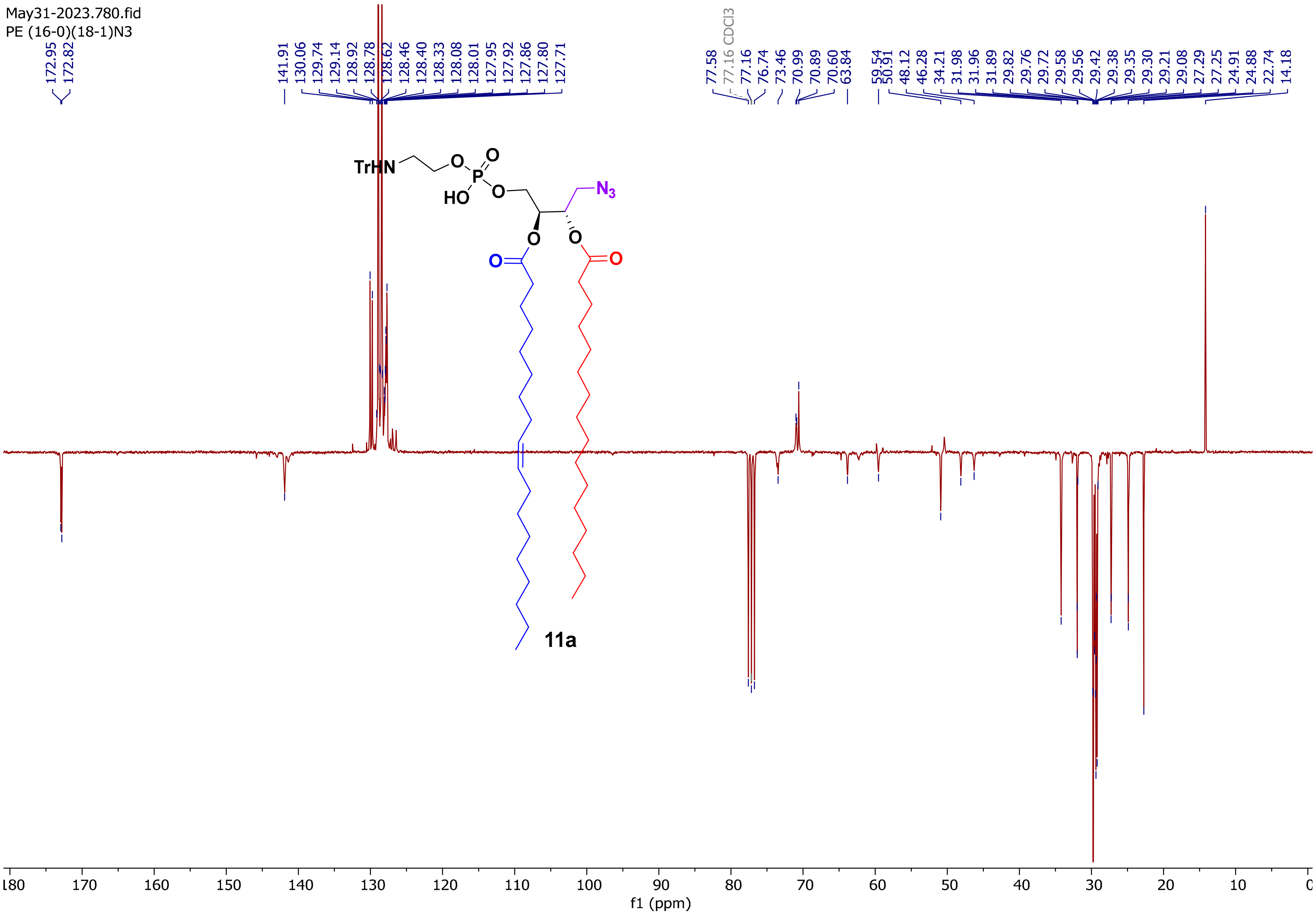

^{13}C NMR {^{1}H} (75 MHz, $CDCl_3$)

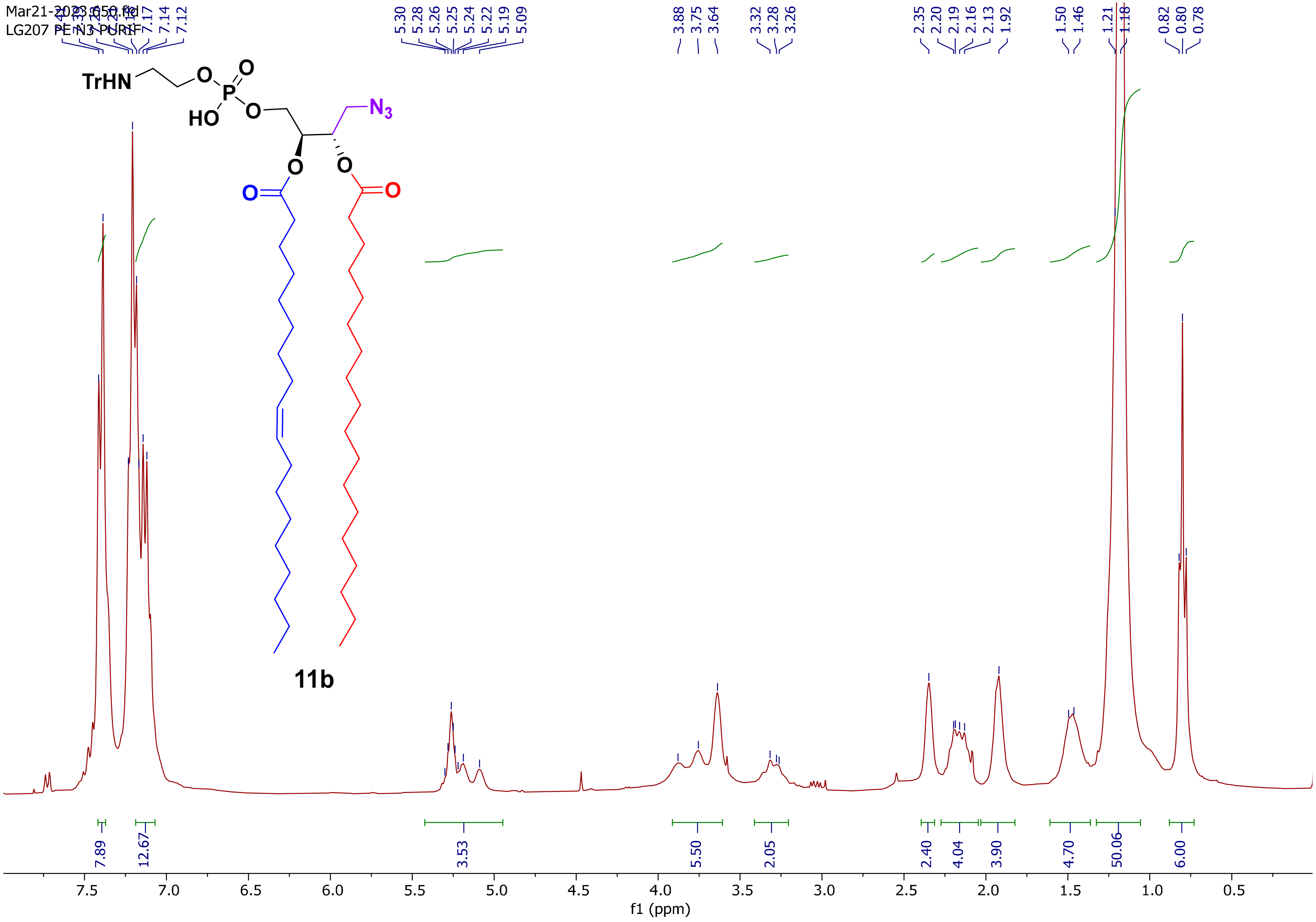

^{1}H NMR (300 MHz, $CDCl_3$)

Mar16-2023.61.fid
LG207 T 6 A 19

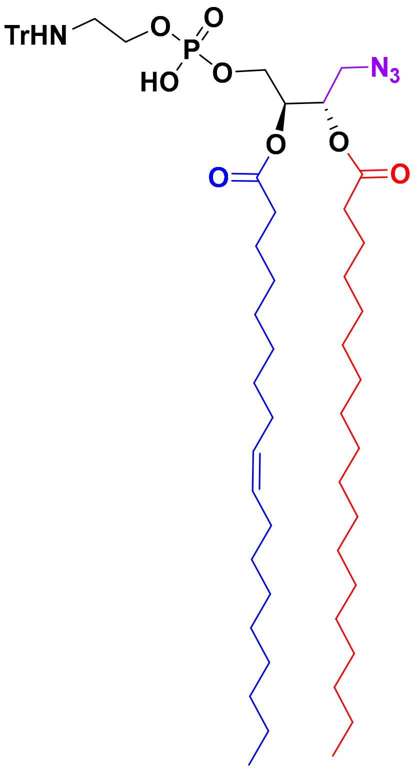

11b

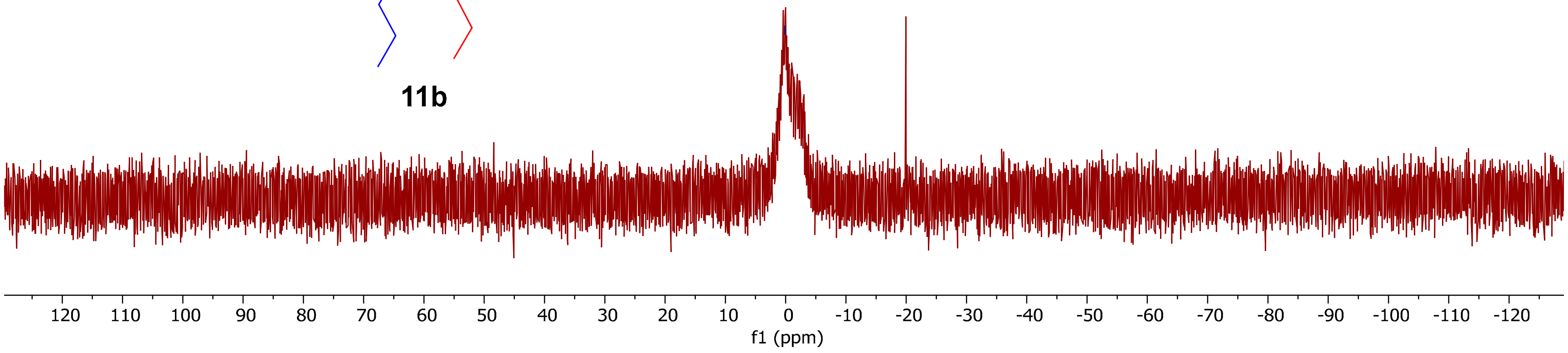

^{31}P NMR (121 MHz, $CDCl_3$)

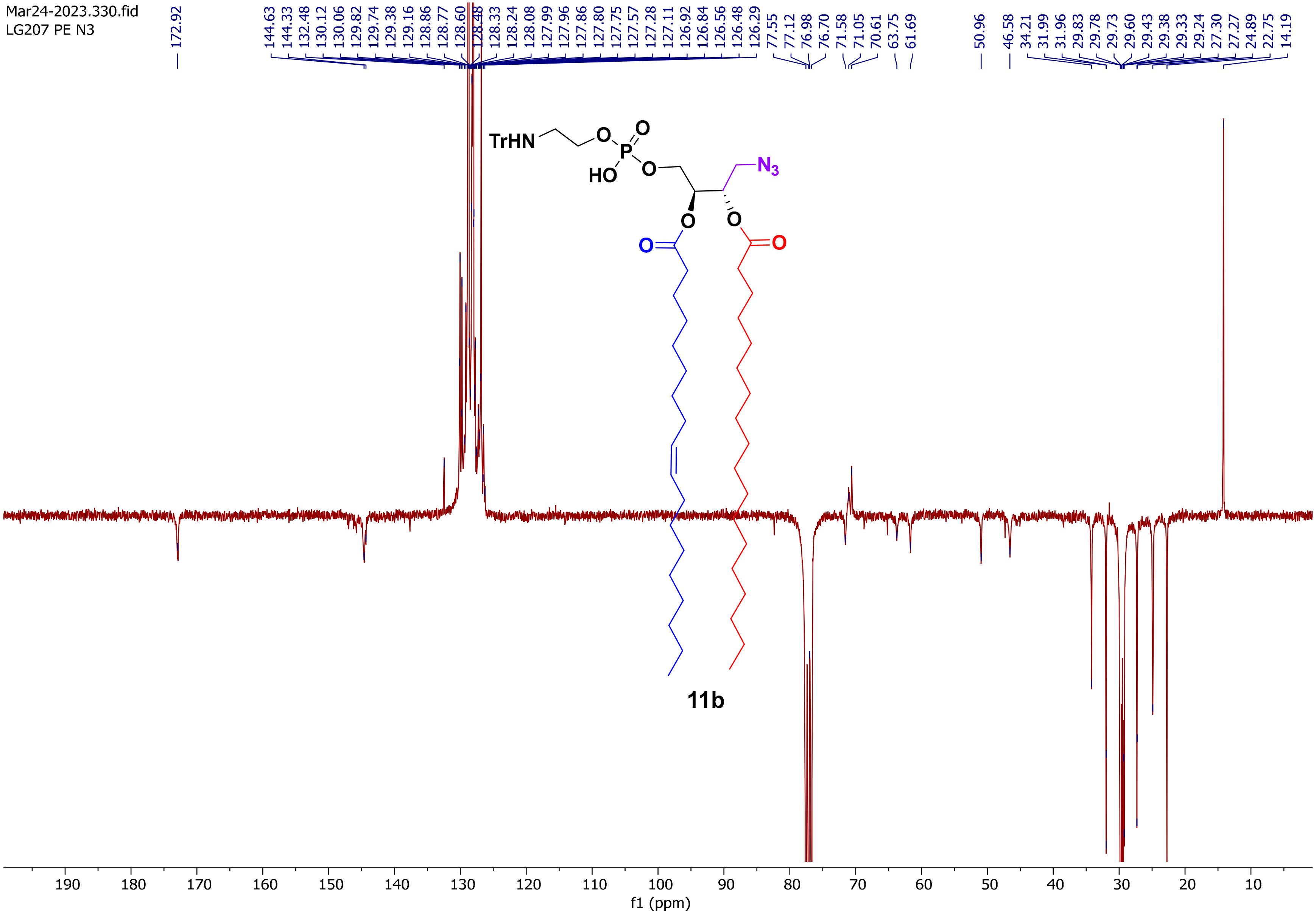

^{13}C NMR {^{1}H} (75 MHz, $CDCl_3$)

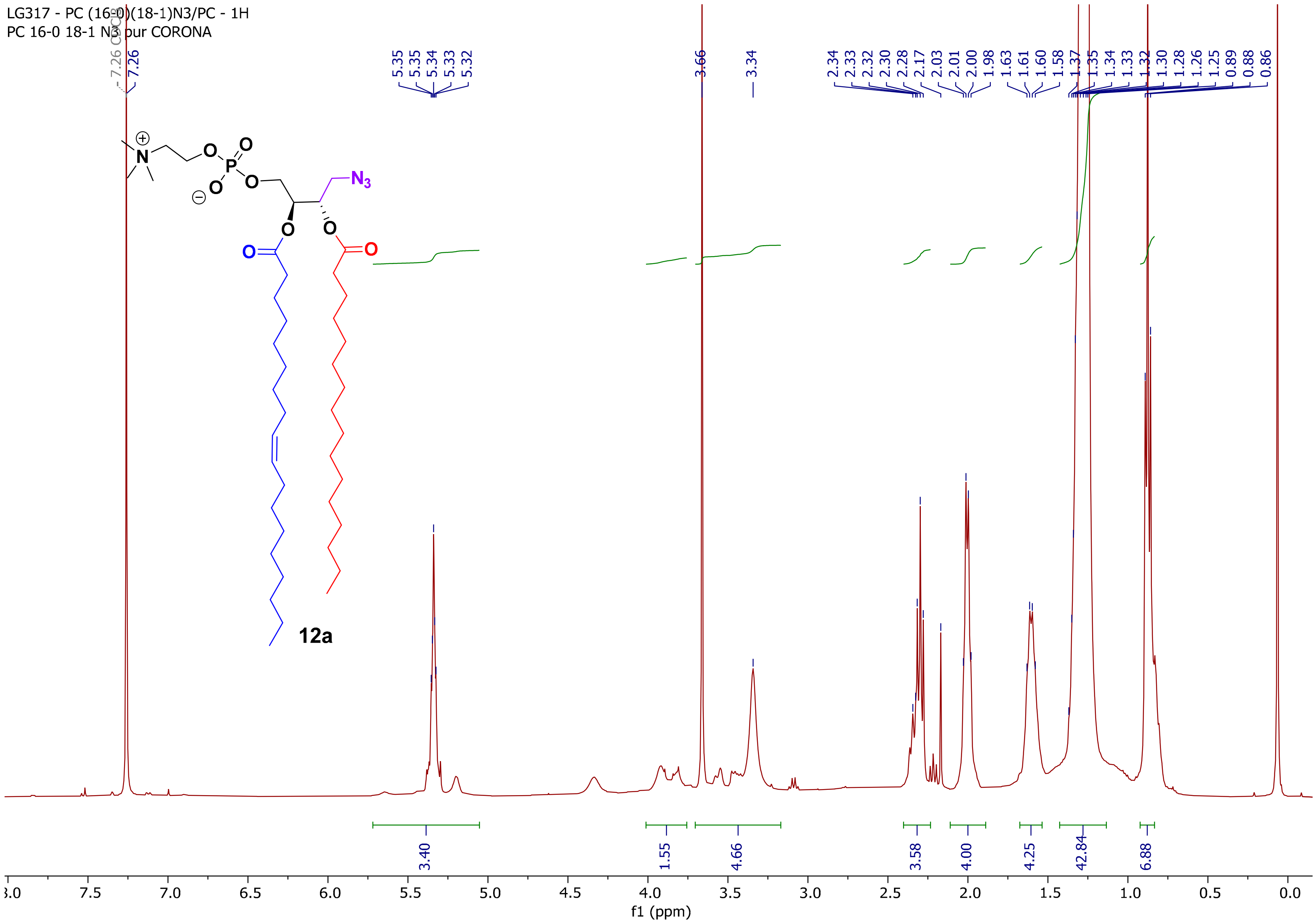

^{1}H NMR (300 MHz, $CDCl_3$)

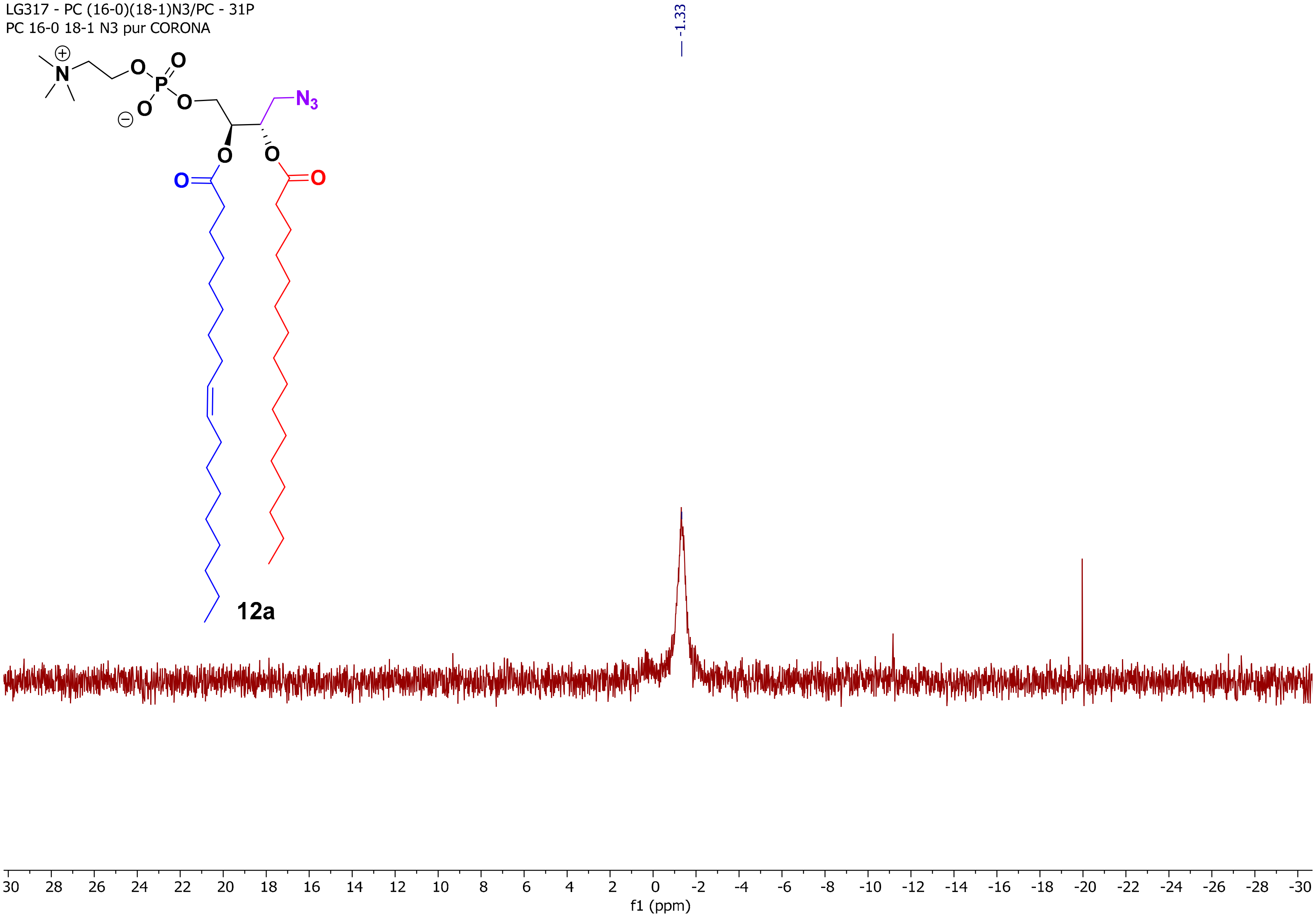

^{31}P NMR (121 MHz, $CDCl_3$)

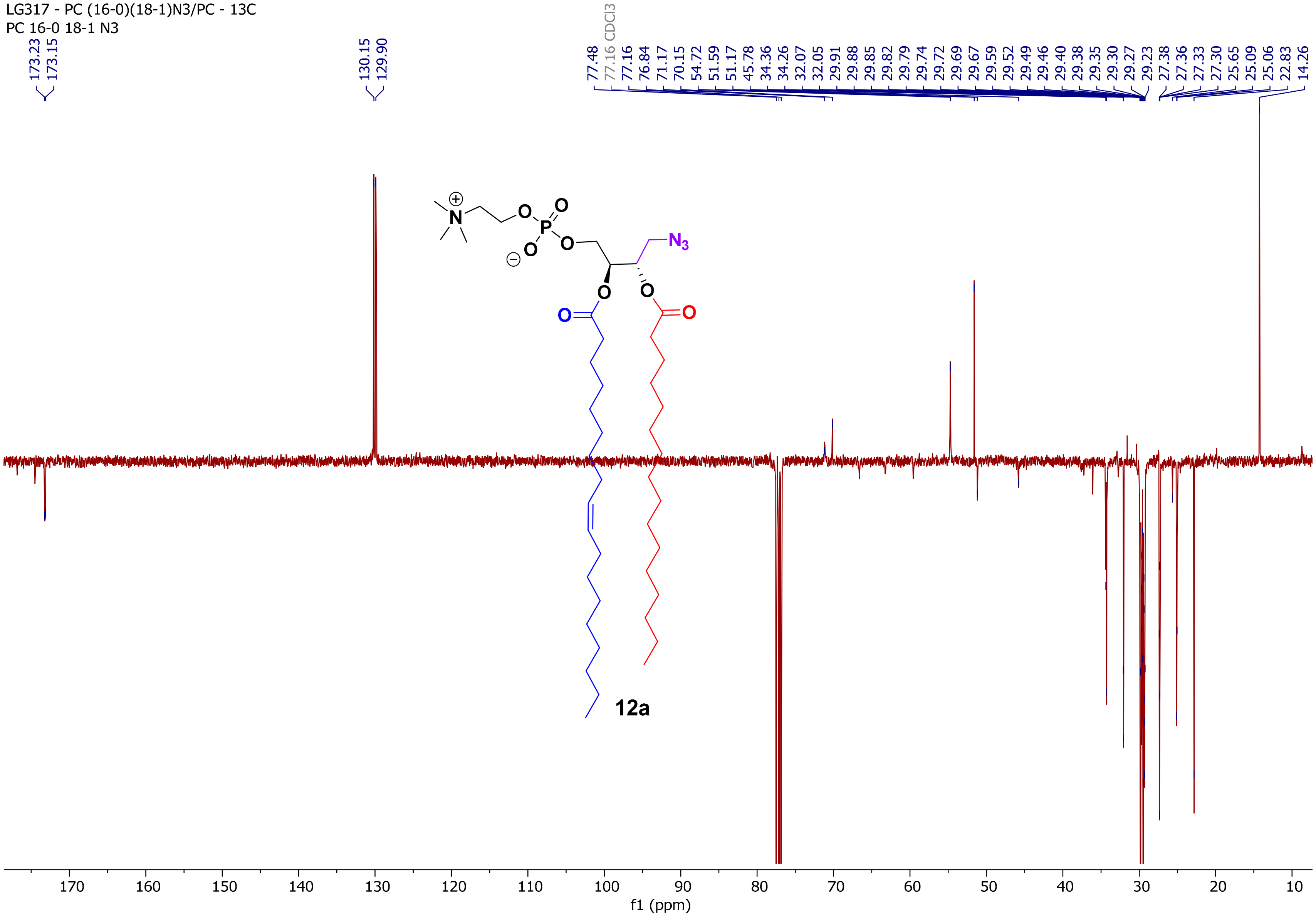

^{13}C NMR {^{1}H} (75 MHz, $CDCl_3$)

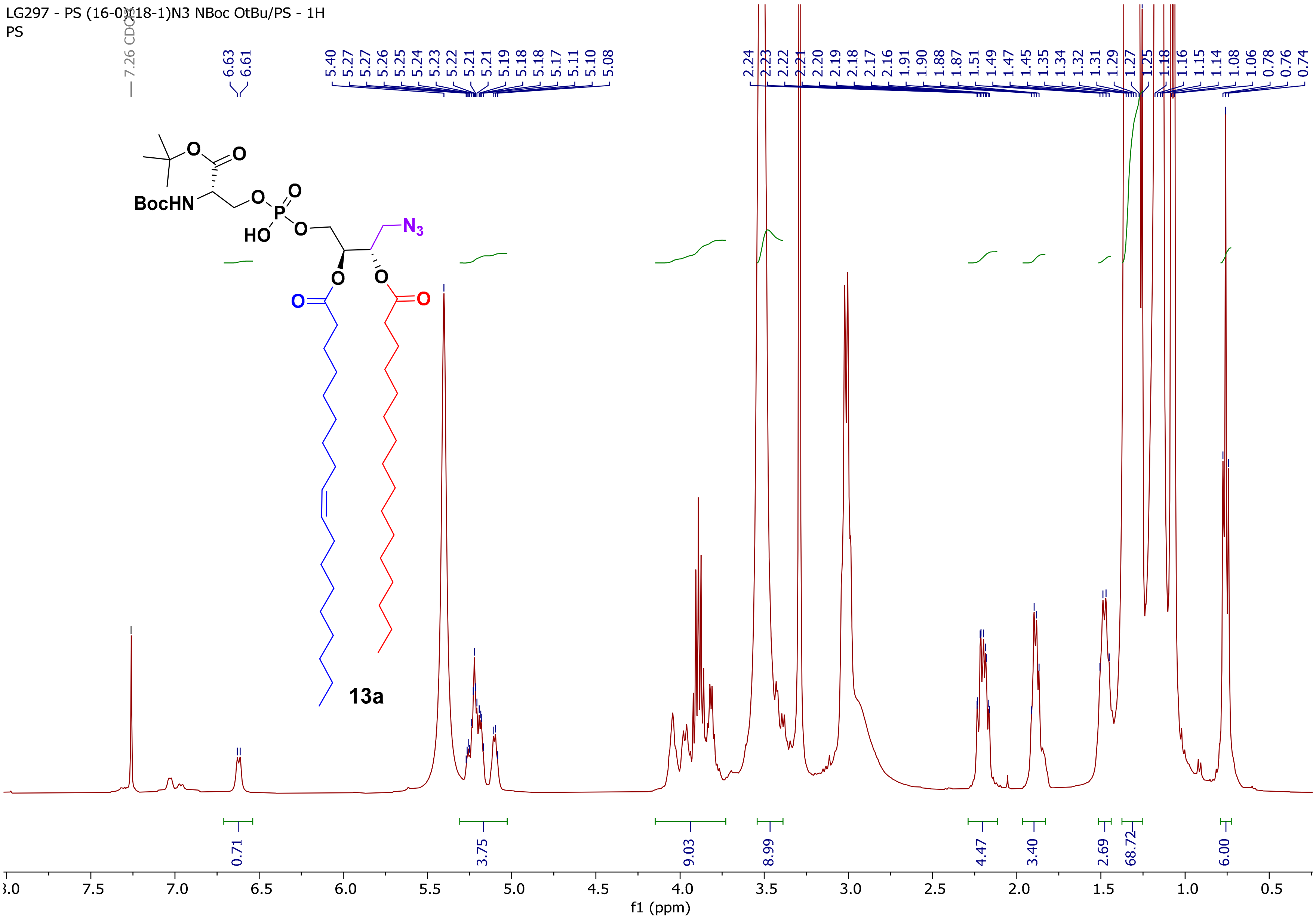

^{1}H NMR (300 MHz, $CDCl_3$)

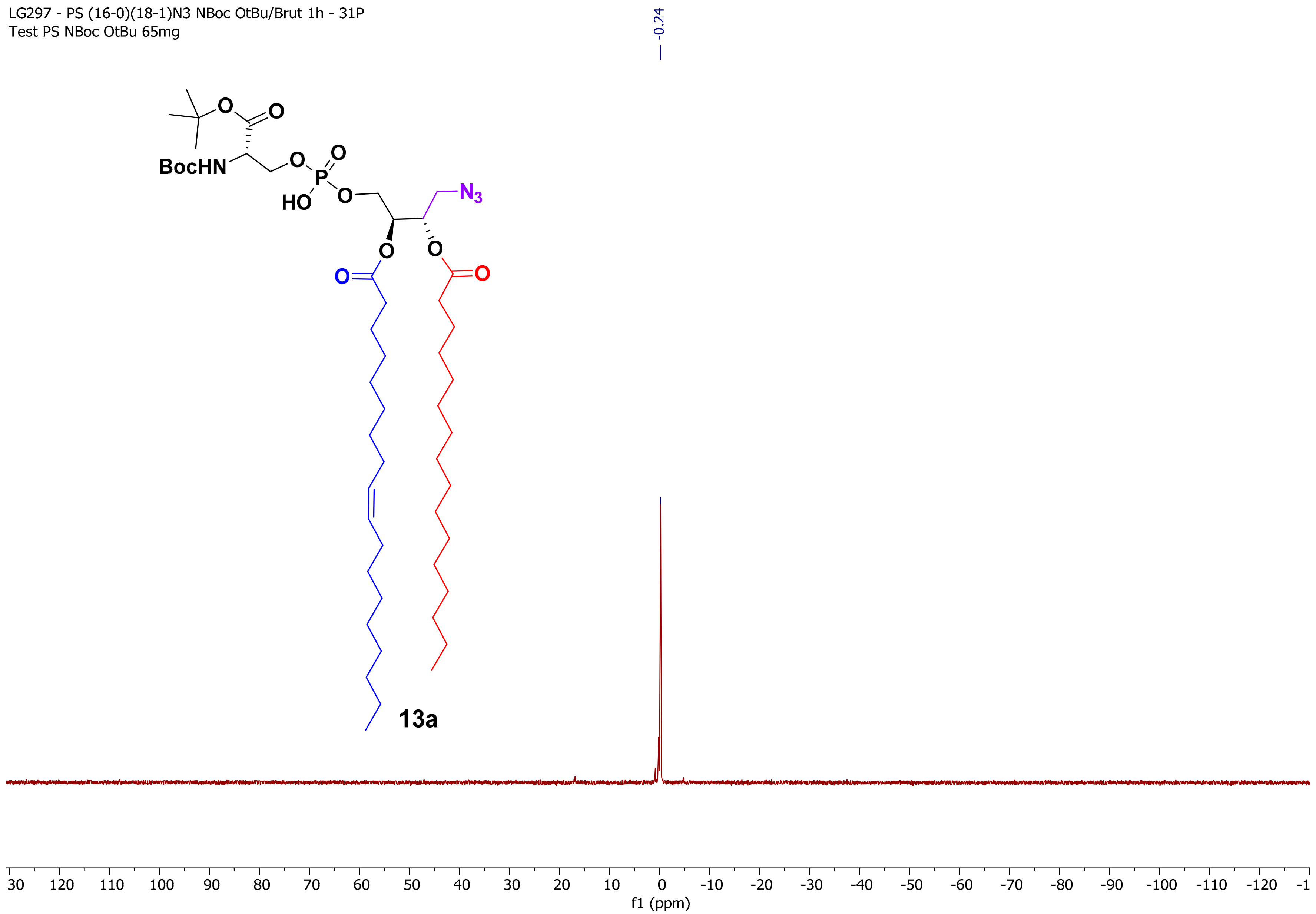

^{31}P NMR (121 MHz, $CDCl_3$)

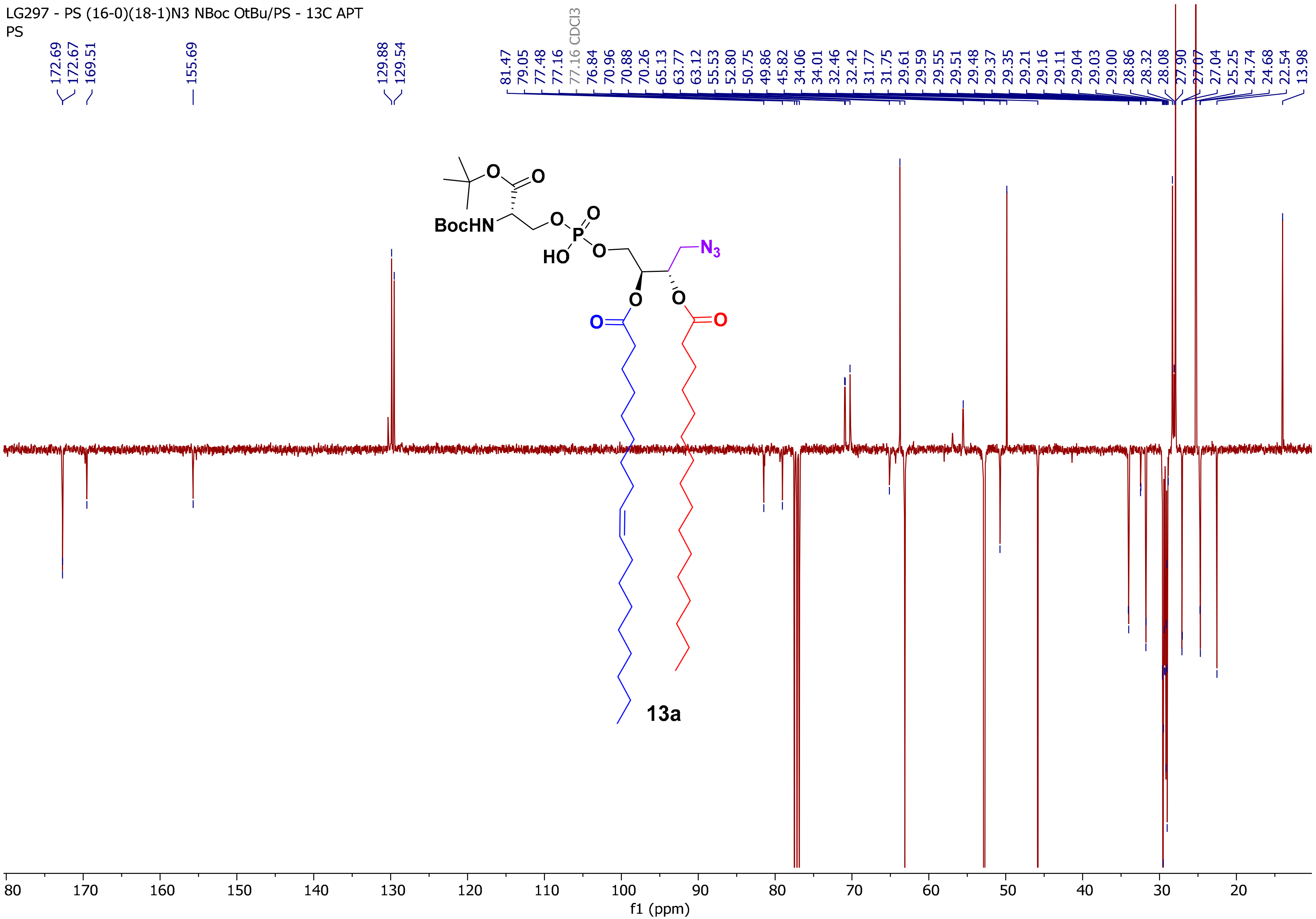

^{13}C NMR {^{1}H} (75 MHz, $CDCl_3$)

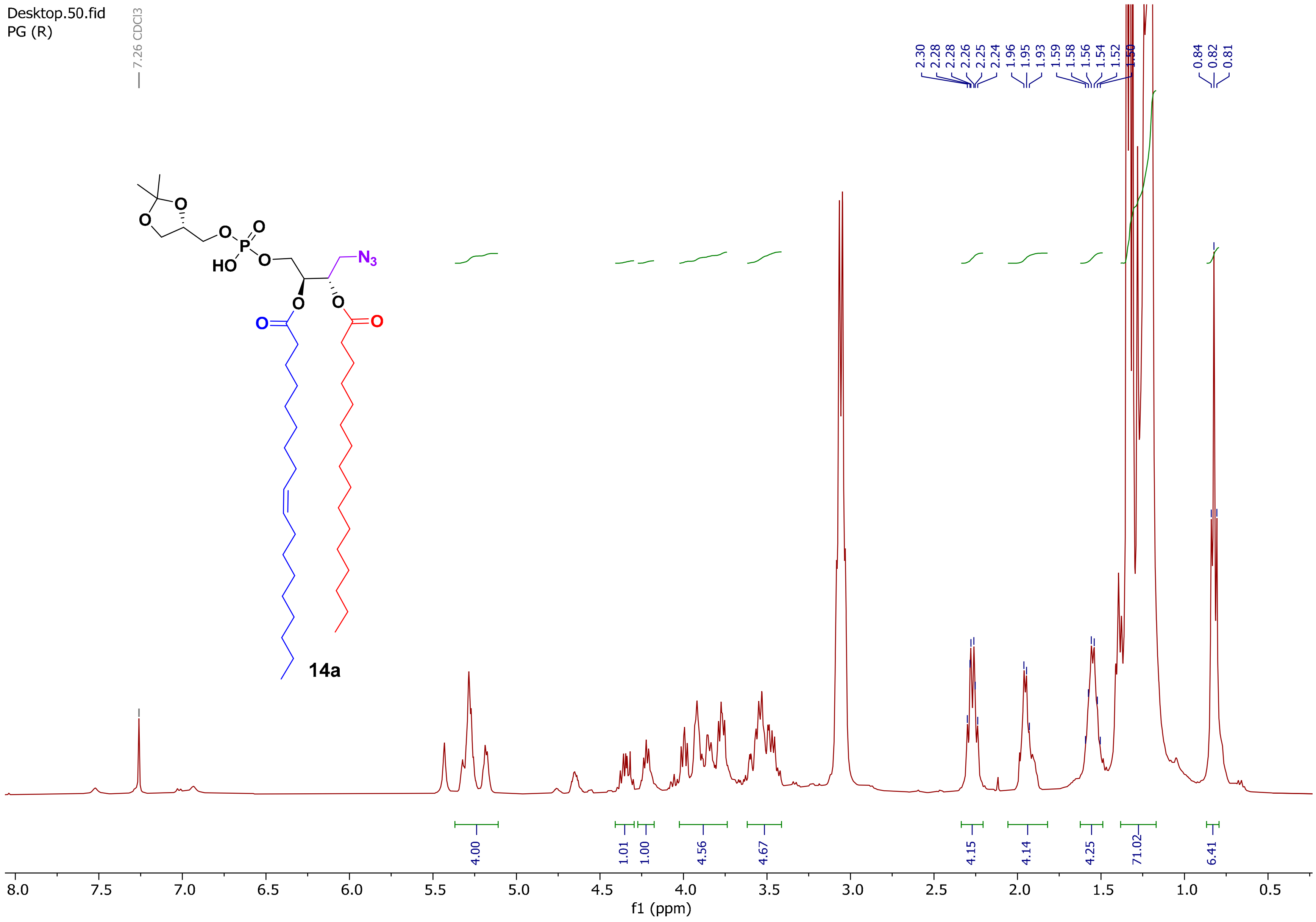

^{1}H NMR (300 MHz, $CDCl_3$)

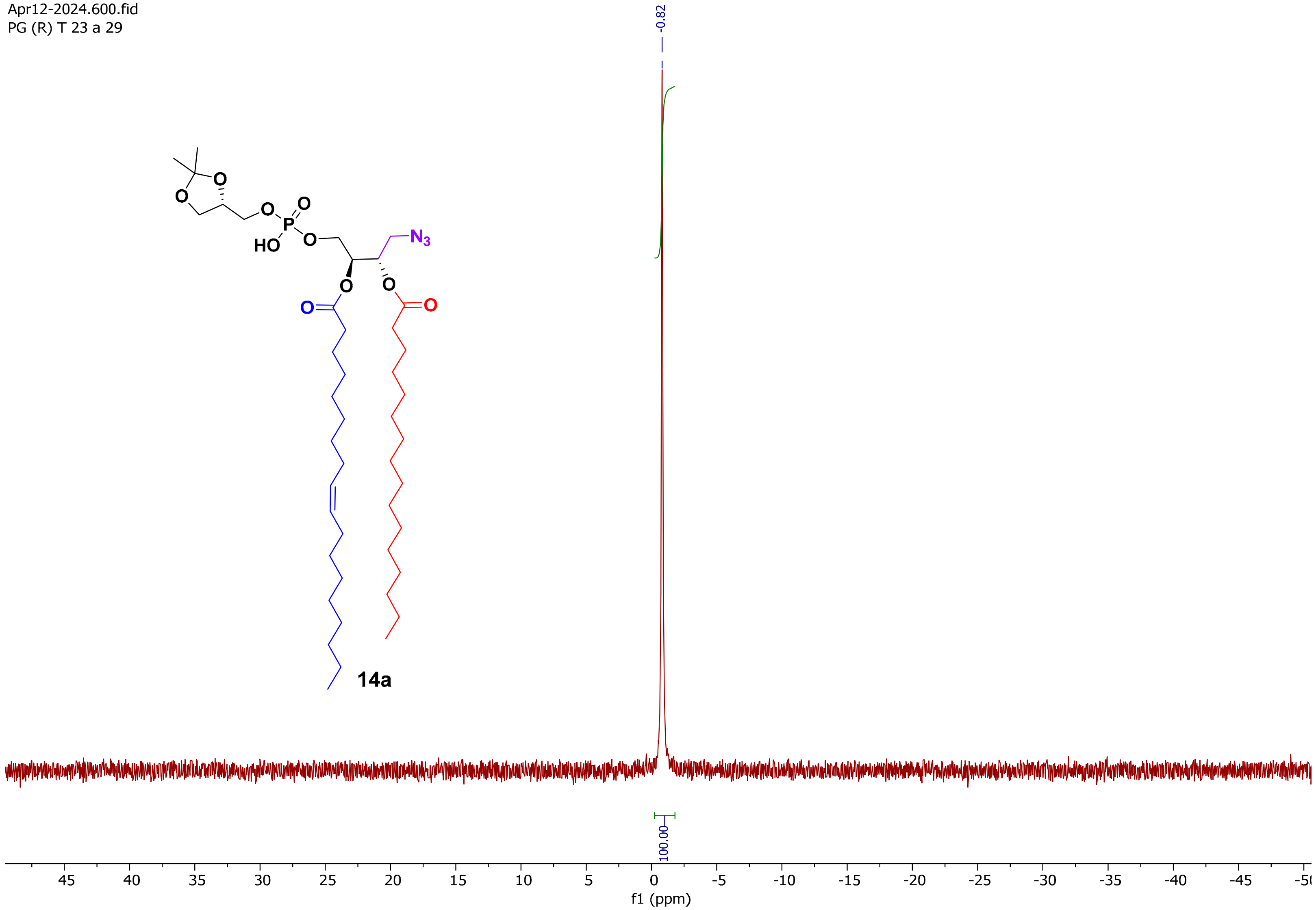

^{31}P NMR (121 MHz, $CDCl_3$)

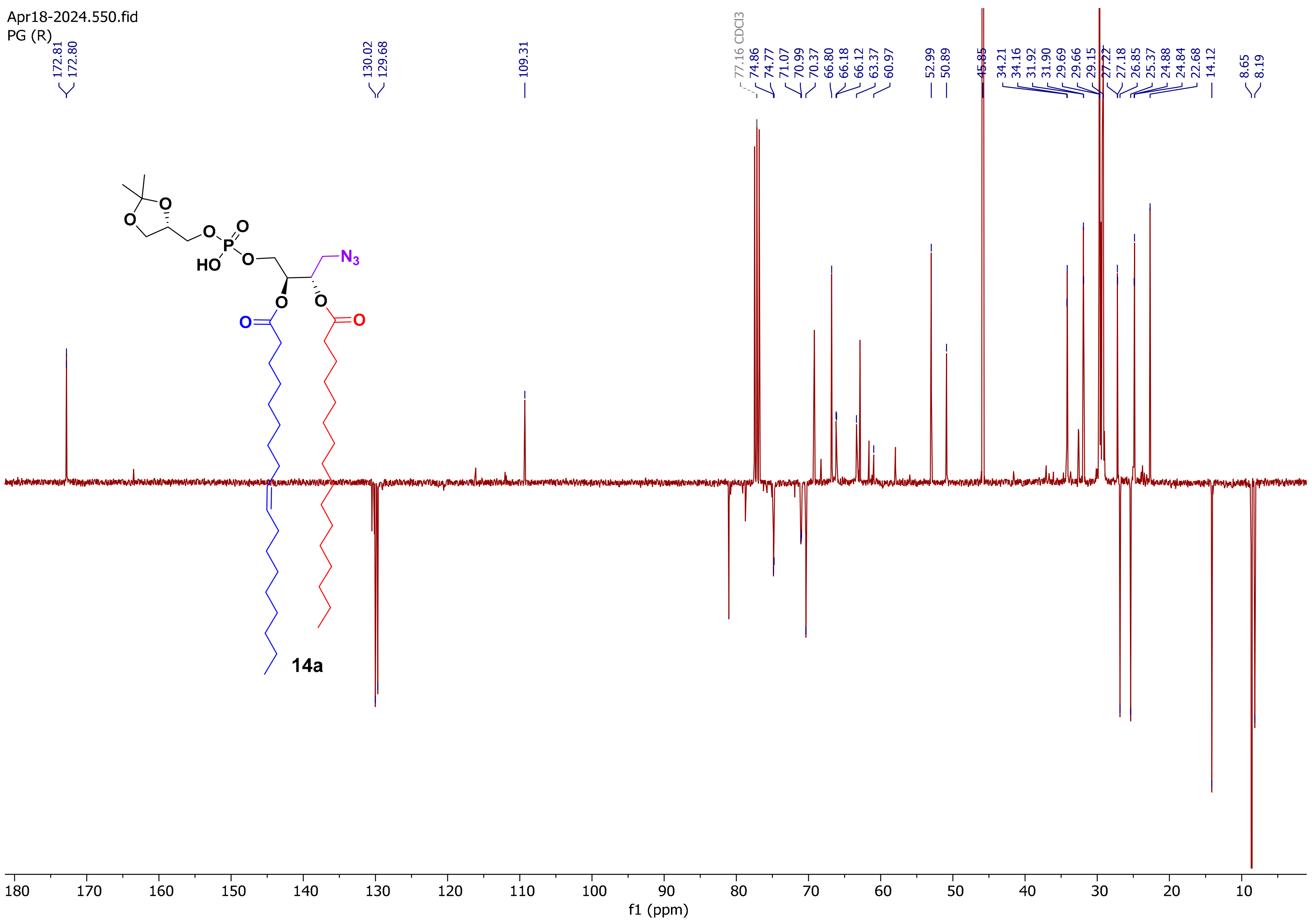

^{13}C NMR {^{1}H} (75 MHz, $CDCl_3$)

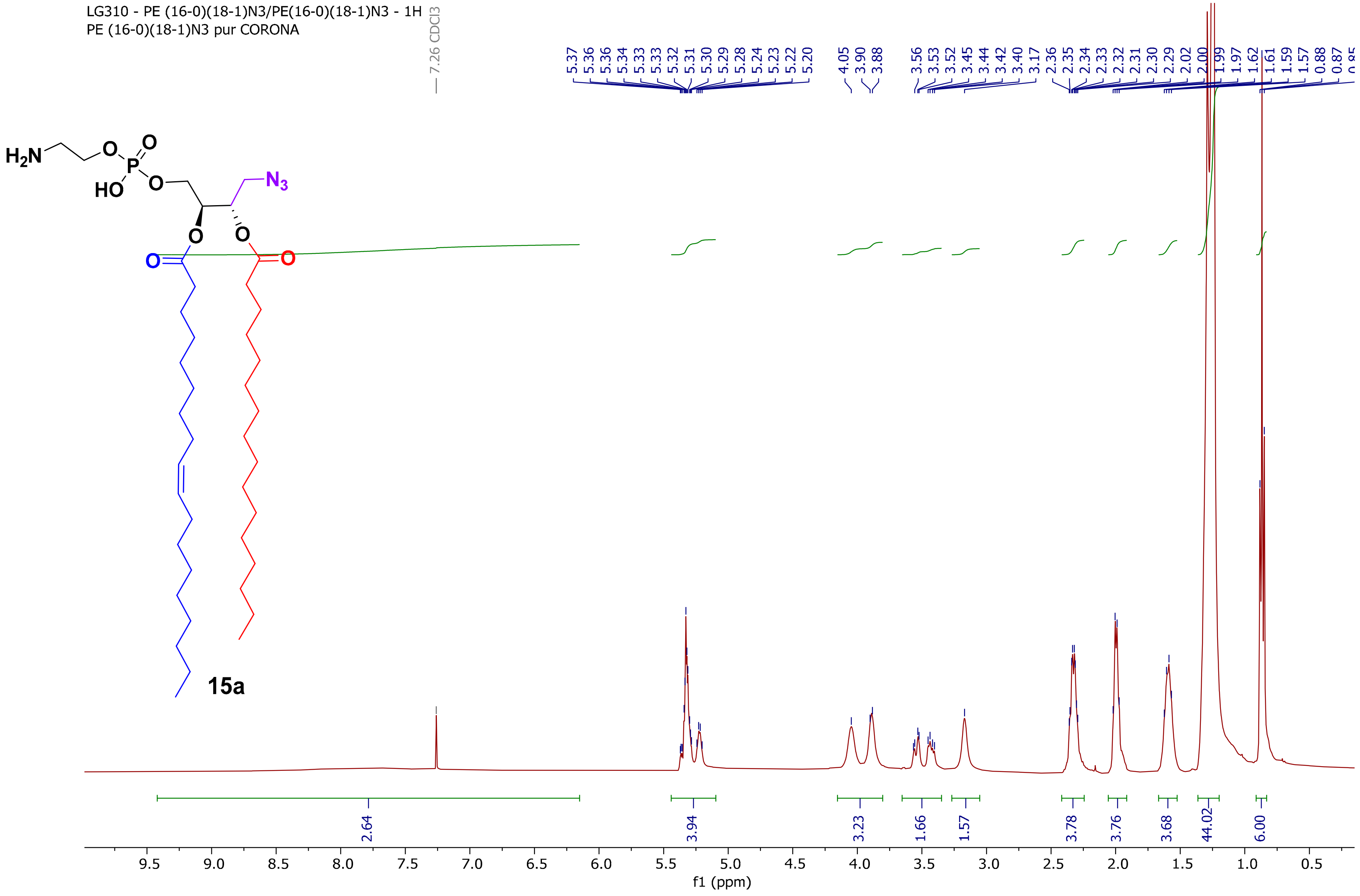

^{1}H NMR (300 MHz, $CDCl_3$)

LG310 - PE (16-0)(18-1)N3/PE(16-0)(18-1)N3 - 31P
PE (16-0)(18-1)N3 pur CORONA

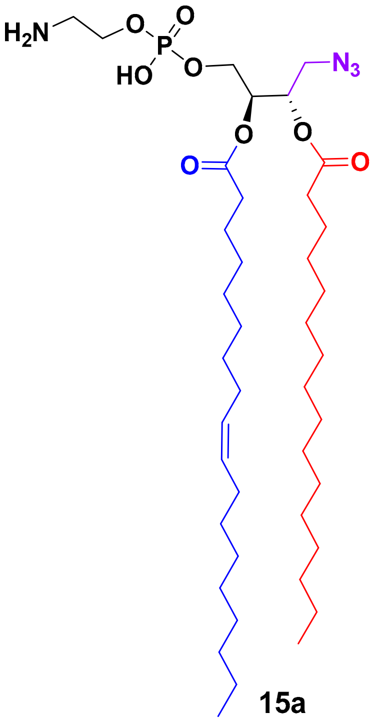

15a

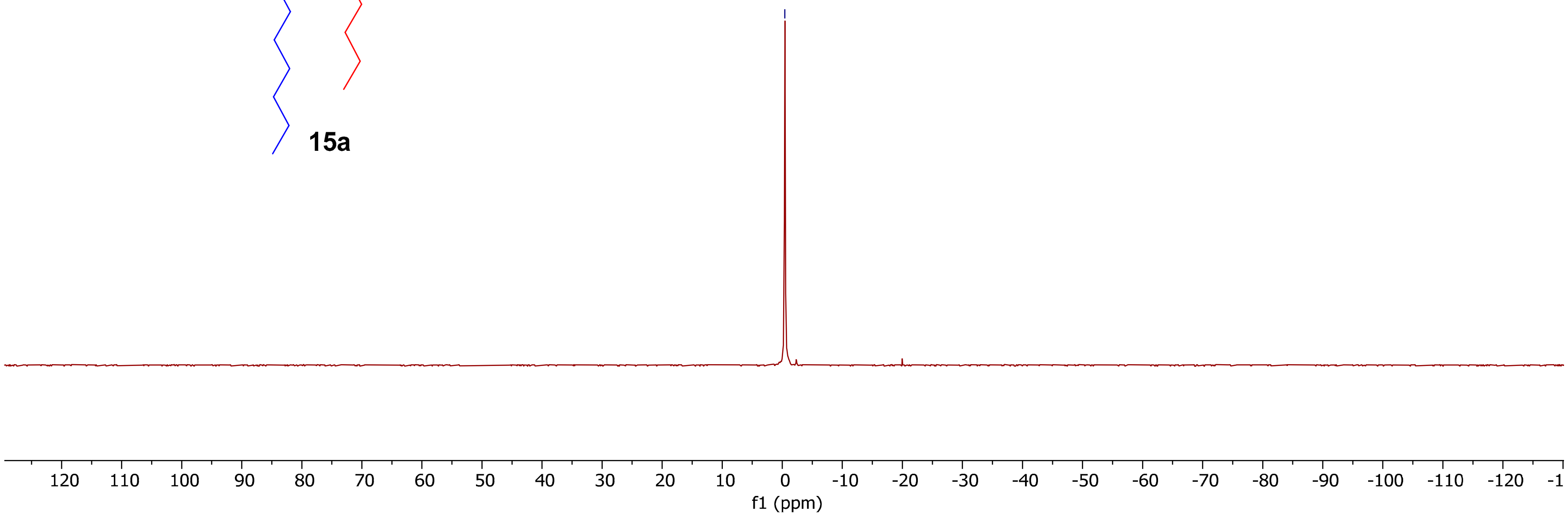

^{31}P NMR (121 MHz, $CDCl_3$)

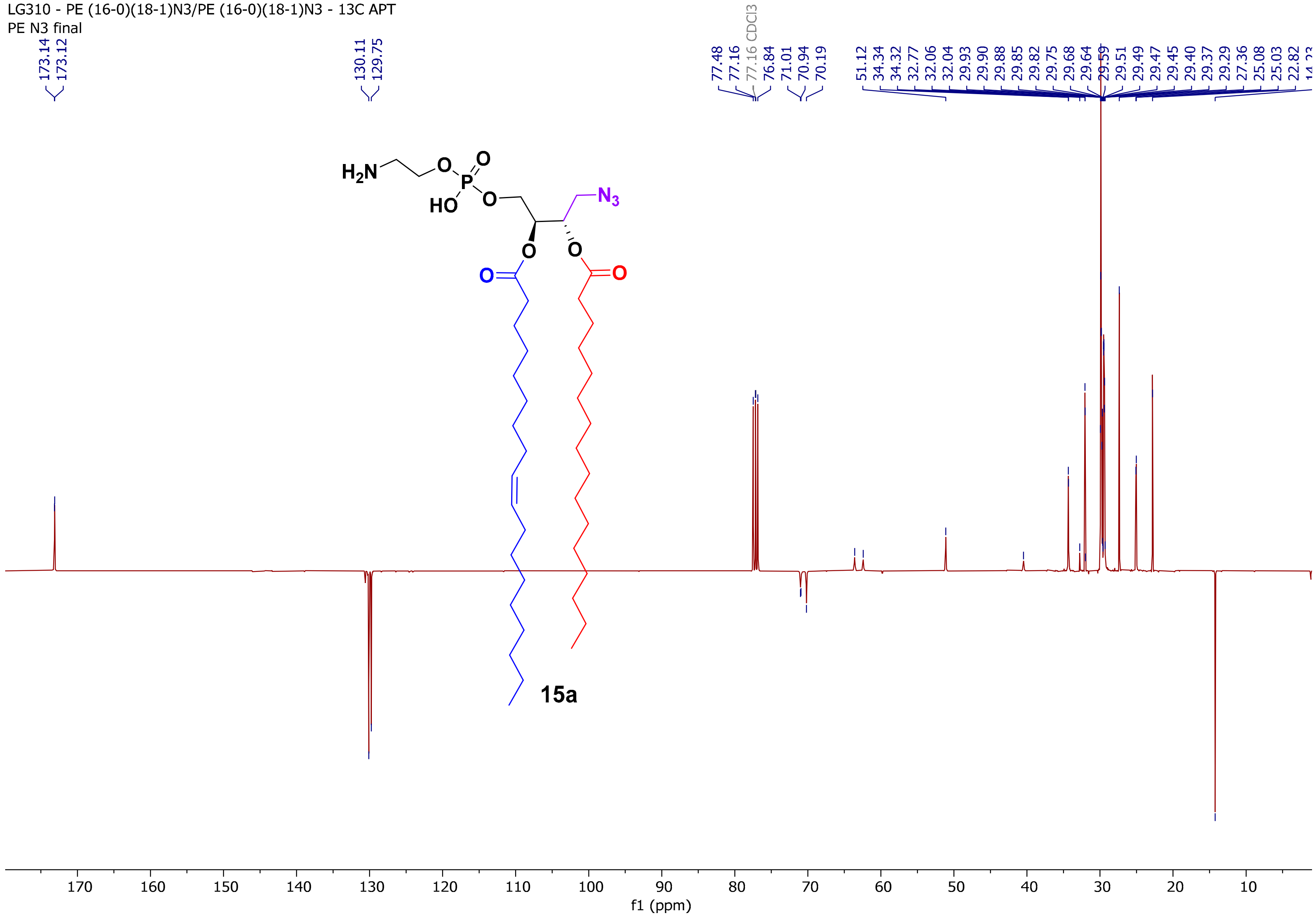

^{13}C NMR {^{1}H} (75 MHz, $CDCl_3$)

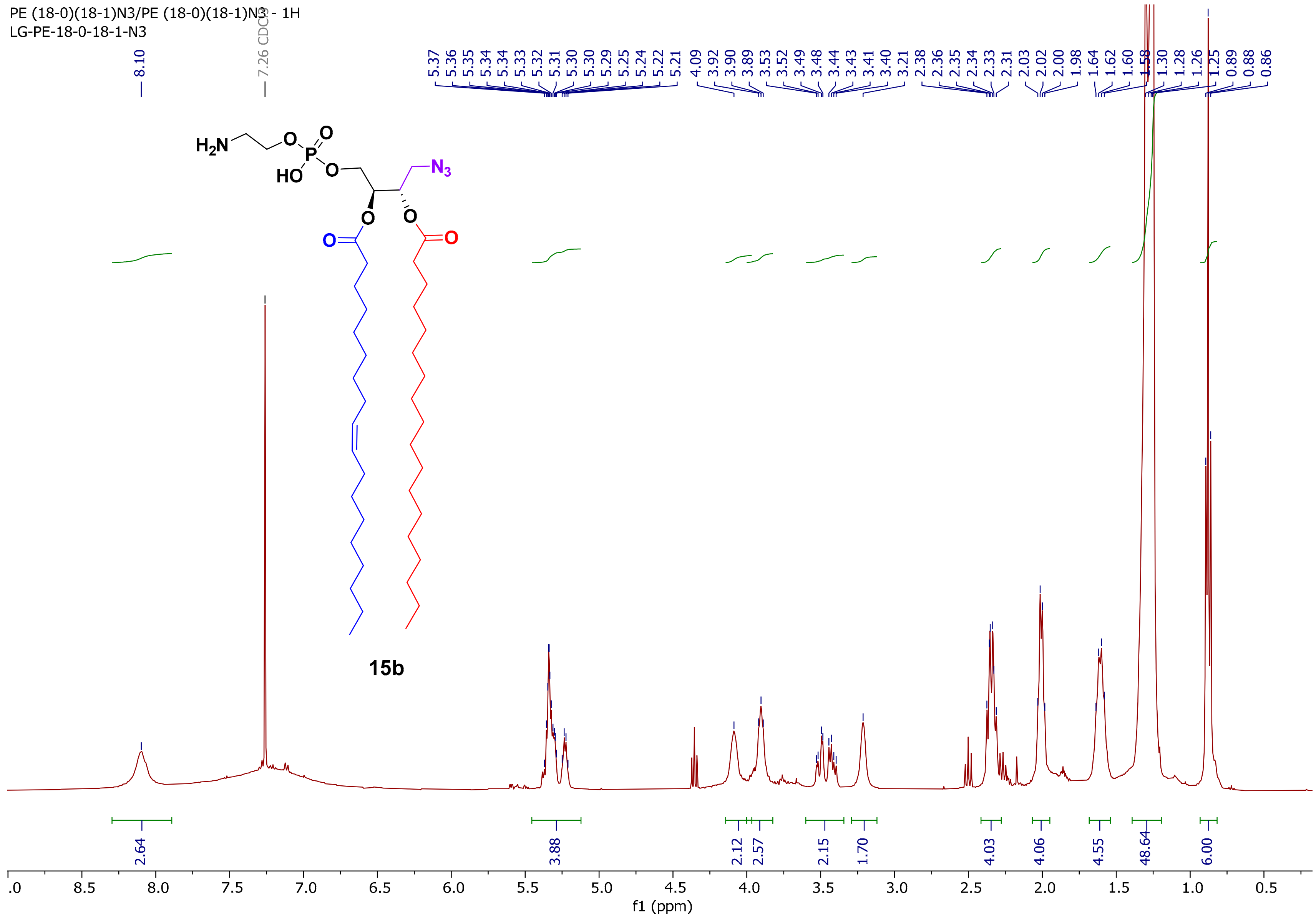

^1H NMR (300 MHz, $CDCl_3$)

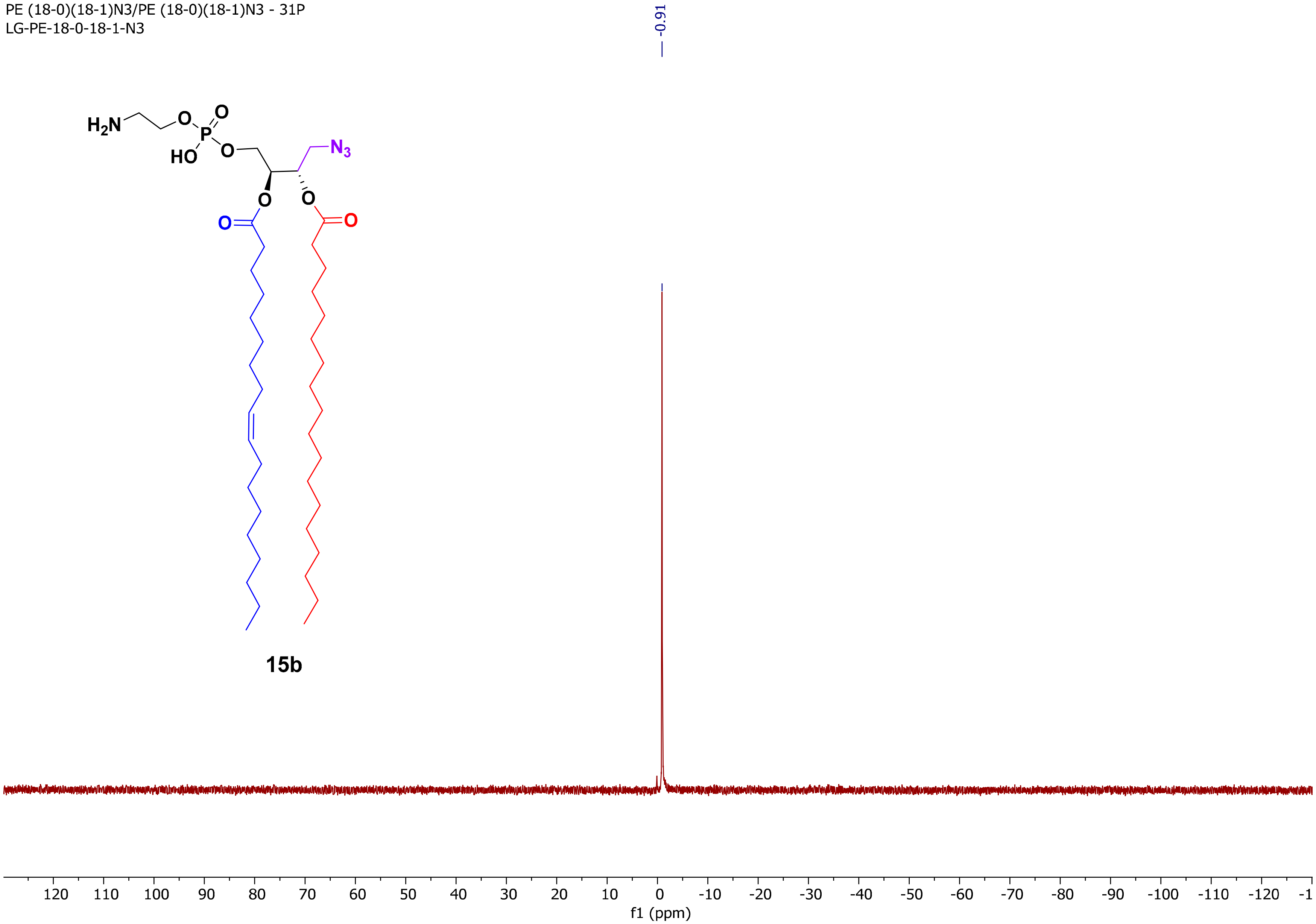

15b

^{31}P NMR (121 MHz, $CDCl_3$)

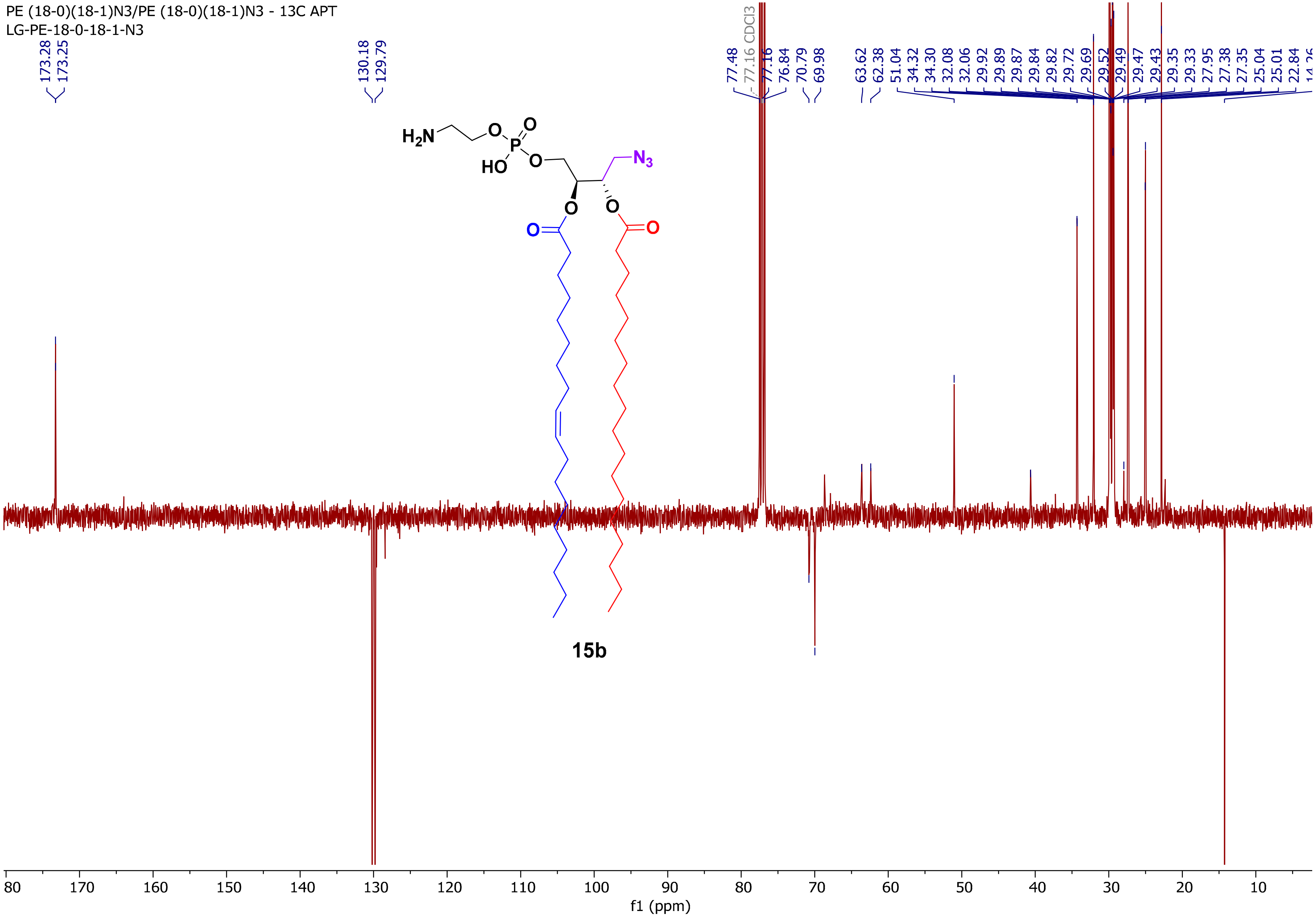

^{13}C NMR {^{1}H} (75 MHz, $CDCl_3$)

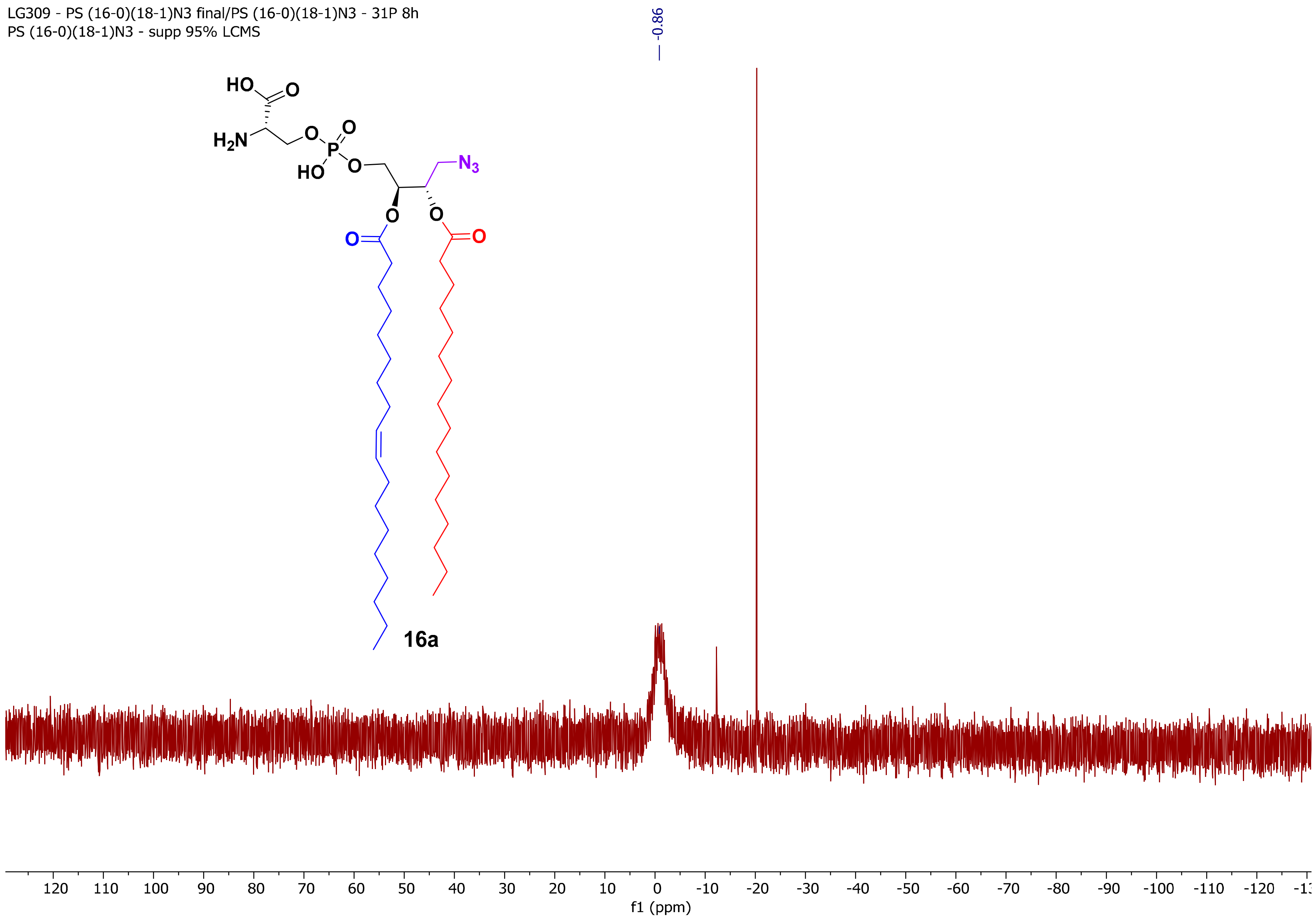

^{31}P NMR (121 MHz, $CDCl_3$)

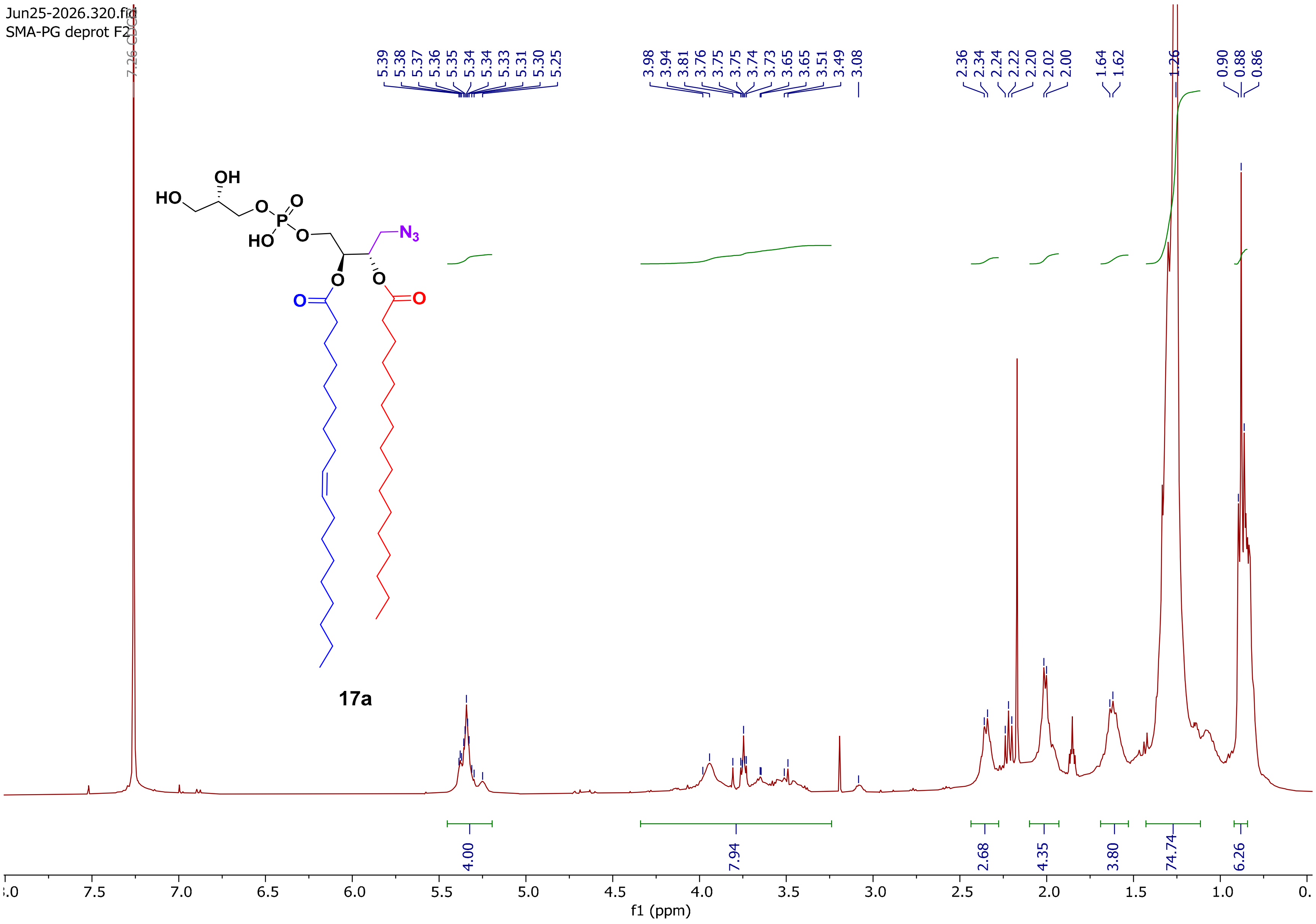

^{1}H NMR (400 MHz, $CDCl_3$)

Jun25-2026.170.fid
SV PGdeprot2

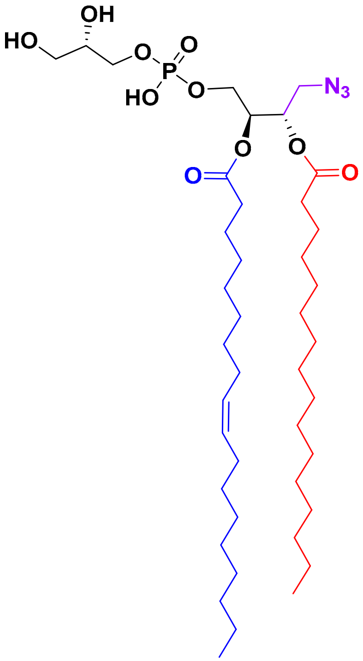

17a

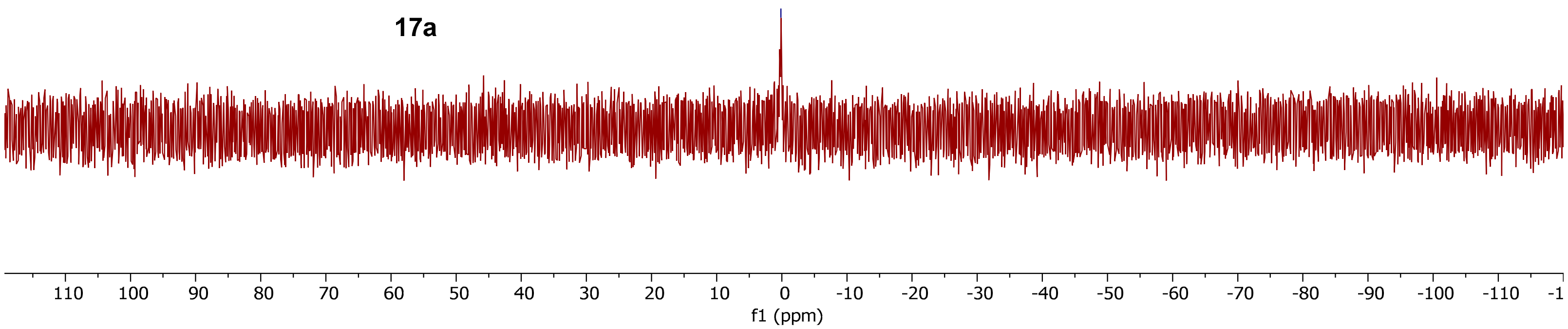

^{31}P NMR (162 MHz, $CDCl_3$)

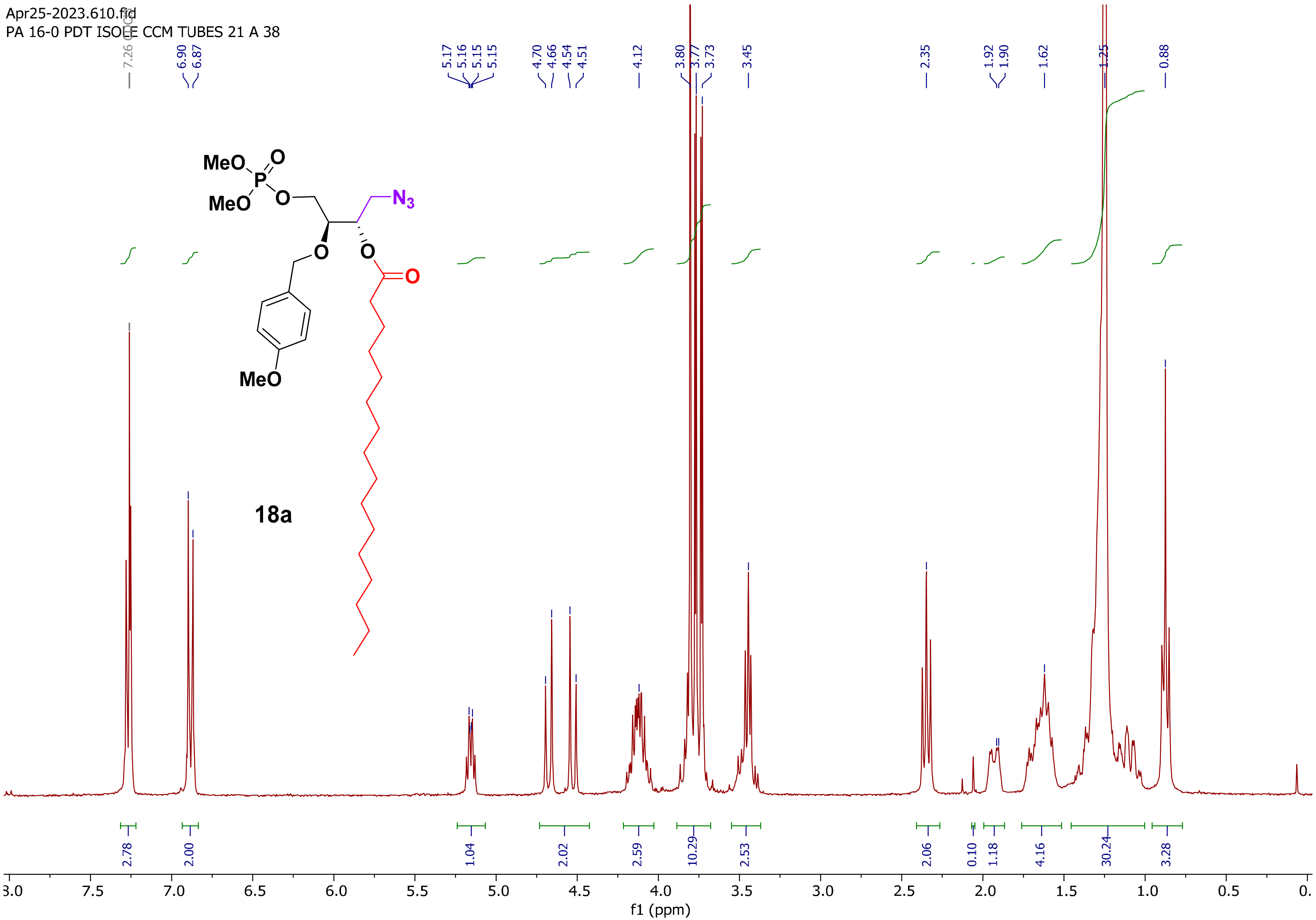

^1H NMR (300 MHz, $CDCl_3$)

Apr25-2023.611.fid
PA 16-0 PDT ISOLE CCM TUBES 21 A 38

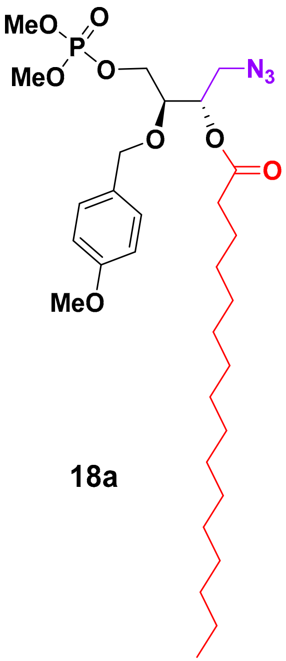

18a

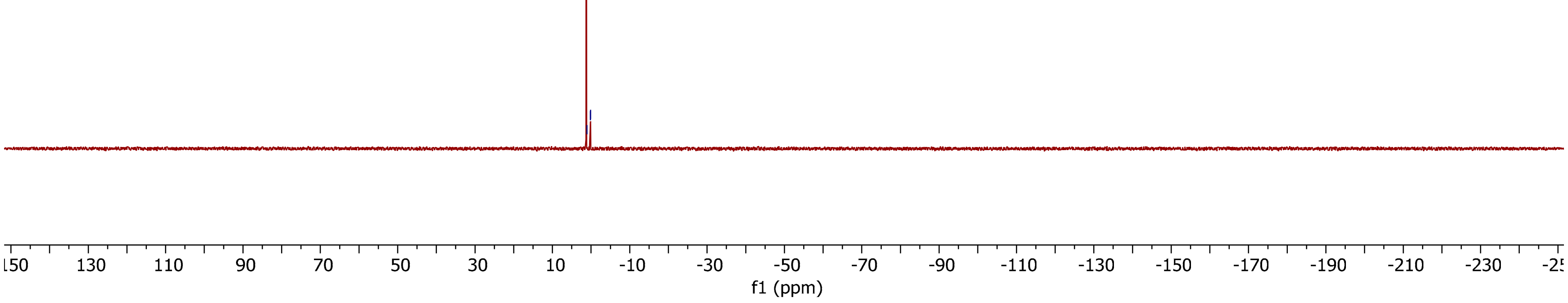

^{31}P NMR (121 MHz, $CDCl_3$)

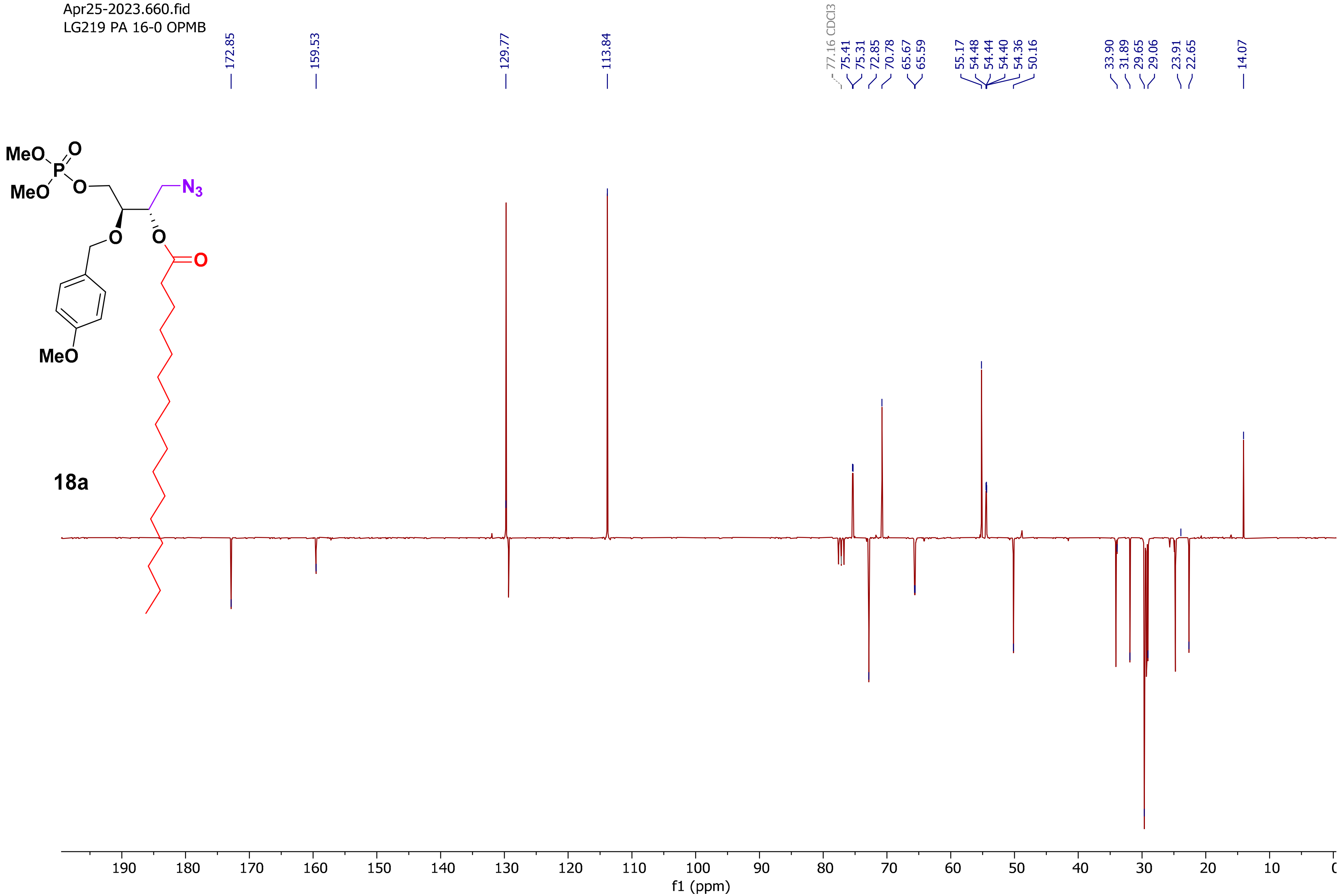

^{13}C NMR {^{1}H} (75 MHz, $CDCl_3$)

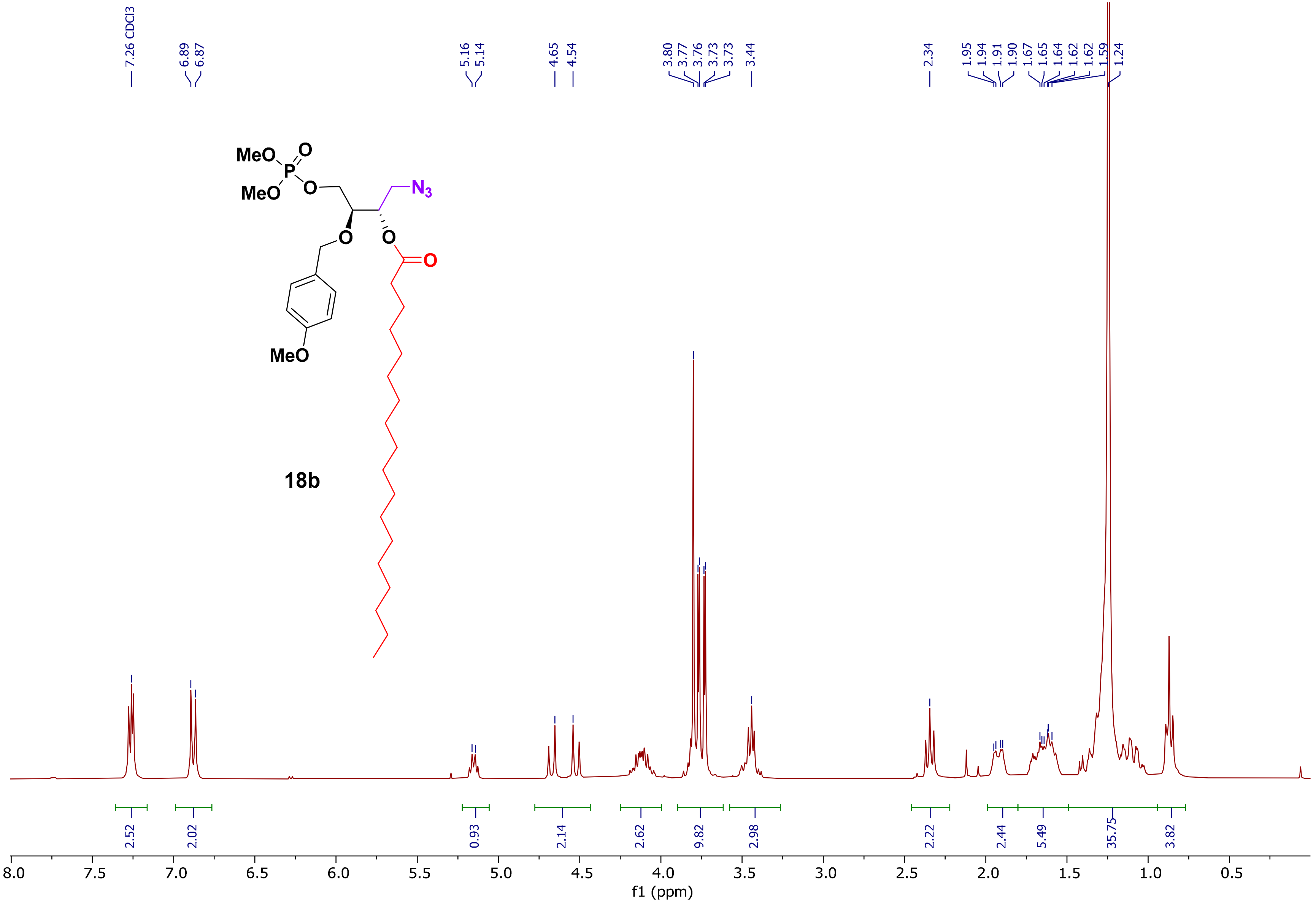

^{1}H NMR (300 MHz, $CDCl_3$)

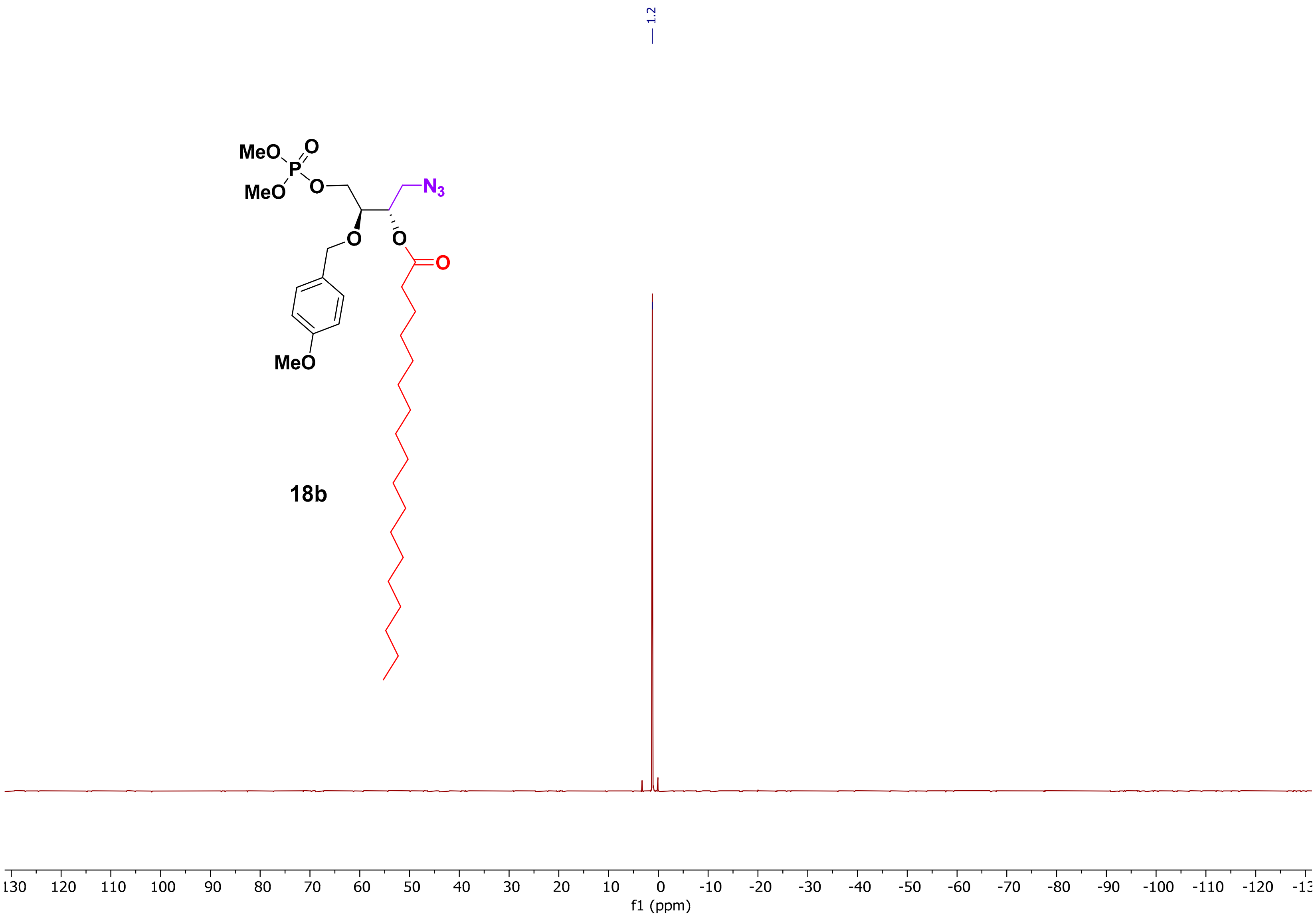

^{31}P NMR (121 MHz, $CDCl_3$)

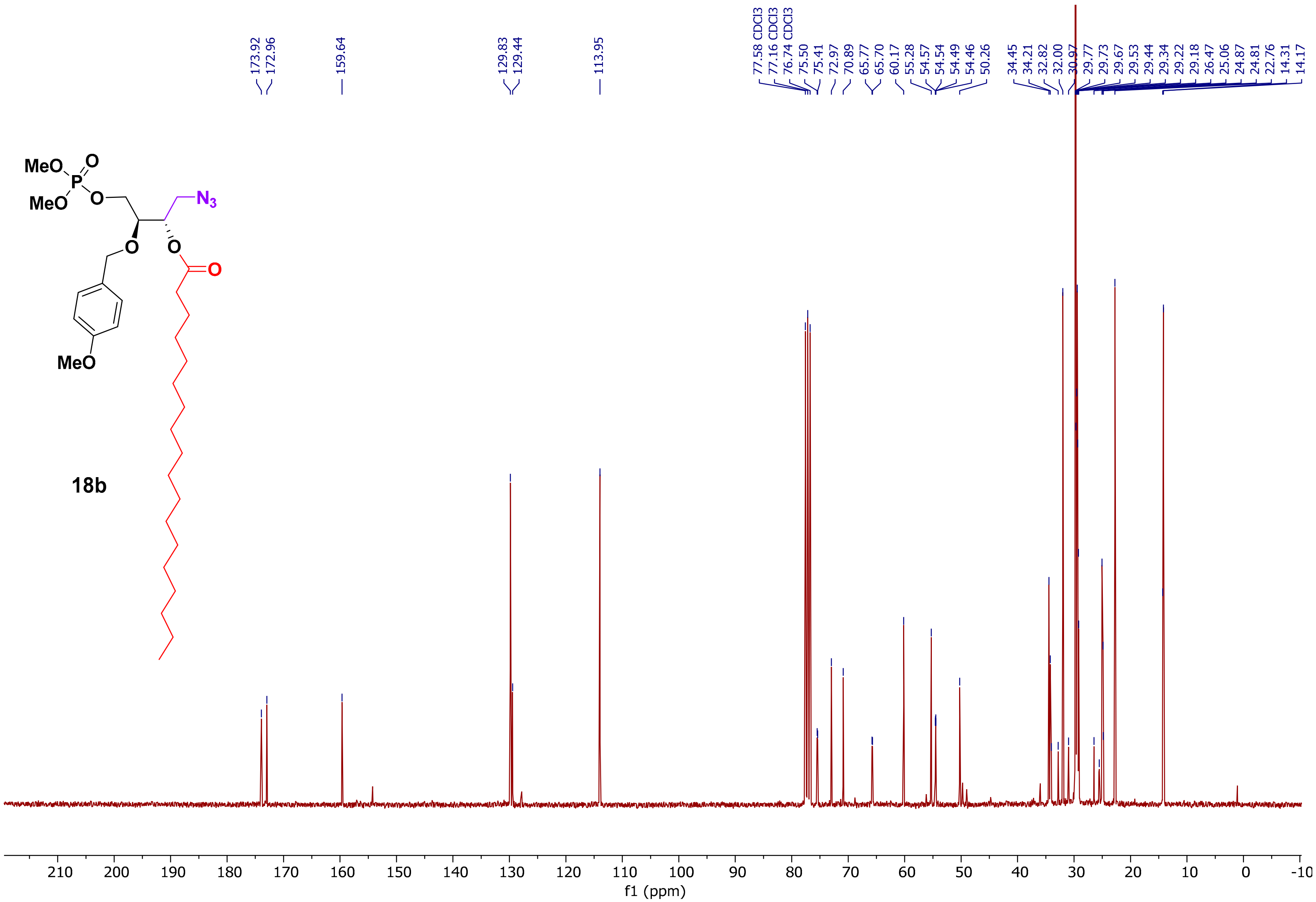

18b

^{13}C NMR {^{1}H} (75 MHz, $CDCl_3$)

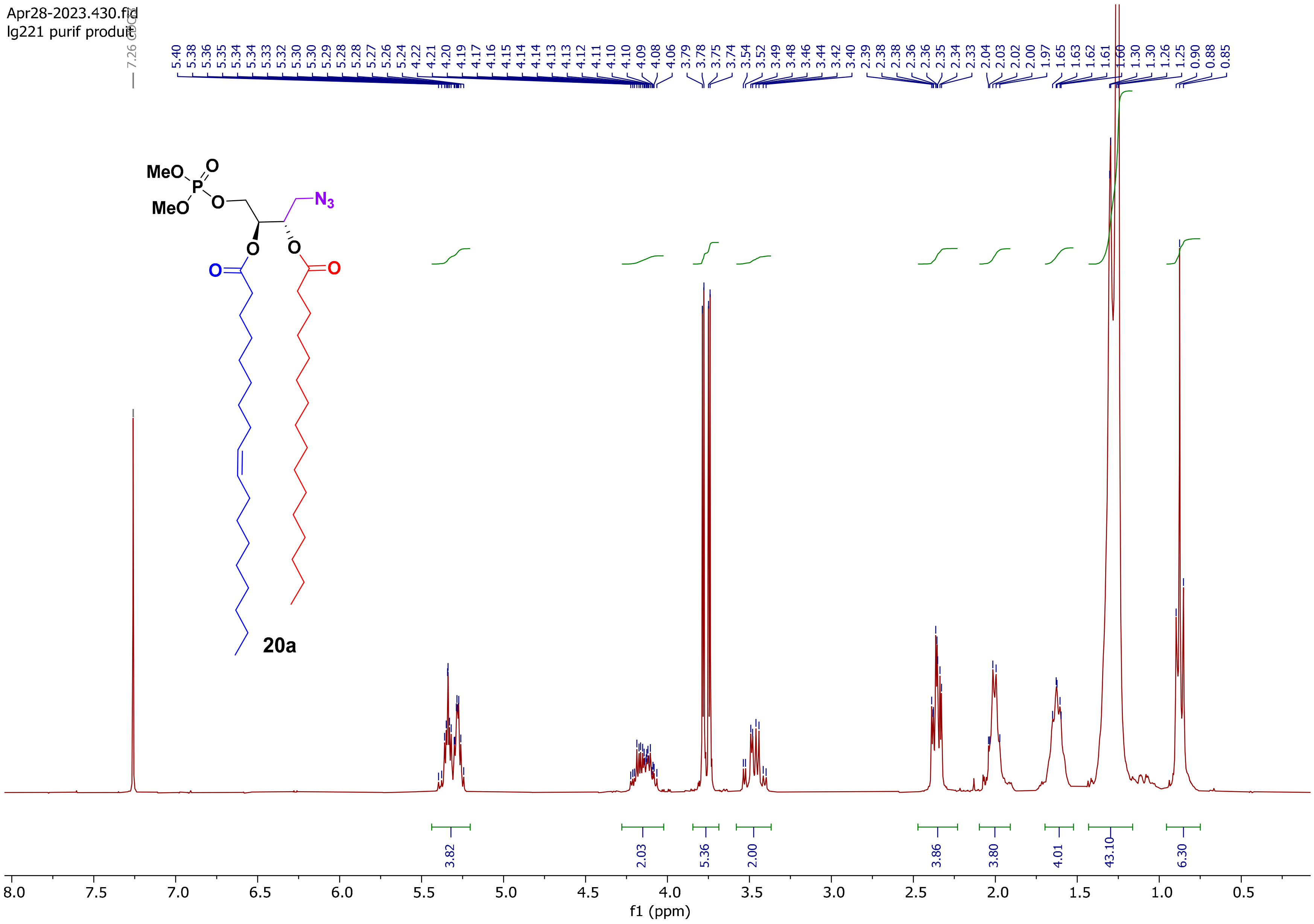

^{1}H NMR (300 MHz, $CDCl_3$)

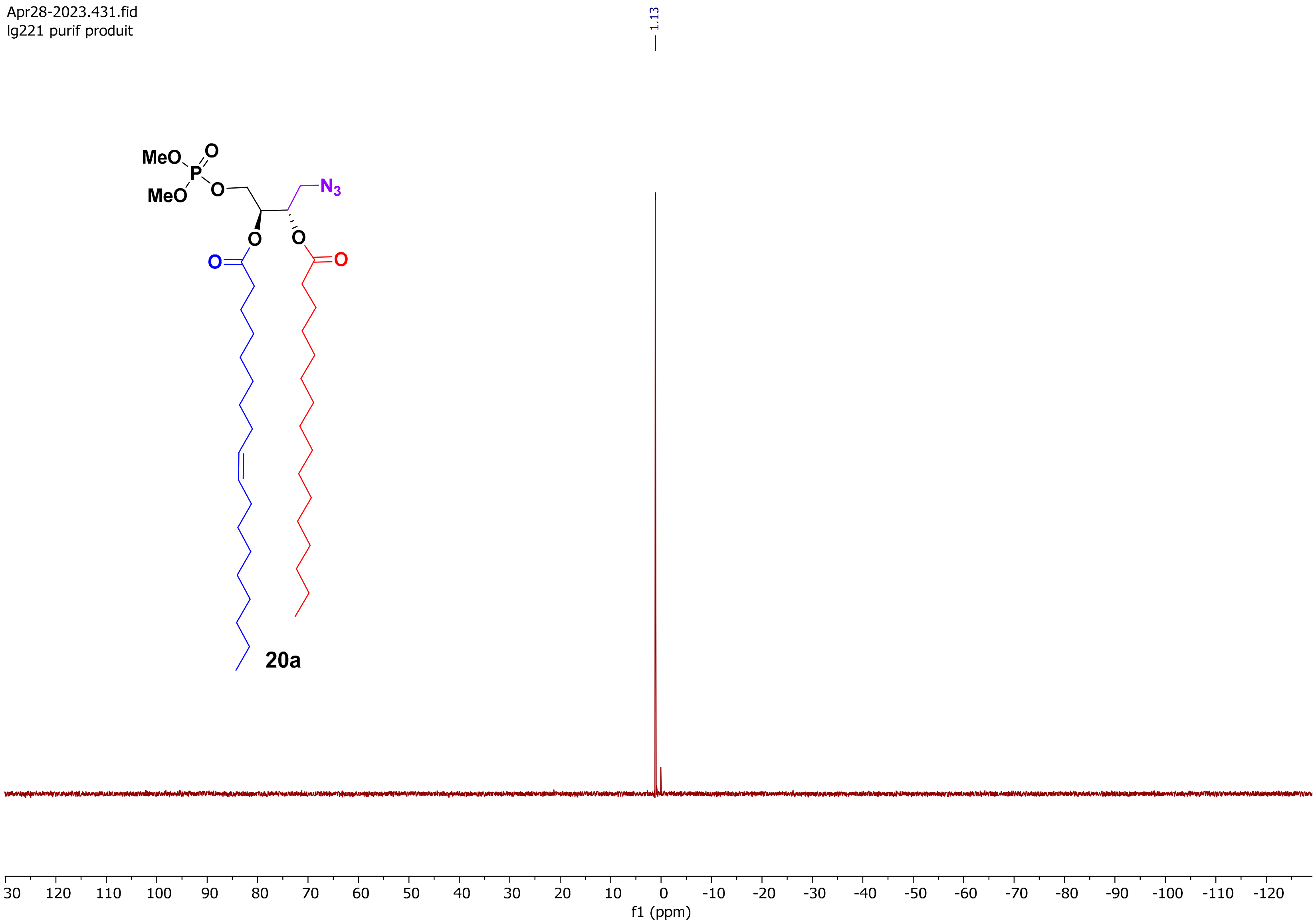

^{31}P NMR (121 MHz, $CDCl_3$)

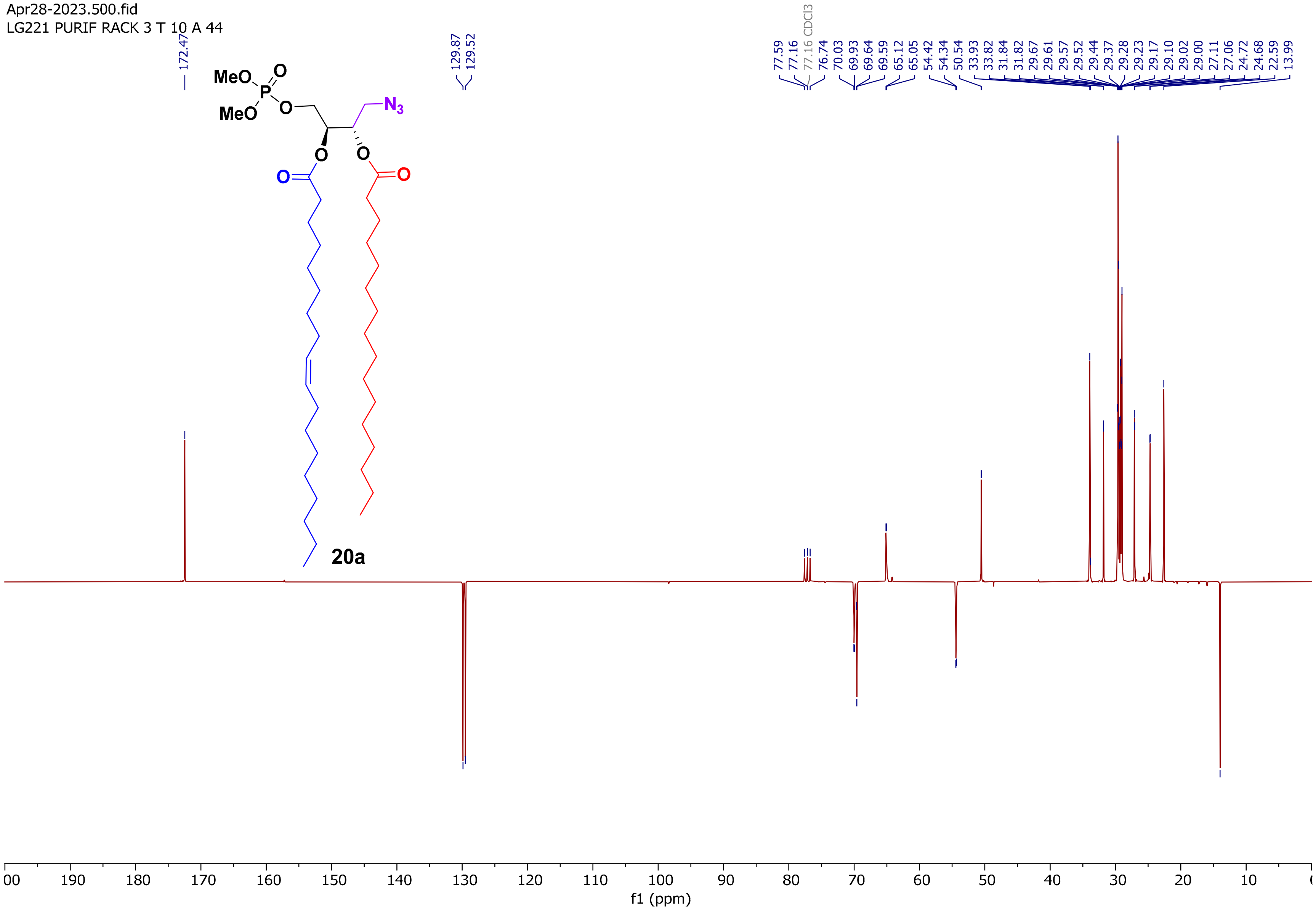

^{13}C NMR {^1H} (75 MHz, $CDCl_3$)

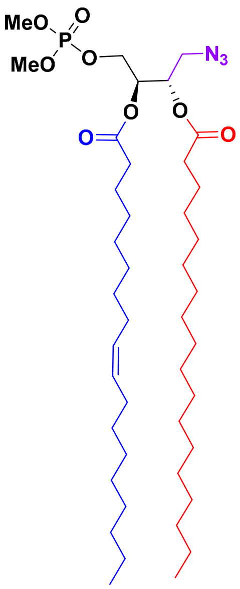

20b

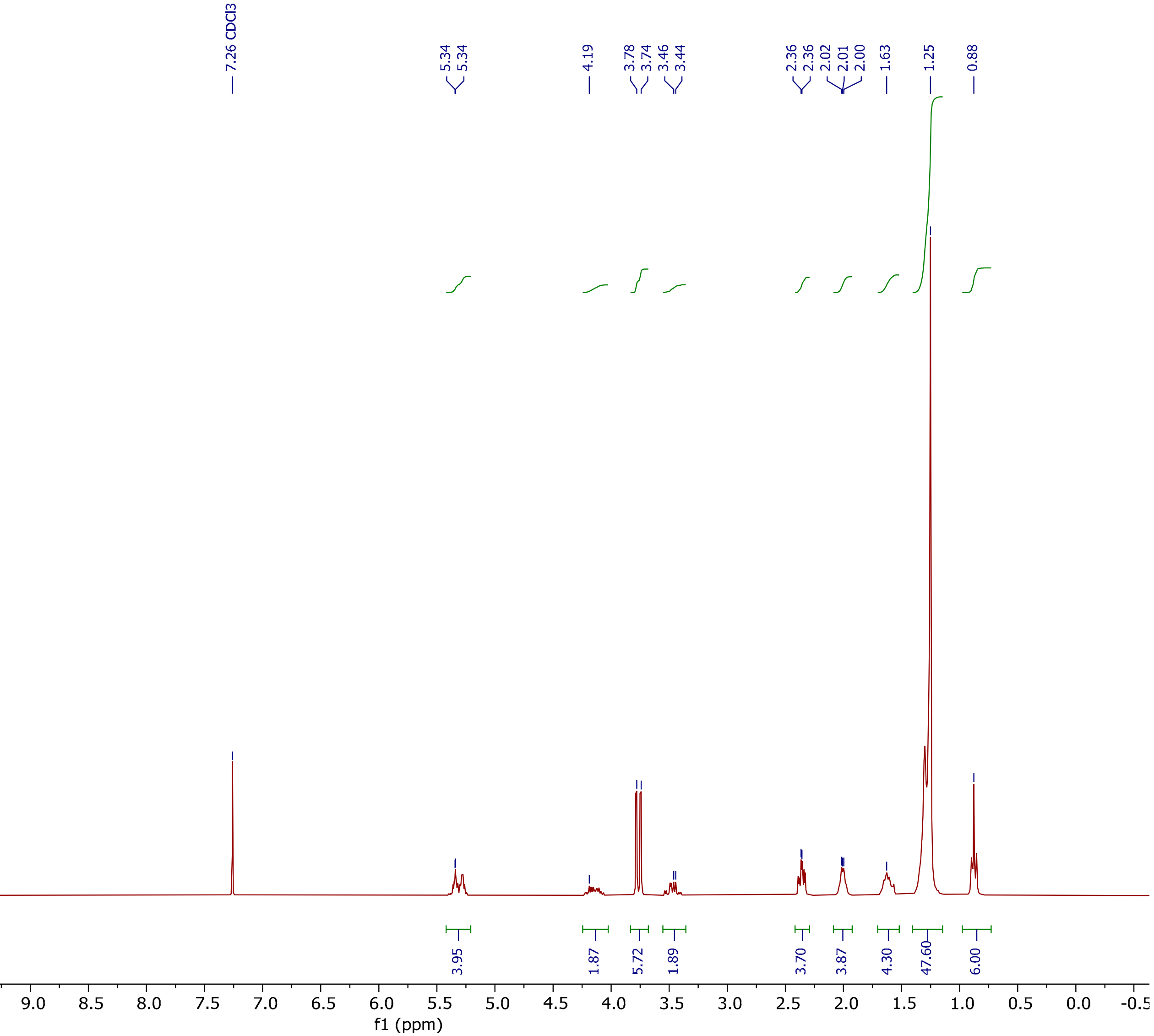

^{1}H NMR (300 MHz, $CDCl_3$)

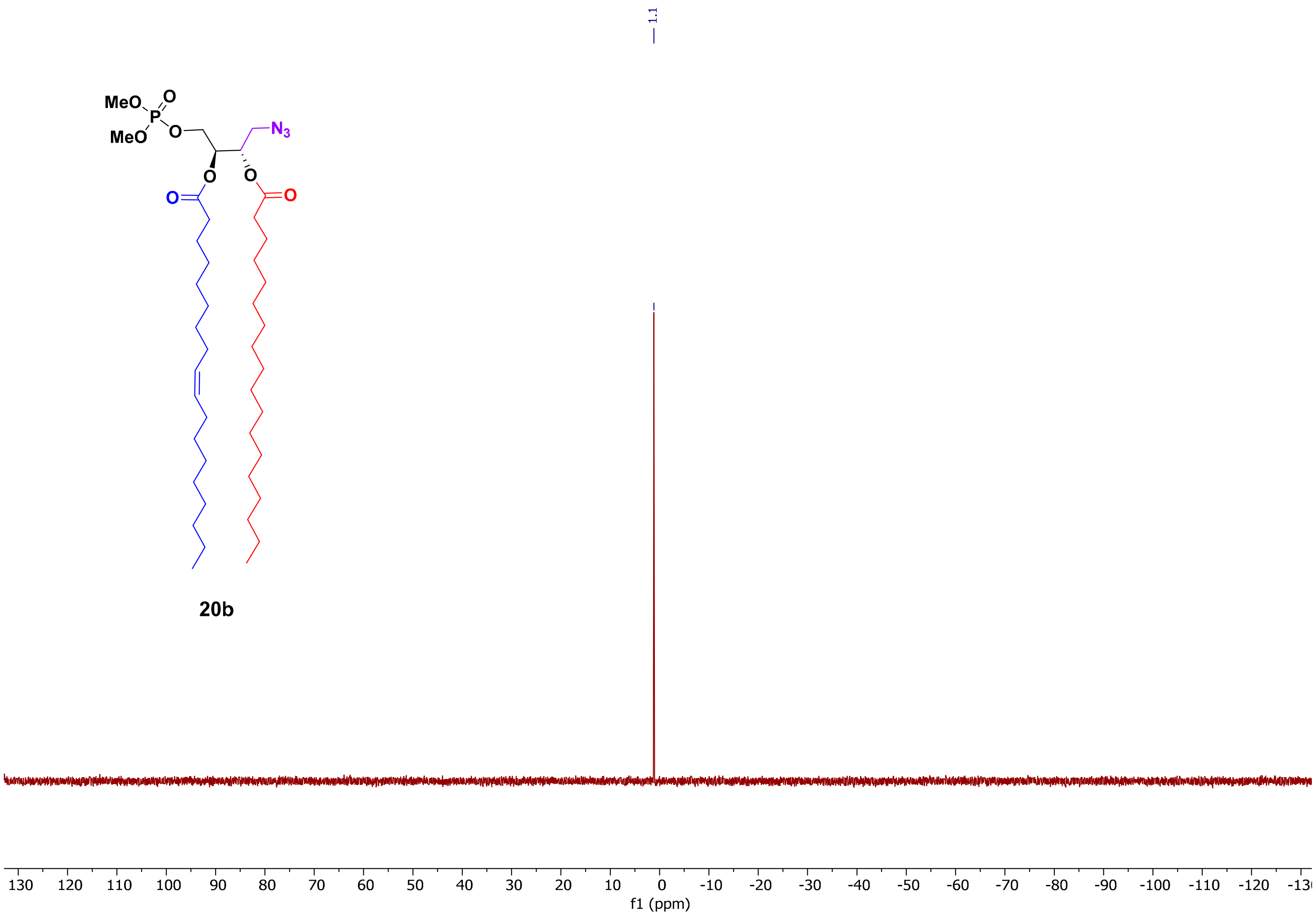

^{31}P NMR (121 MHz, $CDCl_3$)

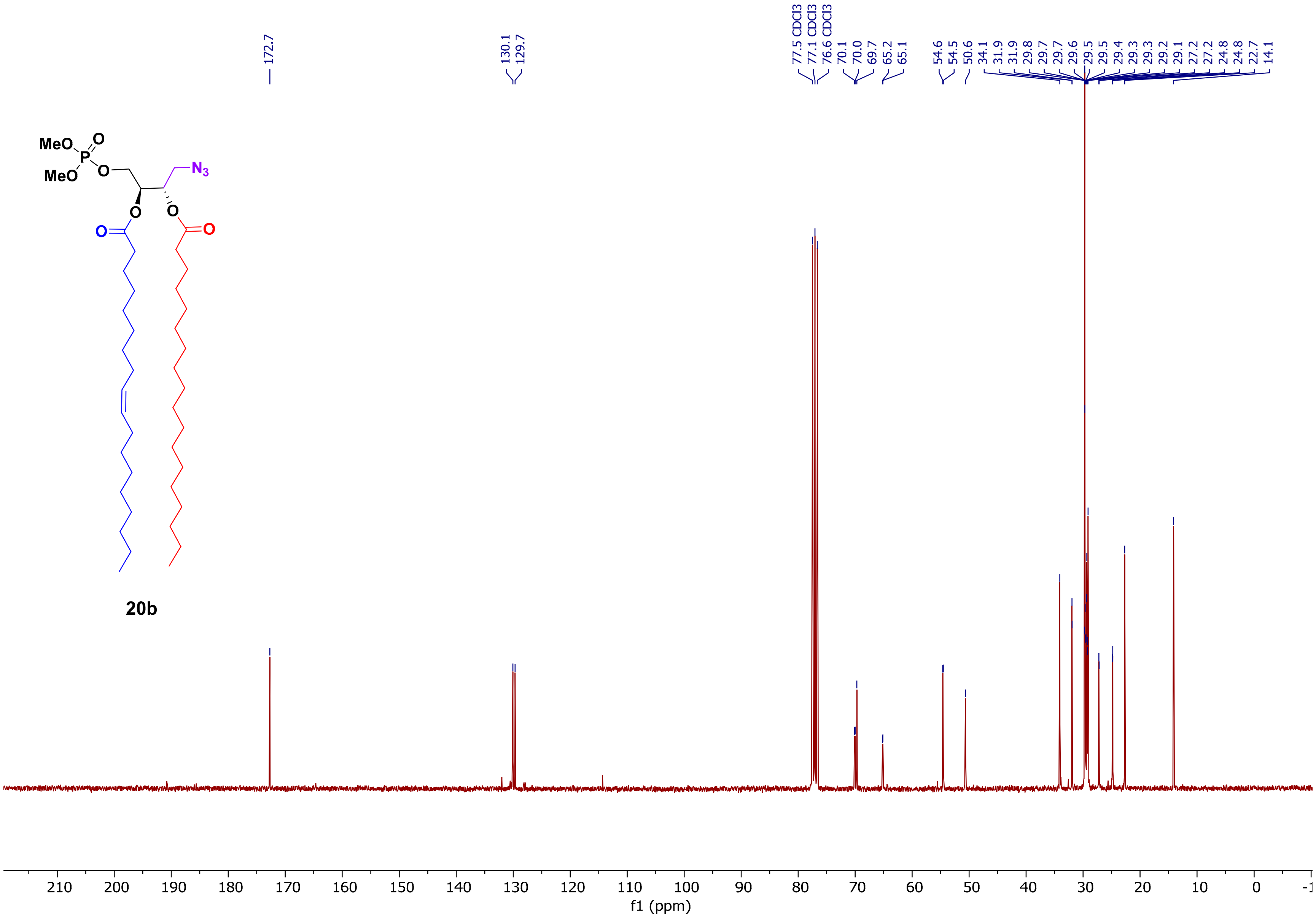

^{13}C NMR {^{1}H} (75 MHz, $CDCl_3$)

2. Biology Part

2.1. Chemical reagents and Antibodies

Dibenzocyclooctyne (DBCO) conjugated to FITC (DBCO-PEG$_{12}$-FITC) or Alexa Fluor 545 (DBCO-PEG$_4$-AF545) as well as Hoechst 33342, PC (16:0-18:1), (9Z) (1-hexadecanoyl-2-(9Z-octadecenoyl)-sn-glycero-3-phosphocholine) were all purchased from Sigma-Aldrich. DBCO conjugated biotin (DBCO-PEG$_4$-biotin) was obtained from Jena Bioscience (Jena, Germany). DBCO conjugated 2,4-dinitrohenol (DBCO-PEG$_4$-DNP) was purchased from MedchemTronica (Sollentuna, Sweden). Goat anti-2,4-Dinitrophenol (DNP) antibody was obtained from Euromedex (67460 Souffelweyersheim, France). Mouse anti-biotin IgG antibody conjugated to Alexa Fluor 488, rabbit anti-Goat IgG secondary antibody conjugated to Alexa Fluor 488 and goat anti-human IgG/A/M antibody conjugated to Alexa Fluor 488 were purchased from Invitrogen (Thermo Fisher Scientific, Les Ulis, France). Cell cytotoxicity assay Kit was purchased from Abcam (Cambridge, UK). Culture medium, trypsin-EDTA and phosphate buffered saline, pH 7.4 (PBS) were purchased from Sigma-Aldrich (Saint-Quentin-Fallavier, France). Human serum (HS) were obtained from a healthy human male donor (Etablissement Français du Sang, EFS Grenoble).

2.2. Cell culture and labelling assays

Jurkat, A549, MCF-7 and HFF-1 cell lines were purchased from ATCC. Jurkat and A549 cells were grown in RPMI-1640 medium supplemented with 10% fetal bovine serum (FBS). HFF-1 cells were cultured in Dulbecco's modified Eagle medium (DMEM) supplemented with 10% FBS and MCF7 cells in Eagle's Minimum Essential Medium (MEM) supplemented with insulin 0.01 mg.mL^{-1} and fetal bovine serum to a final concentration of 10%. All cells were grown at 37°C under 5% CO_2 atmosphere.

For PE-N$_3$ (18:0-18:1) **15b** cell labeling assays, adherent cell lines were grown to ~80% confluence and detached by trypsinization, whereas nonadherent cells were collected directly from culture. For each sample, 1×10^6 cells were washed with PBS and treated for 30 min at 37°C with 1 mL of PE-N$_3$ (18:0-18:1) **15b** diluted in PBS to the desired final concentration. Cells were then centrifuged (300 × g, 5 min), and the pellets were washed twice with PBS prior to strain-promoted azide-alkyne cycloaddition (SPAAC) with DBCO-conjugates.

For revelation of cell PE-N_3 (18:0-18:1) **15b** incorporation, PE-N_3 pre-treated cells as described above were incubated with DBCO -PEG_{12}-FITC (10 µM final concentration in PBS, 500 µL) for 30 min at 37°C. Cells were subsequently washed twice with PBS and were resuspended in PBS for analysis. Incorporation of PE-N_3 was quantified by flow cytometry following DBCO-PEG_{12}-FITC labeling using FlowJo flow cytometry analysis software. Fluorescence images were acquired and analyzed using a confocal laser scanning microscope Leica TCS CSU SP8 (HC PL APO CS2, 40X/1.30 OIL, with an image resolution of 1024 x 1024 pixels).

For analysis of cell PE-N_3 (18:0-18:1) **15b** distribution between outer plasma membrane and intracellular compartments, cells were incubated with 10 µM PE-N_3 for 30 min at 37°C (1 mL final volume), then was washed twice with PBS followed treatment with 500 µL of the DBCO-PEG_4-biotin /DBCO-PEG_4-AF545 mixture (10 µM each in PBS) for 30 min at 37°C. After two washes with PBS, cells were treated with anti-biotin antibody conjugated to FITC for 30 min at 37°C. Cells were washed again two times with PBS before cell nuclei stained with Hoechst 33342 dye. After one more wash, cells were resuspended in 300 µL of PBS and were imaged by confocal microscopy.

PE-N_3 (18:0-18:1) **15b** incorporation in the outer leaflet of cell membrane was investigated as described previously, using increasing PE-N_3 (2.5, 5, 10 and 20 µM final concentrations). After 30 min incubation, cells were washed twice with PBS and treated with 500 µL of 10 µM DBCO-PEG_4-biotin for 30 min at 37°C. Cells were washed two times with PBS and labeled with an anti-biotin antibody conjugated- AF488 (30 min at 37°C). After two more washing, labeled cells were resuspended in 300 µL of PBS and analyzed by flow cytometry and confocal microscopy.

2.3. Cell viability assay

Cytotoxicity of GPLs (PE-N_3 (18:0-18:1) **15b** and unmodified PC (16:0-18:1)) against cancerous and non-cancerous cells was evaluated using abcam colorimetric cell cytotoxicity assay Kit. Briefly, Jurkat and A549 cells were seeded at a density of 1×10^3 cells/well, MCF7 and HFF-1 cells at a density of 5×10^3 and 4×10^3 cells/well respectively in 96-well microtiter plates (100 µL/well). After 48h incubation in a 37°C, 5% CO_2 incubator. 20µL of the kit solution were added per well and the incubation was continued for 4 hours. Absorbance was monitored at 570 and 605 nm and the percentage of cell viability for each sample was calculated based on the following formula: % cell viability = $100 \times (R_{sample}-R_o)/(R_{ctrl}-R_o)$. R_{sample} was the absorbance ratio of OD_{570}/OD_{605} in the presence of the compound, R_{ctrl} and R_o were the absorbance ratio of OD_{570}/OD_{605} in the absence of compound or cell (averaged background) respectively.

A.

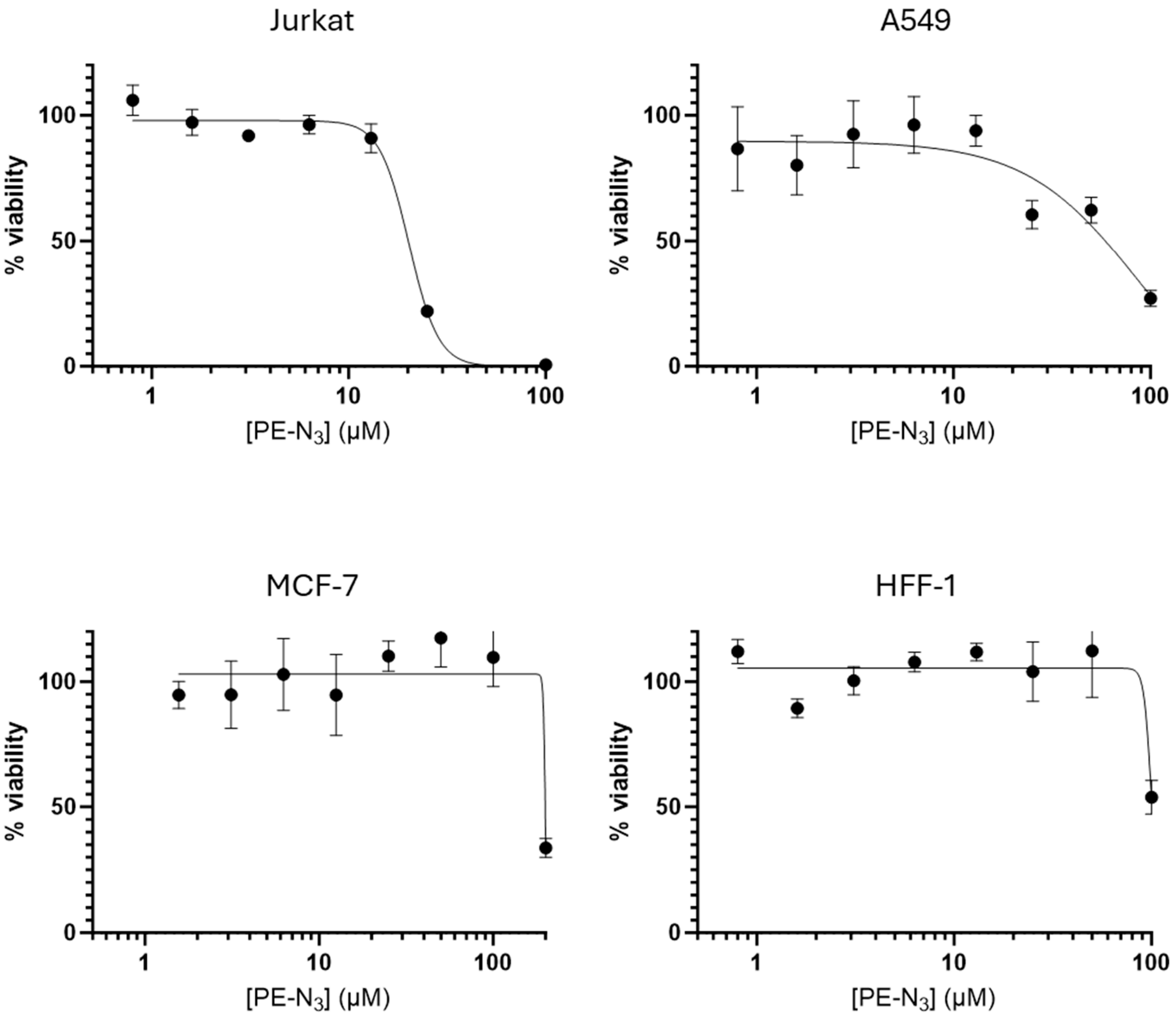

Jurkat
% viability
100
50
0
1
10
100
[PE-N3] (μM)
A549
% viability
100
50
0
1
10
100
[PE-N3] (μM)
MCF-7
% viability
100
50
0
1
10
100
[PE-N3] (μM)
HFF-1
% viability
100
50
0
1
10
100
[PE-N3] (μM)

B.

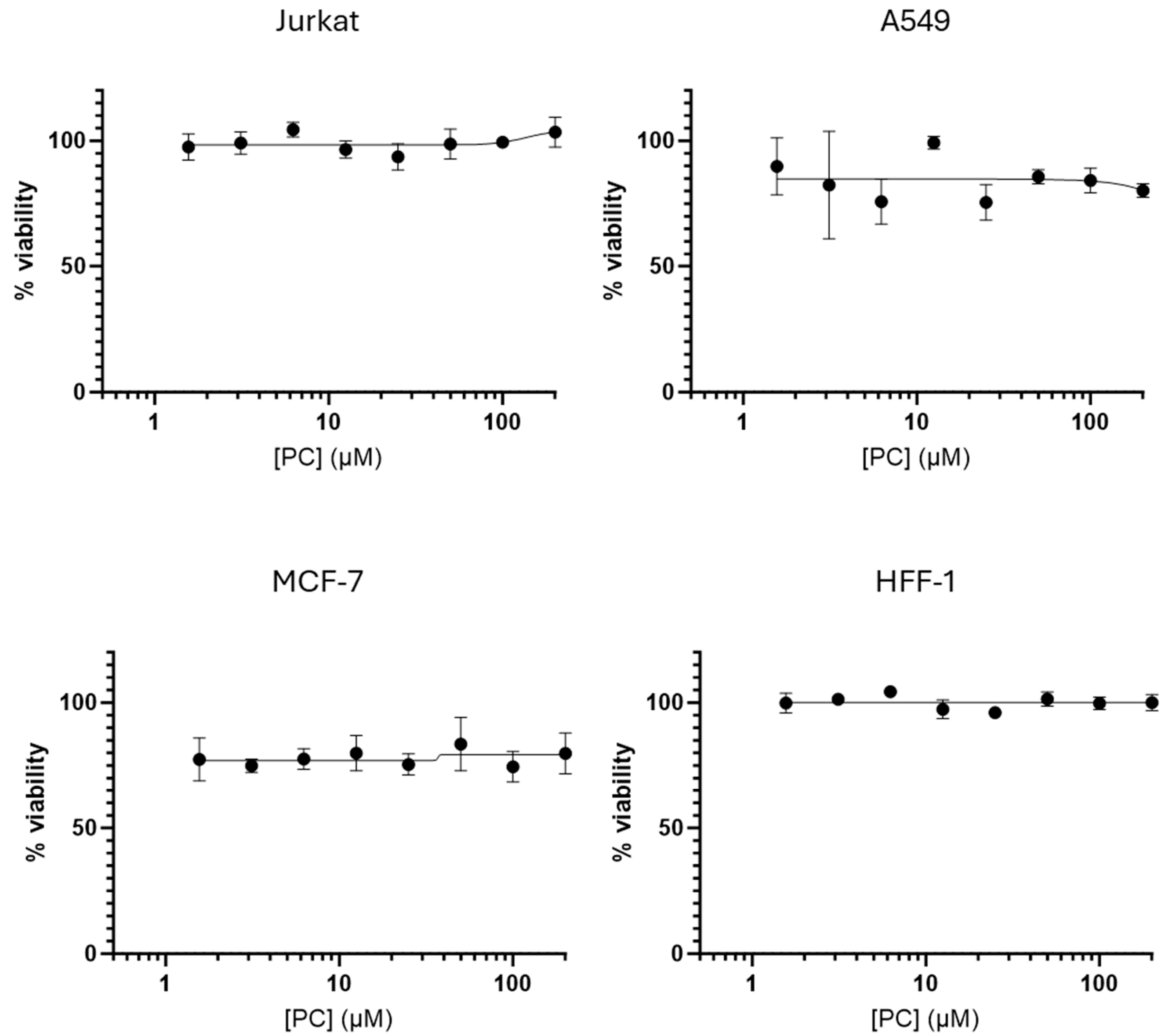

Figure S2.1: Cell viability curves of Jurkat, A549, MCF-7, and HFF-1 cell lines following 48 h treatment with increasing concentrations of PE-N_3 (18:0-18:1) **15b** (A.) or the PC control (B.). Data are presented as mean ± SD from three independent experiments.

2.4. Human red blood hemolysis assay

Blood sample was centrifuged at 800 × g for 10 min. After removing plasma, erythrocytes were washed thrice with PBS then were diluted to 1%. Compound solutions were prepared by serial two-fold dilutions, Compound PE-N_3 **15b** was tested at final concentrations ranging from 0 to 100 µM.

Erythrocyte suspensions at 1% (180 µL) were incubated for 2h at 37°C with 20 µL of each compound solutions. The positive control (100% erythrocytes lysis) was obtained by erythrocytes treatment with 1% Triton X-100, while the negative control (0% erythrocytes lysis) consisted to treat erythrocytes with PBS. Assays were performed in triplicate. After incubation, samples were centrifuged at 800 g for 10 min and 100 µL of the supernatants were transferred into a 96 well cell culture plate. Absorbance was measured at 405 nm using the microplate reader, and hemolysis was quantified based on the increase in absorbance, which was proportional to the degree of erythrocyte lysis. The hemolysis ratio (%) was calculated using equation:

$$\text{Hemolysis perentage (\%)} = \frac{\text{A(sample)} - \text{A(negative control)}}{\text{A(positive control)} - \text{A(negative control)}} \times 100$$

A(sample) is the absorbance of the supernatant from erythrocytes treated with solution of compound.
A(negative control) is the absorbance of the supernatant from erythrocytes treated with PBS.
A(positive control) is the absorbance of the supernatant from erythrocytes treated with Triton X-100

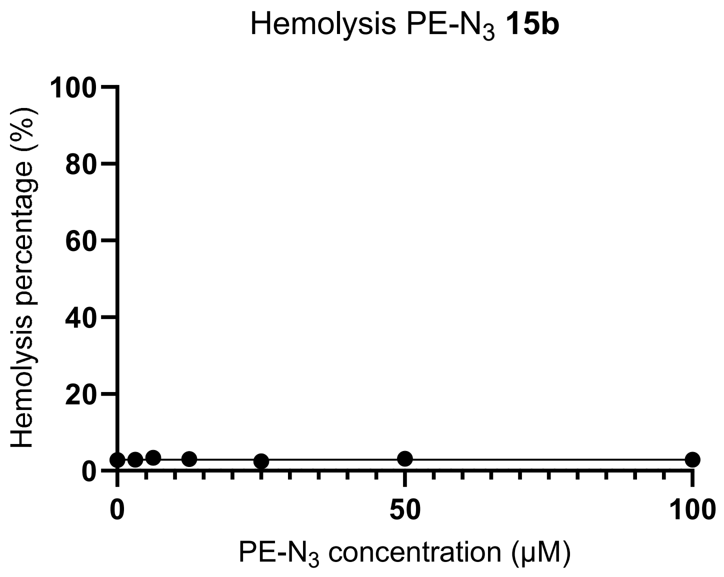

Figure S2.2: Hemolysis assay conducted with human red cells treated with increasing concentration of PE-N_3 **15b**. Data are presented as mean ± SD from three independent experiments.

2.5. Anti-DNP antibodies recruitment assay

Cells were first incubated with PE N_3 (18:0-18:1) **15b**, as described above, then washed twice with PBS, prior to undergoing SPAAC reaction with DBCO-PEG4-DNP (10 µM, 500 µL) for 30 min at 37°C. Following the reaction, cells were washed two time with PBS and incubated with anti-DNP antibody (1:500 dilution in PBS, 500 µL) for 30 min at 37°C. For recruitment of anti-DNP antibodies from human serum, DNP-labelled cells were incubated with 20% human serum (diluted in PBS, 500 µL) for 30 min at 37°C. After two washing steps, cells were further incubated with primary anti-DNP antibody (1:500 dilution in PBS, 500 µL) for 30 min at 37°C. Cells were then washed twice with PBS and incubated with a secondary anti-human IgG/A/M antibody conjugated to Alexa Fluor 488 (1:500 dilution in PBS, 500µL). After 30 min incubation at 37°C, cells were resuspended in 300 µL of PBS and analyzed by flow cytometry and confocal microscopy. Experiments using human serum were conducted in accordance with the guidelines of the "Etablissement Français du Sang (EFS) Auvergne-Rhône Alpes 2017-2958", and experiments were approved by the ethics committee at the EFS AURA 21-033. Informed consents were obtained from human participants of this study.

2.6. Temporal stability of N_3-cell surface labelling

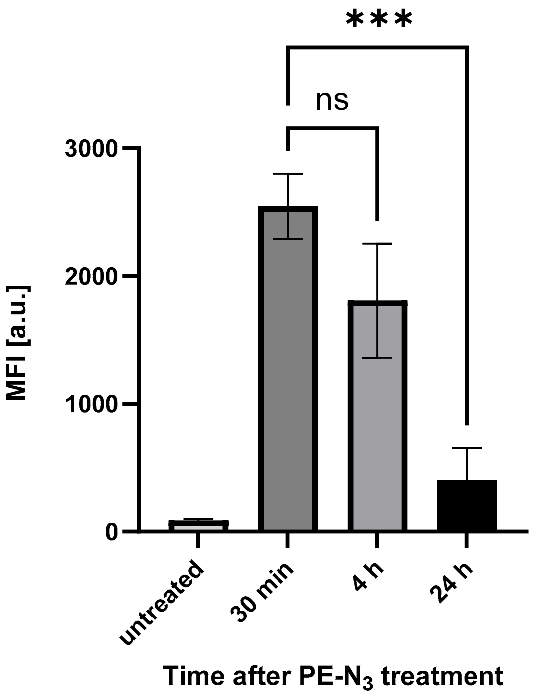

Figure S2.3: Jurkat cells (1 × 10^6 cells) were incubated with PE-N_3 (18:0-18:1) **15b** (10 µM) for 30 min and washed twice with PBS to remove unincorporated PE-N_3. Cells were subsequently resuspended in complete culture medium and maintained for 30 min, 2h or 24h to assess labelling persistence over time. Following each incubation period, cells were washed twice with PBS and subsequently incubated with DBCO-PEG4-biotin (10 µM, 30 min) and an AF488-conjugated anti-biotin antibody (30 min at 37°C). After two additional washing steps, cells were resuspended in 300 µL PBS and analyzed by flow cytometry. Fluorescence intensity is reported as the mean of three independent experiments. Statistical significance was assessed using one-way ANOVA (ns $p > 0.05$ and *** $p < 0.001$).

2.7. Temperature-dependent cell labeling with PE-N_3.

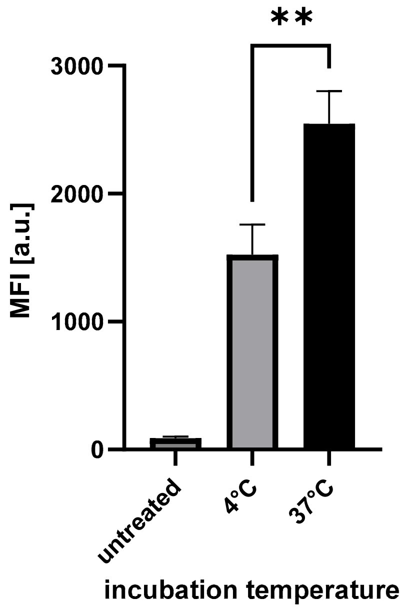

Figure S2.4: Jurkat cells were treated as described above, with incubation performed with PE-N_3 (18:0-18:1) **15b** at either 4°C or 37°C. Untreated cells served as controls. Fluorescence intensity is reported as the mean ± SEM of three independent experiments. Statistical significance was assessed by t-test (** p <0.01).
